# Organoid modeling of human fetal lung alveolar development reveals mechanisms of cell fate patterning and neonatal respiratory disease

**DOI:** 10.1016/j.stem.2022.11.013

**Published:** 2022-12-08

**Authors:** Kyungtae Lim, Alex P.A. Donovan, Walfred Tang, Dawei Sun, Peng He, J. Patrick Pett, Sarah A. Teichmann, John C. Marioni, Kerstin B. Meyer, Andrea H. Brand, Emma L. Rawlins

**Affiliations:** 1 Wellcome Trust, CRUK Gurdon Institute, University of Cambridge, Cambridge CB2 1QN, UK; 2 Department of Physiology, Development and Neuroscience, University of Cambridge, Cambridge CB2 3DY, UK; 3 Wellcome Trust, MRC Stem Cell Institute, Jeffrey Cheah Biomedical Centre Cambridge Biomedical Campus, Puddicombe Way, Cambridge CB2 0AW, UK; 4 Wellcome Sanger Institute, Hinxton, Cambridge CB10 1SA, UK; 5 European Molecular Biology Laboratory, European Bioinformatics Institute (EMBL-EBI), Wellcome Genome Campus, Cambridge CB10 1SD, UK

## Abstract

Variation in lung alveolar development is strongly linked to disease susceptibility. However, underlying cellular and molecular mechanisms are difficult to study in humans. We have identified an alveolar-fated epithelial progenitor in human fetal lungs, which we grow as self-organizing organoids that model key aspects of cell lineage commitment. Using this system, we have functionally validated cell-cell interactions in the developing human alveolar niche, showing that Wnt signaling from differentiating fibroblasts promotes alveolar-type-2 cell identity, whereas myofibroblasts secrete the Wnt inhibitor, NOTUM, providing spatial patterning. We identify a Wnt-NKX2.1 axis controlling alveolar differentiation. Moreover, we show that differential binding of NKX2.1 coordinates alveolar maturation, allowing us to model the effects of human genetic variation in *NKX2.1* on alveolar differentiation. Our organoid system recapitulates key aspects of human fetal lung stem cell biology allowing mechanistic experiments to determine the cellular and molecular regulation of human development and disease.

## Graphical abstract

**Graphical abstract:**
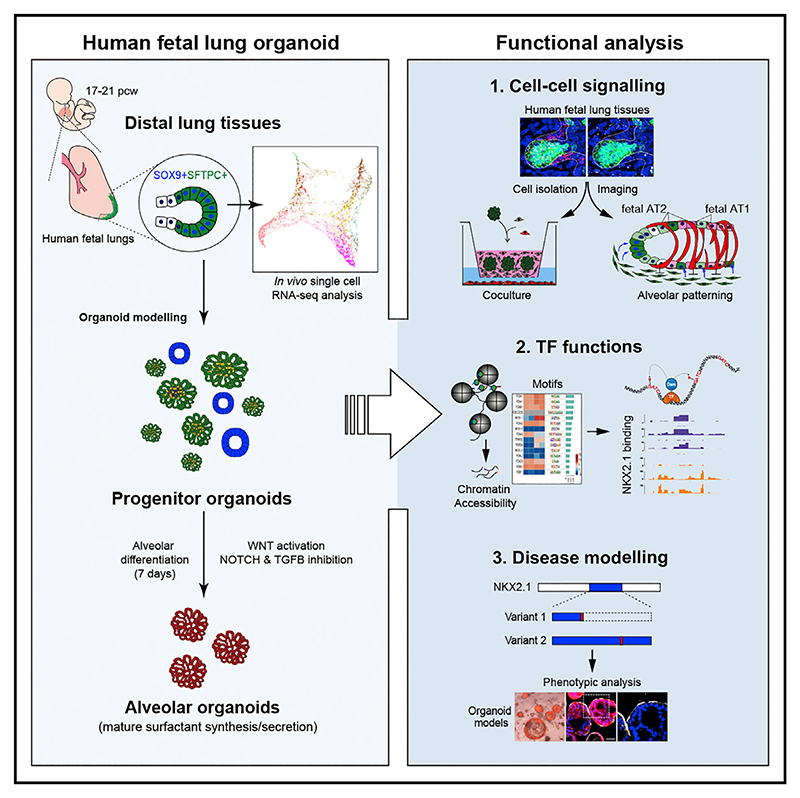

## Introduction

During human lung development the airway tree is formed by branching between ~5 and 16 post-conception weeks (pcw). From ~16 to 26 pcw, the most distal epithelial tubes narrow, come into proximity to capillaries, and start to differentiate as alveolar epithelium.^1,2^ Preterm infants born at more than ~22 pcw have a rudimentary gas exchange surface and can survive if provided with specialized intensive care. However, the molecular mechanisms underlying human alveolar development remain largely unknown.

The human tip epithelium is SOX9/SOX2 dual-positive during airway branching, functions as a multipotent progenitor and has been cultured as organoids.^3,4^ From ~16 pcw, as alveolar differentiation begins, tip progenitors become SOX9 single-positive and more cuboidal in shape.^3^ We hypothesized that growth of tip organoids from 16 to 22 pcw lungs would provide an improved model for studying human alveolar differentiation. In addition, human embryonic lung organoids are amenable to viral transduction and gene editing,^5^ and we speculated that we would be able to obtain genetically manipulable mature human alveolar epithelial cells from such organoids.

Differentiation of human iPSCs to alveolar lineages suggests that Wnt signaling is essential for alveolar fate.^6^ However, Wnt ligands are expressed widely in developing human lungs,^7^ and how spatial patterning of cell fates is achieved is unknown. In mouse lungs, Nkx2.1 is essential for alveolar differentiation and maintenance and binds to promoters of alveolar type 1 (AT1)- and alveolar type 2 (AT2)-cell-specific genes.^8^ Heterozygous missense mutations in the *NKX2.1* homeodomain cause brain**-**lung**-**thyroid syndrome, which includes disrupted surfactant gene expression and interstitial lung disease.^9,10^ However, whether NKX2.1 simply promotes surfactant synthesis,^10^ or has additional roles in human lung alveolar differentiation is unknown.

We find that SOX9^+^ human lung tip progenitors acquire an AT2 gene expression signature by 15 pcw and can grow as self-renewing (SN) organoids that can be readily differentiated. This has allowed us to determine upstream signals and downstream transcription factors (TFs) that promote lineage commitment, identify the role of myofibroblasts in spatial patterning of the developing alveolus and to build models of human neonatal lung disease. This work also provides a source of genetically manipulable human AT2 cells for disease modeling.

## Results

### Human fetal lung tip progenitor cells intrinsically activate alveolar lineage markers from 15 pcw *in vivo*

**Figure 1:**
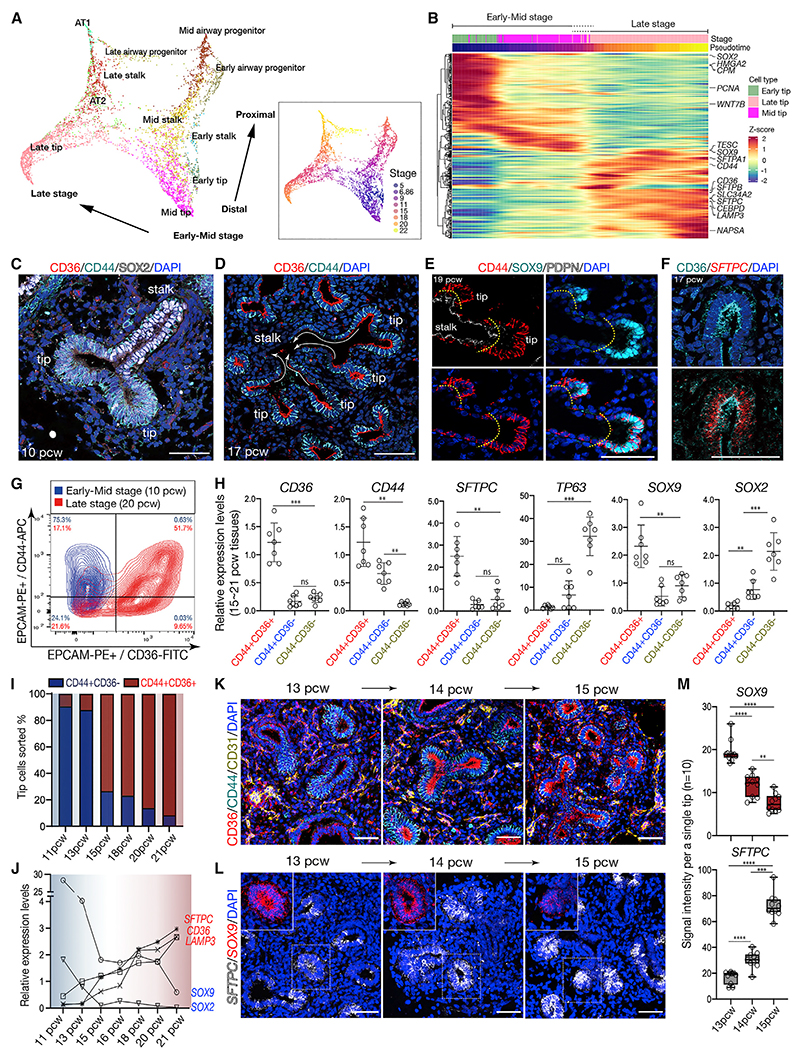
Human fetal lung tip progenitor cells acquire alveolar features during normal development (A) Force-directed embedding of single-cell transcriptomes of cells derived from distal human lung tissues from 5 to 22 pcw, by cell types and stages. (B) Trajectory heatmap showing differential marker gene expression among early, mid, and late-stage tip cells. (C–F) Surface antigens, CD44 and CD36, mark tip epithelium at early-mid and late stages. Lungs at 10 (C), 17 (D and F), and 19 pcw (E) were stained with CD36 and CD44 and/or SOX2 and SOX9 antibodies. Arrows (D) show patterning from distal to proximal regions. Yellow dashed lines (E) indicate separation of SOX9^+^ tip regions from the PDPN^+^ stalk. The *SFTPC* transcript was visualized by *in situ* hybridization chain reaction (HCR) (F) following immunostaining for CD36. (G) Flow cytometry of the human lung tip epithelial population at 10 (blue) and 20 pcw (red). (H) qRT-PCR of the freshly purified lung epithelial cells sorted from the late-stage lungs. Data normalized to fresh EPCAM^+^ cells from 20 pcw distal tissues; mean ± SD, n = 7 (15–21 pcw). Significance evaluated by one-way ANOVA with Tukey multiple comparison post-test; ns: not significant, *p < 0.05, **p < 0.01, *** p < 0.001. (I) Proportion of the freshly purified tip epithelium as CD44^+^CD36^−^ or CD44^+^CD36^+^ at 11, 13, 15, 18, 20, and 21 pcw; n = 1 each time. (J) Relative mRNA levels of the tip progenitor markers, *SOX9* and *SOX2*, and type 2 alveolar lineage markers, *SFTPC, CD36*, and *LAMP3*, in CD44^+^CD36^−^ tip epithelial population at 11 and 13 pcw, and in CD44^+^CD36^+^ tip epithelial population at 15, 16, 18, 20, and 21 pcw, by qRT-PCR. Data were normalized to fresh EPCAM^+^ cells from 20 pcw tip tissues; n = 1 at each stage. (K and L) Human fetal lung tissues during the transition from 13 to 15 pcw were stained using antibodies against CD36, CD44, and CD31 (K), or for *SFTPC* and *SOX9* mRNA (L). Three 13 pcw, two 14 pcw, and two 15 pcw samples. (M) Signal intensity of *SOX9* and *SFTPC* transcripts in Figure 1L. Ten tip regions analyzed per stage, and the intensity represented as mean ± SD. Significance evaluated by one-way ANOVA with Tukey multiple comparison post-test; ns: not significant, *p < 0.05, **p < 0.01, ***p < 0.001, ****p < 0.001. DAPI, nuclei. Scale bars, 50 μm. See also Figure S1.

We investigated distal regions of human fetal lungs by single-cell RNA sequencing (scRNA-seq)^7^ (Figure 1A). 11 epithelial cell clusters were identified, including tip, stalk, airway progenitors, AT2, and AT1 cells, each with distinct markers (Figures 1A, S1A, and S1B). Force-directed embedding showed that the clusters were largely grouped into two distinct regions by stage: 5–11 pcw (“early-mid”), and 15–22 pcw (“late”) (Figure 1A). Along the distal to proximal axis of the plot, the early- and mid-stage tips were transcriptionally connected to stalk cells, followed by airway progenitors. Whereas late-stage tips were closely linked to stalk and airway progenitors and to AT2 and AT1 cells (Figure 1A), suggesting late-stage tip cells gain alveolar differentiation competence. Differential gene expression analysis showed that late-stage tip cells co-expressed alveolar lineage markers, such as *SFTPC, LAMP3*, and *SLC34A2*, with tip progenitor markers (Figures 1B, S1A, and S1B). Analysis of tissue sections confirmed that 10–12 pcw distal tips were columnar and marked by SOX9, SOX2, and *TPPP3*. 17–22 pcw distal tip epithelium contained more cuboidal SOX2^−^, SOX9^+^, *TPPP3*^+^ cells, which co-expressed AT2 cell markers, SFTPC, and HTII-280 (Figures S1C–S1E). Fetal AT2 cells were located outside of the tip region and did not co-express progenitor markers (Figure S1E). We identified surface markers to distinguish between early-mid (airway)- and late (alveolar)-stage tips (Figures 1B and S1B). CD44 marks tip epithelial cells across all stages of lung development tested. Whereas CD36 is specific to the ~15–22 pcw stage tips where it is co-expressed with CD44, *SFTPC* and SOX9. A lower level of both CD44 and CD36 extends into the SOX9^−^/PDPN^+^ tip-adjacent (stalk) cells, and CD44 extends further proximally into the differentiating stalk (Figures 1C–1F, S1F, and S1G).

Distal lung regions were dissected to enrich for the tip, and EPCAM^+^ cells were sorted for CD44 and/or CD36. At 10 pcw, 75% of sorted cells were CD44^+^, and CD44^+^CD36^+^ cells were rare (Figures 1G and S1H). By contrast, at 20 pcw 53% of sorted cells were CD44^+^CD36^+^ and only 17% were single CD44^+^ (Figures 1G and S1H). qRT-PCR showed that the 17–20 pcw CD44^+^CD36^+^ cells robustly expressed *CD36, CD44, SFTPC*, and *SOX9*, but extremely low levels of the airway markers, *TP63* and *SOX2*, consistent with immunostaining (Figure 1H). In contrast, single CD44^+^ cells showed a higher level of *SOX2*, but much lower levels of *SFTPC* and *SOX9*, suggesting they are derived from the CD44^+^SOX9^−^ stalk region (Figures 1H and S1G). Finally, the CD44^−^CD36^−^ cells had higher levels of TP63 and *SOX2*, but low *SFTPC* and *SOX9*, indicating that they are derived from more proximal airway-lineage cells (Figure 1H). Therefore, dual expression of CD44 and CD36 marks the tip epithelial population in the 15–21 pcw stage lung, and this population co-expresses tip progenitor and alveolar markers.

### Gradual acquisition of tip alveolar lineage signature during human lung development

Flow cytometric analysis showed that the expression of CD36 was robustly acquired between 13 and 15 pcw, prior to morphological alveolar differentiation (Figure 1I). Similarly, in the CD36^+^ cells, *SFTPC, CD36*, and *LAMP3* mRNA began to increase from 13 pcw (Figure 1J). We confirmed that CD36 was detectable in the tip epithelium at 14 pcw, moreover the intensity of *SFTPC* transcripts increased while *SOX9* gradually lowered during this transition period (Figures 1K–1M). Showing that the acquisition of AT2 lineage signatures occurs gradually in the tip, prior to the differentiation of the alveolar epithelium.

### Organoids derived from late-stage distal tip epithelium exhibit alveolar lineage signatures

**Figure 2:**
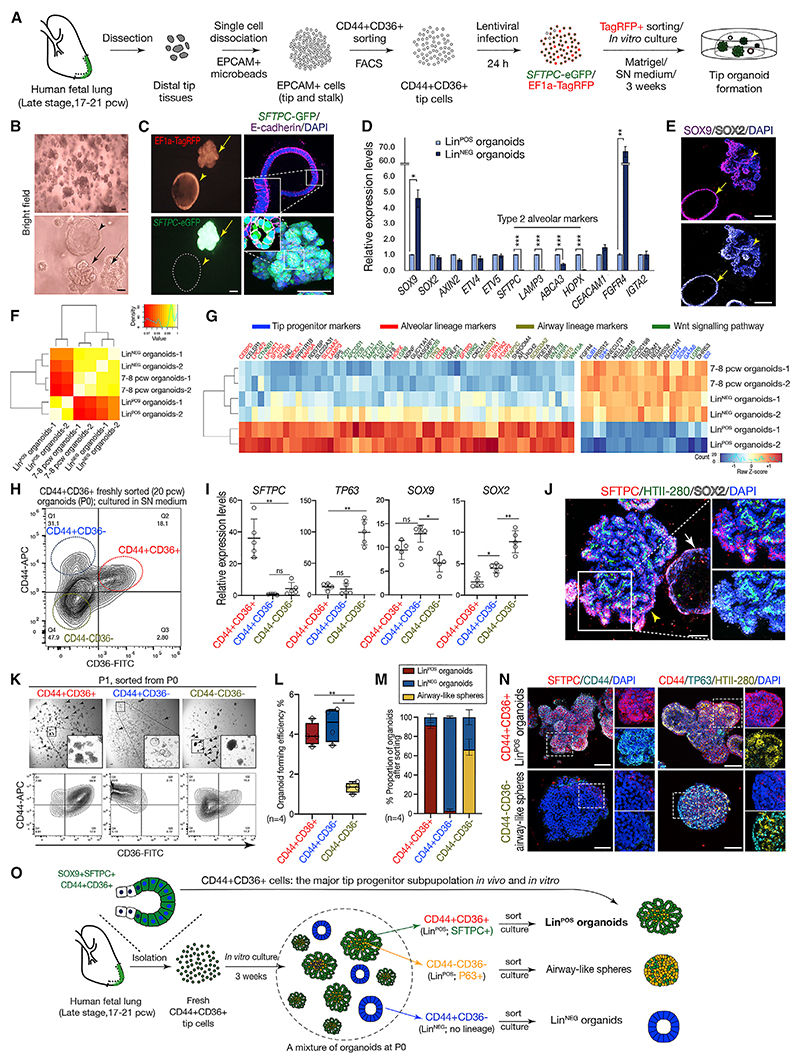
CD36, CD44 dual-positive tip cells self-renew and undergo lineage commitment *in vitro* to form late-stage lung organoids (A) Isolation and viral infection of CD44^+^CD36^+^ tip epithelial cells from human fetal lungs at 17–21 pcw, late stage, and *in vitro* culture in self-renewing (SN) medium. (B and C) Gross morphology (B) of the cultured epithelial tip organoids. Detailed morphology (C) E-cadherin (magenta); *SFTPC*-GFP and *TagRFP*. Arrows and arrowhead indicate folded Lin^POS^ organoids and cystic Lin^NEG^ organoids, respectively. Scale bars, 100 μm. (D) Gene expression profile of the Lin^POS^ and Lin^NEG^ organoids. Data are quantified by qRT-PCR; mean ± SD of 4 biological replicates. Significance evaluated by unpaired Student’s t test; *p < 0.05, **p < 0.01, ***p < 0.001. (E) Immunofluorescence analysis of the Lin^POS^ (arrowheads) and Lin^NEG^ organoids (arrow) at passage 1 cultured in the SN medium, showing co-expression of SOX9 and SOX2. DAPI, nuclei. Scale bars, 50 μm. (F) Hierarchical clustering analysis of bulk-RNA-seq data using 7–8 pcw, Lin^POS^ and Lin^NEG^ organoids. (H) Heatmap analysis of selected genes highly enriched in the 7–8 pcw, Lin^NEG^ and Lin^POS^ organoids. (H and I) Late-stage lung tip organoids sorted into 3 populations at passage zero by FACS using antibodies against CD36 and CD44 (H). The sorted P0 cell populations were analyzed by qRT-PCR (I). Data were normalized to total EPCAM^+^ cells freshly sorted from 20 pcw tissues; mean ± SD (n = 5). Significance was evaluated by one-way ANOVA with Tukey multiple comparison post-test; *p < 0.05, **p < 0.01. (J) Late-stage tip organoids at passage zero cultured in self-renewal medium stained with SFTPC, HTII-280 and SOX2 antibodies. Arrowheads indicate the tip-like, SFTPC^+^ subpopulation in the Lin^POS^ organoids. Arrows indicate the Lin^NEG^ organoids. (K–N) Passage 1 organoids were grown from the sorted CD44^+^CD36^+^, CD44^+^CD36^−^, or CD44^−^CD36^−^ populations at passage 0 and reanalyzed for CD44 and CD36 at the end of passage 1 (K; Figure 2O). Arrows and arrowheads indicate the Lin^POS^ and Lin^NEG^ organoids. The organoid forming efficiency (L), the proportion (M), and the fluorescence images (N) of the organoids of each morphological sub-type at passage 1 was measured at 3 weeks after plating. Data were represented as mean ± SD of 4 biological replicates. (O) Diagram summarizing the organoid experiments performed. CD44^+^CD36^+^ cells from late-stage lung tissues are the major tip progenitor subpopulation *in vitro*, growing into self-renewing Lin^POS^ organoids showing key features of the late-stage lung tip cells. Scale bars, 100 μm. See also Figure S2 and Table S1.

To determine the fate potential of the 17–21 pcw distal tip, CD44^+^CD36^+^ cells were cultured for 3 weeks (Figure 2A) in our established self-renewing (SN) medium.^2^ Two morphologically distinct organoids formed: cystic and folded (Figure 2B). Folded organoids consisted of cuboidal cells and expressed both progenitor and AT2 markers, including an *SFTPC*-eGFP reporter (Figure 2C). By contrast, cystic organoids had columnar cell shape and expressed tip progenitor, but not AT2, markers resembling the airway branching stage (early/mid) tips (Figures 2B–2E, S2A, and S2B). We refer to the folded and cystic organoids isolated from 17 to 21 pcw lungs as lineage positive (Lin^POS^) and negative (Lin^NEG^).

We performed RNA-seq to compare the transcriptome of passaged early-mid stage tip organoids derived from 7 to 8 pcw with passaged 17–21 pcw Lin^NEG^ and Lin^POS^ organoids (Figure S2C). Hierarchical clustering and principal component analysis showed that the Lin^NEG^ organoids were very similar to the 7–8 pcw (early-mid tip) organoids, but distinct from the Lin^POS^ organoids (Figures 2F and S2D). We identified >280 differentially expressed genes between the Lin^POS^ and Lin^NEG^ organoids (Table S1; Figure S2E; log_2_FC > 4, p < 0.05). Similar to the 7–8 pcw organoids, the Lin^NEG^ organoids were highly associated with gene ontology (GO) terms related to ion transport and branching morphogenesis, confirming that they resemble the early-mid distal tips. Whereas the Lin^POS^ organoids had significant GO terms for respiratory gaseous exchange and lung alveolus development, as well as canonical Wnt pathway signaling (Figures S2F–S2I). Moreover, Lin^POS^ organoids were enriched for AT2 markers, Wnt signaling-related genes and low levels of airway genes (Figure 2G). These data confirm that the passaged Lin^POS^ organoids recapitulate key molecular characteristics of the alveolar/late-stage lung tip progenitors.

### Late-tip epithelial cells function as multipotent stem cells *in vitro*

We tested whether any organoids from the 17 to 21 pcw mixed Lin^POS^ and Lin^NEG^ population retained CD44 and CD36 expression after 3 weeks culture (Figure 2H). The CD44^+^CD36^+^ cells showed the highest level of *SFTPC* with a moderate level of *SOX9*, but very low levels of *SOX2* and *TP63*. They were located at the tips of the Lin^POS^ organoids where they expressed SFTPC, HTII-280, SOX9, PORCN, CD44, and KI67 (Figures 2I, 2J, and S2J–S2N). The CD44^−^CD36^−^ cells had the highest levels of airway markers *TP63* and *SOX2*. They corresponded to the inner parts of the Lin^POS^ organoids where scattered TP63^+^ cells were found (Figures 2I, 2J, and S2K). By contrast, the CD44^+^CD36^−^ cells had the highest level of *SOX9* and a moderate level of *SOX2*, but no lineage markers, and corresponded to the Lin^NEG^ organoids which had uniform CD44, SOX2, and SOX9 (Figures 2I, 2J, and S2M). These data suggested that the CD44^+^CD36^+^ late-stage tip cells originally plated had self-renewed (at the tips) and differentiated toward airway lineages (in the center) to form the Lin^POS^ organoids.

We tested whether the CD44^+^CD36^+^ cells continued to selfrenew upon passaging. P0 organoids (mixed population of Lin^POS^ and Lin^NEG^) were sorted as CD44^+^CD36^+^, CD44^+^CD36^−^ and CD44^−^CD36^−^ and cultured separately in the SN medium. Only the CD44^+^CD36^+^ cells were able to generate a large proportion of Lin^POS^ organoids with folded structure and progenitor/AT2 gene signature (Figures 2K–2O and S2O). In contrast, the CD44^+^CD36^−^ cells, derived from Lin^NEG^ organoids, produced Lin^NEG^ organoids. The CD44^−^CD36^−^ cells, derived from the center of the Lin^POS^ organoids, largely formed airway-fated spheres expressing a significantly higher level of *TP63*/TP63 (Figures 2K–2O and S2O). We confirmed that the CD44^+^CD36^+^-cell-derived Lin^POS^ organoids self-renew and maintain their cellular organization for multiple passages (Figures S2P and S2Q). These data demonstrate that the CD44^+^CD36^+^ cells are the major progenitor subpopulation *in vitro* and can maintain Lin^POS^ organoids. We have therefore captured the alveolar stage lung tip epithelial population that co-expresses SOX9 and AT2 markers in the Lin^POS^ organoids.

### Cultured 17–21 pcw tip cells differentiate readily into alveolar-type-2 cells

**Figure 3:**
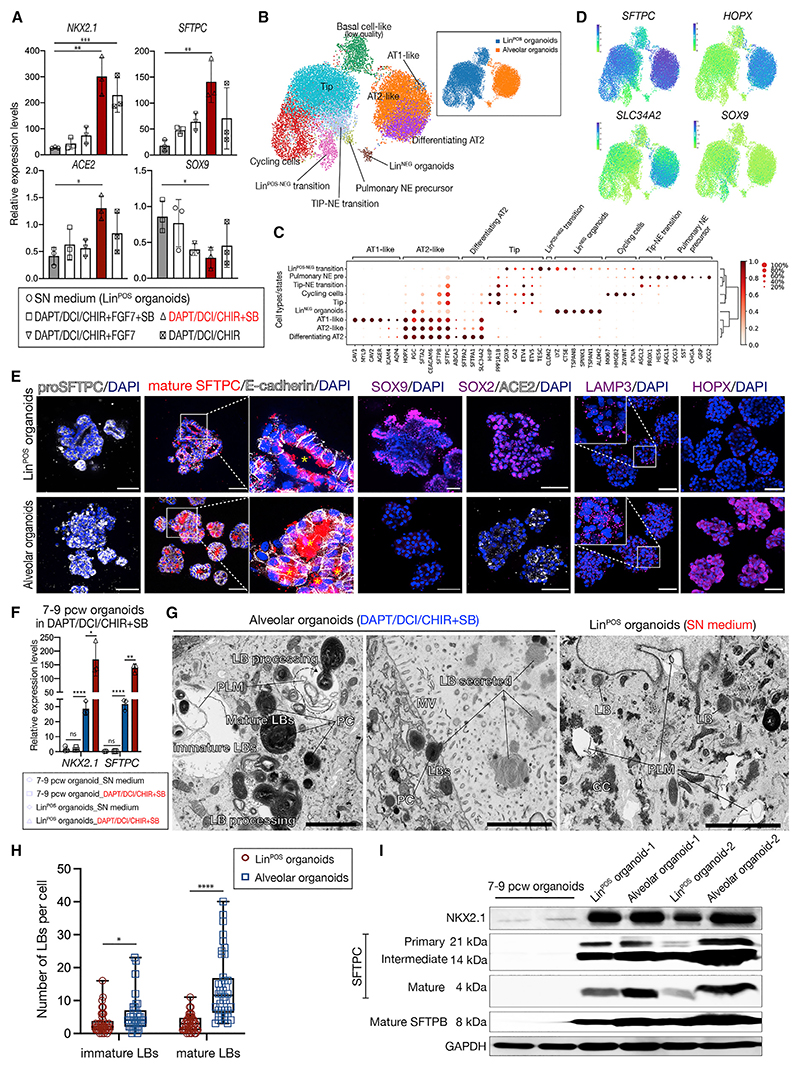
Efficient *in vitro* differentiation of Lin^POS^ organoids to alveolar cell fate (A) qRT-PCR of the Lin^POS^ organoids cultured in SN medium or alveolar-induction culture conditions containing combinations of DAPT, dexamethasone/cyclic AMP/IBMX (DCI), CHIR with/without SB431542 (SB) and FGF7, for 1 week. *NKX2.1, SFTPC*, and *SOX9* levels were normalized to EPCAM^+^ cells, and *ACE2* was normalized to EPCAM^−^ cells, freshly sorted from 20 pcw tip tissues; mean ± SD of four biological replicates. Significance was evaluated by one-way ANOVA with Dunnett multiple comparison post-test; *p < 0.05, **p < 0.01, ***p < 0.001. (B) UMAP embedding of single-cell RNA sequencing profile from Lin^POS^ and alveolar organoids colored by cell types/states, showing heterogeneous nature of the organoids. Inbox describes the origin of the cell sources in the UMAP. (C) Dot plot describing differential marker gene expression level and cell proportion within a cluster, by cell types/states. (D) UMAP plots showing transcript expression of AT2 lineage markers, *SFTPC, HOPX, SLC34A2*, and tip progenitor marker, *SOX9*, in the organoids. (E) Immunofluorescent analysis of Lin^POS^ and alveolar organoids. Asterisk (*) indicates a lumen. DAPI, nuclei. Scale bars, 50 μm. (F) qRT-PCR of *NKX2.1* and *SFTPC* in 7–9 pcw tip and Lin^POS^ organoids cultured in the SN medium or in DAPT/DCI/CHIR plus SB. Data were normalized to EPCAM^+^ cells freshly isolated from 20 pcw tip tissues; mean ± SD of three biological replicates. Significance was evaluated by one-way ANOVA with Tukey multiple comparison post-test; ****p < 0.0001. (G) Electron microscopy images of the alveolar organoids (left) and the Lin^POS^ organoids (right). LBs, lamellar bodies; PC, projection core; MV, microvilli; GC, glycogen; and PLM, primitive lipid membrane within a pool of monoparticulate glycogen at an early stage in the formation of LBs. Scale bars, 3 μm. (H) Numbers of LBs per cells in the Lin^POS^ organoids (red) and alveolar organoids (blue). Immature and mature LBs were measured in total 40 cells from two biological samples for each condition. Significance evaluated by unpaired Student’s t test; *p < 0.05, **p < 0.01, ***p < 0.001, ****p < 0.0001. (I) Western blot showing NKX2.1 level and SFTPB/SFTPC processing in the 7–9 pcw, Lin^POS^, and alveolar organoids. GAPDH (glyceraldehyde-3-phosphate dehydrogenase) was used for a loading control. See also Figure S3.

Our scRNA-seq data (Figures 1A and 1B) suggested that the late-stage tip cells gain alveolar differentiation competence. We therefore tested whether the Lin^POS^ organoids could differentiate into AT2 cells. In medium containing DAPT (γ-secretase inhibitor; Notch inhibitior), DCI (dexamethasone, cyclic AMP [cAMP], and 3-Isobutyl-1-methylxanthine [IBMX]), CHIR (CHIR99021; Wnt agonist) and SB431542 (TGF-β inhibition), *NKX2-1, SFTPC*, and *ACE2* were upregulated and *SOX9, SOX2*, and *TP63* downregulated (Figures 3A and S3A–S3C).

Similarly, the *SFTPC*-GFP reporter and the AT2-specific LAMP3, HOPX, and ACE2 proteins increased^11^ (Figures 3A, 3E, S3B, and S3C). Withdrawal of DAPT, DCI, or SB431542 caused reduction of mature SFTPC and AT2 gene expression (Figures S3D–S3F). scRNA-seq showed that Lin^POS^ organoids mostly comprised tip and cycling tip cells with small subpopulations of pulmonary neuroendocrine (NE) precursors, basal-like cells, and transitional cell states (Figures 3B–3D). Whereas, differentiated organoids comprised AT2-like cells, with a small number of AT1-like cells (Figures 3C, 3D, and S3G). The medium condition efficiently differentiates the Lin^POS^ organoids to alveolar lineages.

We directly compared the ability of 7–9 pcw, and 17–21 pcw Lin^NEG^ and Lin^POS^ organoids to differentiate to alveolar fates (Figure 3F). This confirmed that 17–21 pcw tips (Lin^POS^ organoids) are in a distinct differentiation-ready state, confirming that cell-intrinsic changes occur in tip progenitors during development.

Electron microscopy revealed that the Lin^POS^ organoids in the SN medium contained rare, immature lamellar bodies (LBs) usually surrounded by glycogen (GC) (Figures 3G and 3H). Higher numbers of LBs with a characteristic surfactant projection core (PC)^12,13^ were produced and secreted in the differentiated organoids (Figures 3E, 3G, and 3H). NKX2.1 protein, pro-SFTPC and SFTPB processing (Figure 3I), and SFTPC secretion (Figure 3E)^13^ were increased following AT2 differentiation. These data demonstrate that the Lin^POS^ organoids are readily differentiated to a mature AT2 fate and suggest that NKX2.1 levels are important in this process.

### Coordinated control of the late-stage tip epithelial cell fate by Wnt and FGF signaling

**Figure 4:**
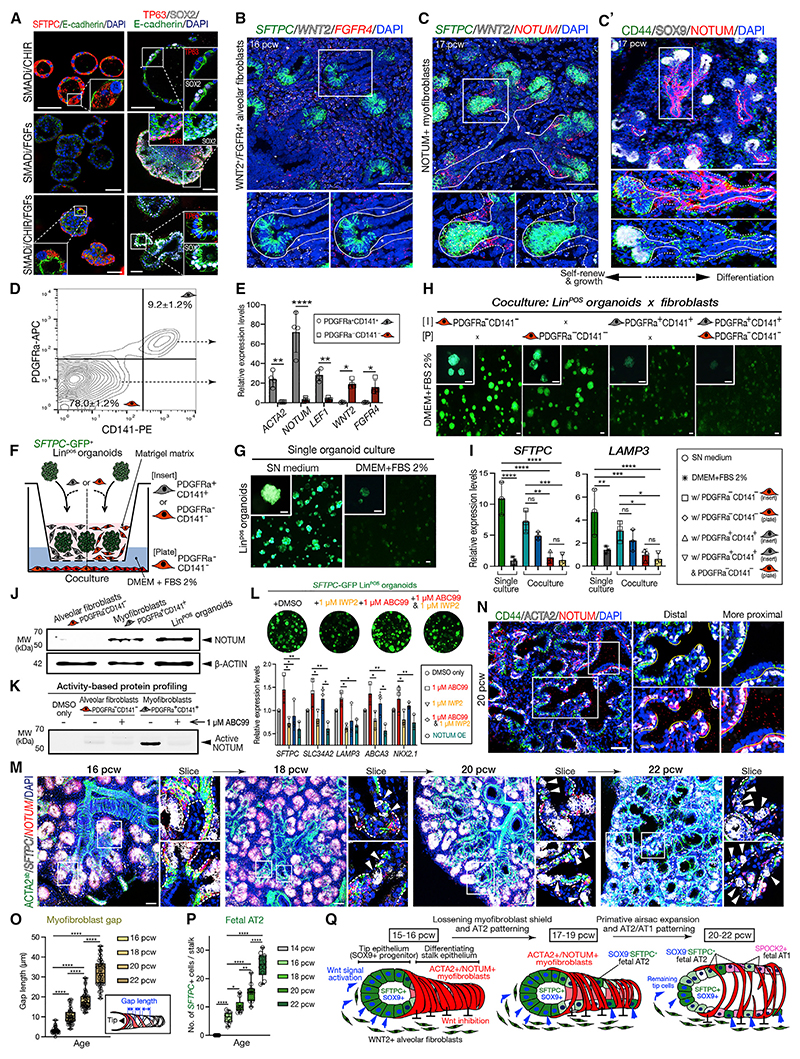
Spatial patterning of the differentiating alveolar epithelium by modulating Wnt signaling activity (A) Immunofluorescence analysis of the tip organoids at passage zero cultured in the different culture conditions. Antibodies against E-cadherin, SFTPC, TP63, and SOX2 were used. Scale bars, 20 μm. (B and C) Frozen sections of 15–17 pcw human fetal lung stained by *in situ* HCR and/or antibodies. (B) 16 pcw, *SFTPC, WNT2*, and *FGFR4* probes. (C) 17 pcw, *SFTPC, WNT2*, and *NOTUM* probes. (C′) 17 pcw, CD44, SOX9, and NOTUM antibodies. Asterisks (B and C) represent *NOTUM*^+^ myofibroblasts in the tissues. Lines and dashed lines indicate the boundaries of epithelial cells and myofibroblasts, respectively. Scale bars, 50 μm. (D) Isolation of PDGFRA^+^CD141^+^ myofibroblasts and PDGFRA^−^CD141^−^ alveolar fibroblasts from human fetal lung tissues at 17–21 pcw using a combination of PDGFRA-APC and CD141-PE antibodies. (E) qRT-PCR of PDGFRa^+^CD141^+^ myofibroblasts and PDGFRa^−^CD141^−^ alveolar fibroblasts freshly isolated from 17 to 21 pcw human lung tissues. Data were normalized to the total isolated fibroblast population; mean ± SD of biological 4 replicates. Significance was evaluated by unpaired Student’s t test; *p < 0.05, **p < 0.01, ***p < 0.001. (F) Diagram illustrating *in vitro* co-culture of the isolated PDGFRa^+^CD141^+^ myofibroblasts and PDGFRa^−^CD141^−^ alveolar fibroblast with Lin^POS^ tip organoids expressing *SFTPC-*GFP. (G and H) *SFTPC-*GFP signal of Lin^POS^ tip organoids. (G) Cultured alone in self-renewing (SN) or DMEM + 2% FBS medium. (H) Co-cultured with freshly isolated fibroblast subpopulations. I, insert; P, plate. Scale bars, 100 μm. (I) qRT-PCR for *SFTPC* and *LAMP3*, 2 weeks after *in vitro* culture. Mean ± SD of 3 biological replicates. Significance was evaluated by one-way ANOVA; ns: not significant, *p < 0.05, **p < 0.01, ***p < 0.001. (J) Western blot assay of NOTUM expression in PDGFRA^−^CD141^−^ alveolar fibroblasts, PDGFRA^+^CD141^+^ myofibroblasts, and Lin^POS^ organoids. β-ACTIN was used for a loading control. 52 kDa, NOTUM. (K) Activity-based protein profiling assay of the alveolar fibroblasts and the myofibroblasts for detecting an enzymatically active form of NOTUM. (L) An inhibitor of Porcupine (1-μM IWP2) or NOTUM (1-μM ABC99) was added to the Lin^POS^ organoids in the SN medium for 1 week and analyzed by qRT-PCR. The medium was replaced every 2 days. DMSO only, a positive control. NOTUM overexpression (OE), a negative control. Data were normalized to the Lin^POS^ organoids treated with DMSO only; mean ± SD of biological 3 replicates. Significance was evaluated by one-way ANOVA; *p < 0.05, **p < 0.01. (M) Time course analysis of spatial configuration of ACTA2^+^*NOTUM*^+^ myofibroblasts and *SFTPC*^+^ tip epithelium in the human fetal lung tissues at 16, 18, 20, and 22 pcw, by *in situ* HCR followed by antibody immunostaining. Red, *NOTUM*; white, *SFTPC*; green, ACTA2. Arrowheads and dashed lines in “slice” images indicate fetal AT2 cells and myofibroblasts at the stalk regions, respectively. The *SFTPC*^+^ fetal AT2 cells are located at the gap between discontinued lines of the myofibroblasts. Thickness, 50 μm. (N) Immunofluorescence analysis of 20 pcw human fetal lung using antibodies against CD44, ACTA2, and NOTUM. Yellow lines indicate ACTA2^+^NOTUM^+^ myofibroblasts. (O) Measurement of gap length between the myofibroblasts surrounding the stalk epithelial tubes. 50 measurements from 2 biological replicates at each age. Significance was evaluated by one-way ANOVA; *p < 0.05, **p < 0.01, ***p < 0.001, ****p < 0.0001. (P) Number of *SFTPC*^+^ fetal AT2 cells was counted in the developing fetal lungs at 14, 16, 18, 20, and 22 pcw. Significance was evaluated by one-way ANOVA; *p < 0.05, **p < 0.01, ***p < 0.001, ****p < 0.0001. (Q) Diagram describes the emergence of alveolar-type cells along with conformational changes of myofibroblasts. DAPI, nuclei. See also Figures S4 and S5.

To determine which factors in SN medium are most important for promoting airway versus alveolar fate of the late-stage tips, freshly isolated tips from 17 to 21 pcw lungs were directly exposed to pairwise signal combinations (Figures 4A, S4A, and S4B). The cells did not grow in the absence of SMADi (SMAD inhibitors; Noggin and SB431542) (Figures S4B and S4C). However, we observed that two distinct populations of organoids were obtained by combining SMADi with CHIR, or with FGFs (FGF7 and FGF10) (Figure S4B). Organoids grown in SMADi/CHIR had a thin epithelium and hollow lumen. They could not be passaged and expressed high levels of the AT2 marker *SFTPC*, greater than the Lin^POS^ organoids. In contrast, organoids grown in SMADi/FGF formed spheres with a small lumen, a thicker proliferative epithelium, and expressed the highest level of the basal cell marker TP63 (Figures 4A, S4B, and S4D). In both conditions *SOX9* was lower than the Lin^POS^ and Lin^NEG^ organoids (Figure S4D). These data indicate that Wnt and FGF signaling promote the lineage determination of the 17–21 pcw tip epithelium to alveolar or airway lineages; in agreement with previous data for Wnt.^6,14^ Moreover, when SMADi/CHIR/FGFs were combined (equivalent to our SN medium) organoids displayed a mixture of alveolar and airway characteristics, as in the Lin^POS^ organoids (Figure 4A).

We demonstrated that the 17–21 pcw tips are highly plastic and can switch readily between alveolar and airway differentiation by altering the medium and observing rapid organoid morphology and gene expression changes (Figures S4E–S4G). Freshly isolated 8 pcw distal tip epithelial cells did not show similar levels of differentiation when grown in the same conditions (Figure S4H). These data again indicate that the 17–21 pcw tips are intrinsically different to the airway-stage (8 pcw) tips, being both highly plastic and in a differentiation-ready state.

### NOTUM-expressing myofibro-blasts pattern the epithelial Wnt response during alveolar formation

We reasoned that Wnt and FGF signaling also control lineage determination of the late-tip epithelium *in vivo* and investigated the role of Wnt. In 17–21 pcw tissue, the *SFTPC*^+^ tips expressed higher levels of the Wnt targets *AXIN2, WIF1*, and PORCN, compared with stalk and airway epithelium (Figures S4I–S4N and S5A–S5D). Moreover, we confirmed our single-cell data showing that *WNT2* is co-expressed with *FGFR4* in alveolar fibroblasts throughout these stages^7,11^ (Figure 4B). This led us to question how Wnt-responsive *SFTPC* could be precisely restricted to the tip epithelium in the presence of widespread *WNT2*. A secreted Wnt inhibitor, *NOTUM*,^15^ is expressed in the distal tip epithelium and the myofibroblasts that surround the differentiating stalk cells (Figures 4C, 4C′, and S5B–S5E). The *NOTUM*^*+*^ myofibroblasts coexpress the Wnt targets *LEF1* and *AXIN2*, suggesting that they also respond to Wnt (Figures S5C and S5D). We hypothesized that in response to WNT2 the myofibroblasts locally secrete NOTUM, preventing the stalk epithelium from experiencing a high level of Wnt activity, allowing cells to turn off *SFTPC* and exit the tip fate. We identified surface antigens for the isolation of WNT2^+^ fibroblasts or NOTUM^+^ myofibroblasts (Figures 4E and S5F–S5H). Isolated PDGFRA^+^CD141^+^ myofibroblasts express high levels of *ACTA2, NOTUM*, and *LEF1*. Whereas PDGFRA^−^CD141^−^ fibroblasts express high levels of *WNT2* and *FGFR4* (Figures 4D and 4E). This gene expression is maintained if the cell types are cultured individually for 14 days. When freshly isolated fibroblasts and myofibroblasts were co-cultured, the levels of myofibroblast *LEF1* and *NOTUM* increased, suggesting that they are indeed responding to WNT2 from the fibroblasts (Figure S5I).

We asked whether co-culture with the PDGFRA^−^CD141^−^ fibroblasts, or PDGFRA^+^CD141^+^ myofibroblasts, could affect *SFTPC* expression in the Lin^POS^ organoids (Figure 4F). Lin^POS^ organoids robustly express *SFTPC*-GFP when cultured in SN medium, but not in 2% FBS (Figure 4G). However, co-culture with PDGFRA^−^CD141^−^ fibroblasts can substitute for SN medium and maintain *SFTPC*-GFP and endogenous *SFTPC* and *LAMP3* (Figures 4H and 4I). By contrast, co-culture of the Lin^POS^ organoids with PDGFRA^+^CD141^+^ myofibroblasts, or both PDGFRA^+^CD141^+^ myofibroblasts and PDGFRA^−^CD141^−^ fibroblasts, did not support AT2 gene expression. We propose that *in vivo* WNT2-expressing alveolar fibroblasts promote *SFTPC* expression in the distal tip. Moreover, that differentiating stalk cells are protected from the Wnt signal by the NOTUM-secreting myofibroblasts allowing them to turn off *SFTPC* and enter a differentiation program (Figure S5M).

To test the specific role of NOTUM-mediated inhibition of Wnt, we performed loss- and gain-of-function studies by treating with a NOTUM inhibitor ABC99, by overexpressing NOTUM (NOTUM-OE), or using conditioned medium (NOTUM-CM) (Figures 4J, 4K, and S5J–S5L).^16,17^ In the co-culture, treatment with 1-μM ABC99 efficiently inhibited mesenchymal and epithelial NOTUM activity, resulting in prolonged expression of *SFTPC*-GFP reporter even in the absence of exogenous Wnt agonists (Figure S5J). By contrast, the Lin^POS^ organoids co-cultured with the NOTUM-OE fibroblasts, or treated with the NOTUM-CM, lose *SFTPC*-GFP reporter expression, which is restored by ABC99 treatment (Figures S5K and S5L). This shows that Wnt activity in the Lin^POS^ organoids relies on both paracrine signaling from the fibroblasts and partly on autocrine Wnt. Indeed, treatment of 1-μM IWP2, an inhibitor of Porcupine that is essential for Wnt secretion and activation, resulted in reduction of *SFTPC*-GFP and AT2 gene transcription in the Lin^POS^ organoids, which could be restored by ABC99 treatment (Figure 4L). These data demonstrate that NOTUM activity in the distal tip epithelium and the myofibroblasts function as a highly efficient Wnt regulatory machinery that controls epithelial tip cell fate in the developing lung.

The distribution of Wnt and NOTUM-expressing cells is spatio-temporally regulated *in vivo*, consistent with an additional role in AT2 patterning. At 16 pcw the ACTA2^+^*NOTUM*^+^ myofibroblasts tightly shield the adjacent lower tip and the stalk regions, likely blocking Wnt signaling and allowing the tip cells to turn off *SFTPC* (and *SOX9*) and enter the stalk state. At 18–22 pcw, the myofibroblasts became loosely coiled and the stalk epithelium between them is now exposed to the *WNT2*^+^ alveolar fibroblasts (Figures 4M–4O and 4Q). Simultaneously, *SFTPC*^+^ differentiating AT2 cells were observed in the stalk and increased in number over time, filling the gaps between the myofibroblasts (Figures 4M, 4P, 4Q, S5N, and S5O; Videos S1, S2, and S3). At 20 pcw onward, *NKX2.1*^+^, *SPOCK2*^+^ differentiating AT1 cells emerged at the *SFTPC*^−^ stalk regions and were aligned with the myofibroblasts (Figures 4Q, S5O, and S5P). Overall, these data show dynamic, spatiotemporal changes of the myofibroblasts in the alveolar niche of the late-stage lungs and support the concept that the differentiating alveolar epithelium is patterned by the myofibroblasts (Figures 4Q and S5M).

### NKX2.1 is a major driving force for alveolar differentiation in the tip epithelial organoids

**Figure 5:**
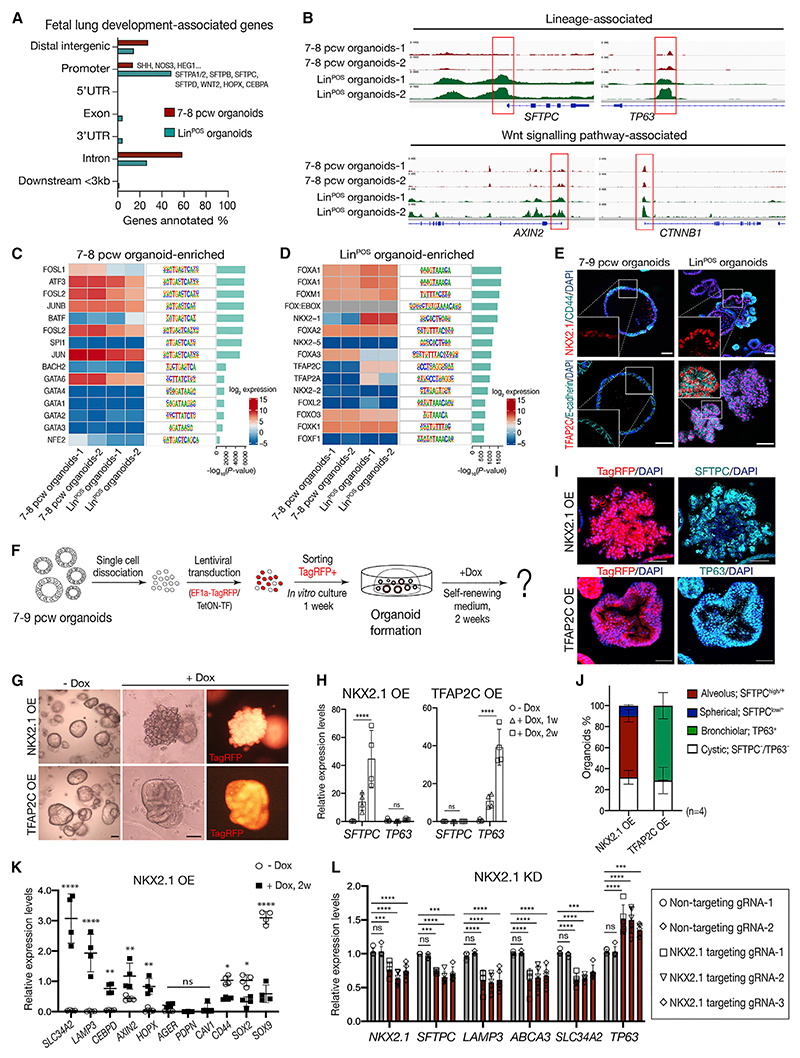
Identification of key transcription factors controlling airway and alveolar lineage differentiation using the organoid system (A) Genomic distribution of differentially accessible chromatin regions associated with human fetal lung development between 7 and 8 pcw and Lin^POS^ organoids. (B) Representative ATAC-seq tracks visualized in Integrative Genomics Viewer (IGV) at *SFTPC, TP63, AXIN2*, and *CTNNB1*. Red box indicates the promoter. (C and D) HOMER motif analysis coupled with RNA-seq data. The top 15 most highly enriched motifs and TF gene expression level (heatmap) are shown for 7–8 pcw (C) and Lin^POS^ organoids (D). (C) 7–8 pcw and Lin^POS^ organoids stained with antibodies against NKX2.1, TFAP2C, CD44, and E-cadherin. (D) Diagram showing doxycycline-inducible overexpression of NKX2.1 and/or TFAP2C in 7–9 pcw organoids. Constitutively expressed TagRFP was used for sorting transduced cells. (E) Morphology of the 7–9 pcw organoids overexpressing NKX2.1 or TFAP2C for 2 weeks. Scale bars, 100 μm. (F) Relative mRNA levels of *SFTPC* and *TP63* were measured by qRT-PCR in NKX2.1- or TFAP2C-OE 7–9 pcw organoids. Data were normalized to EPCAM^+^ cells freshly isolated from 20 pcw tip tissues; mean ± SD of four biological replicates. Significance was evaluated by one-way ANOVA with Tukey multiple comparison post-test; ns: not significant, ****p < 0001. (I and J) SFTPC and TP63 antibody staining of 7–9 pcw organoids overexpressing NKX2.1 or TFAP2C for 2 weeks (I). The proportion of the organoids positively stained in (J) was measured based on morphology and signal intensity. 4 biological replicates. (K) qRT-PCR of 7–9 pcw organoids overexpressing NKX2.1 for 2 weeks. Data were normalized to EPCAM^+^ cells freshly isolated from 20 pcw tip tissues; mean ± SD of four biological replicates. Significance was evaluated by one-way ANOVA with Tukey multiple comparison post-test; ns: not significant, *p < 0.05, **p < 0.01, ***p < 0.001, and ****p < 0.0001. (L) Knockdown (KD) of endogenous *NKX2.1* in the Lin^POS^ organoids by CRISPR-dCas9-KRAB system. Data were normalized to non-targeting gRNAs; mean ± SD of 5 biological replicates. Significance was evaluated by one-way ANOVA with Tukey multiple comparison post-test; ns: not significant, *p < 0.05, **p < 0.01, ***p < 0.001, ****p < 0.0001. DAPI indicates nuclei. Scale bars, 50 μm. See also Figure S6 and Table S2.

To identify putative TFs for cell differentiation, we analyzed chromatin accessibility of 7–8 pcw and Lin^POS^ organoids by bulk ATAC-seq (assay for transposase-accessible chromatin using sequencing). There were ~2-fold more differentially open chromatin regions in the Lin^POS^ than 7–8 pcw organoids, consistent with increased cell-type complexity of Lin^POS^ organoids (Figure S6A; Table S2). The gene feature distribution of the differentially opened chromatin was similar in both organoid types (Figure S6B). GO analysis of the genes nearest to differentially open chromatin was consistent with the RNA-seq data (Figures 2 and S6C–S6E). However, a much higher proportion of lung-development-associated genes had open chromatin at the promoter regions in the Lin^POS^ organoids (Figure 5A). For example, the promoter regions of lung differentiation genes *SFTPC, TP63*, and *CD36* and Wnt signaling genes, *AXIN2, CTNNB1, DVL3, LRRK2*, and *TCF7L1*, were more accessible in Lin^POS^ than 7–8 pcw organoids (Figures 5B, S6D, and S6E). These data strongly suggest that the chromatin accessibility of the Lin^POS^ organoids is intrinsically more favorable for lineage differentiation than the 7–8 pcw tip organoids, consistent with our functional assays.

To predict which TFs control cell fate, we performed TF motif analysis in the differential ATAC-seq peaks and compared this with our RNA-seq data to focus on TFs whose binding sites and RNA levels changed concordantly. In the 7–8 pcw organoids, the motifs for FOSL1 and GATA6 binding were predictive of function (Figures 5C and S6F). Moreover, GATA6 OE in the Lin^POS^ organoids resulted in a fate conversion of 10%–15% of the Lin^POS^ organoids to cystic Lin^NEG^ organoids (Figures S6G– S6I). In the Lin^POS^ organoids, NKX2.1 and TFAP2C motifs were accessible, and these TFs were highly transcribed (Figure 5D). Immunostaining confirmed that NKX2.1 is more strongly expressed in the Lin^POS^ than 7–8 pcw organoids. Moreover, TFAP2C was absent in 7–8 pcw organoids, but ubiquitous in the Lin^POS^ organoids (Figure 5E). *In vivo, NKX2-1* transcripts were most highly expressed in the tip and reduced in the stalk and *SCGB3A2*^+^ distal airway regions, whereas *TFAP2C* was expressed in the airway epithelium (Figure S6J–S6L).

OE of NKX2.1 and TFAP2C in undifferentiated 7–9 pcw organoids tested whether either factor was sufficient to induce differentiation to the alveolar or airway lineages (Figure 5F). NKX2.1 OE resulted in ~60% of the 7–9 pcw organoids acquiring alveolar-like structure with high levels of *SFTPC* (Figures 5G–5J and S6M). NKX2.1 also upregulated other AT2 markers including *SCL34A2, LAMP3, CEBPD, HOPX*, and *AXIN2* but downregulated tip markers *SOX9, SOX2*, and *CD44* (Figure 5K). In contrast, TFAP2C OE caused ~70% of the organoids to form bronchiolar-like structures and significantly increased basal cell markers TP63/*P63, KRT5*, and *NGFR*, (Figures 5G–5J, S6M, and S6N). NKX2.1 and TFAP2C function as key regulators of differentiation toward AT2 and basal cell lineages respectively.

The Lin^POS^ organoids co-express high levels of NKX2-1 and TFAP2C (Figure 5E) yet are comprised of distinct SOX9/SFTPC^+^ tip and TP63^Lo^ central regions (Figure 2). We analyzed the relationship between NKX2.1 and TFAP2C by simultaneous OE in the 7–9 pcw organoids. NKX2.1/TFAP2C OE organoids were highly folded, similar to NKX2-1 OE. Furthermore, TP63 was barely detectable, but SFTPC was markedly induced (Figure S6O). When Lin^POS^ organoids were cultured in SMADi/CHIR/FGF7, *NKX2.1*, and *SFTPC* were high, but *TP63* and *TFAP2C* were low. Whereas in SMADi/FGF7 (without the Wnt agonist), NKX2.1 deceased, *SFTPC* turned off and *TP63* and *TFAP2C* were robustly expressed (Figure S6P). These data demonstrate that high NKX2.1 expression, in combination with Wnt signaling, suppresses the airway lineages, explaining why TP63 expression is low in the Lin^POS^ organoids although TFAP2C is expressed (Figure S6P). Further support for the importance of NKX2.1 in promoting alveolar and inhibiting airway differentiation came from *NKX2.1* and *TFAP2C* knockdown experiments in the Lin^POS^ organoids. A small decrease in *NKX2.1* expression was sufficient to decrease AT2-specific gene expression and increase *TP63*, while *TFAP2C* KD did not affect AT2 gene transcription (Figures 5L and S6Q). These data suggest that Wnt signaling is a critical upstream regulator of *NKX2.1* levels and alveolar gene expression via a Wnt-NKX2.1-alveolar lineage transcriptional program.

### Differential binding of NKX2.1 orchestrates alveolar differentiation and functional maturation

**Figure 6:**
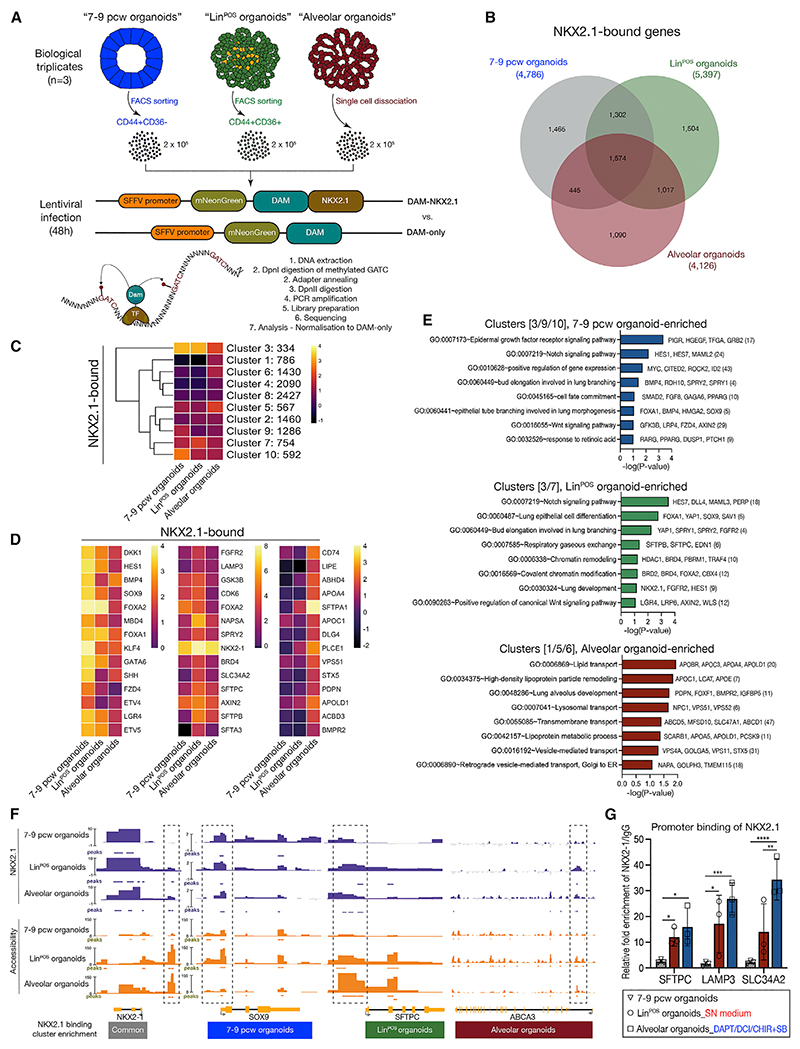
Differential binding of NKX2.1 orchestrates alveolar differentiation and functional maturation (A) Diagram describing the experimental scheme from targeted DamID. 2 × 3 10^5^ cells from three types of organoids transduced with a lentiviral vector harboring either DAM only or DAM-NKX2.1 were used for targeted DamID to analyze NKX2.1-binding pattens. (B) Venn diagram showing the number of NKX2.1-bound genes for each organoid sample. (C and D) Heatmaps illustrating k-means clustering (C) of NKX2.1-bound genes across the organoid samples and the representative genes (D) that are relatively highly enriched in each sample. Colors represent binding intensities from genes associated with peaks, which was averaged across gene bodies, including −1 kb from the TSS (transcription start site). (E) GO enrichment analysis of biological process-associated GO terms on the clusters highly enriched in the 7–9 pcw tip organoids (clusters 3/9/10), Lin^POS^ orgnaoids (clusters 3/7), and alveolar organoids (clusters 1/5/6). Representative genes associated with each GO term with the total number of genes in brackets were shown next to each graph bar. (F) Representative targeted DamID tracks of Dam-NKX2.1 (NKX2.1-bound, purple) and Dam only (chromatin accessibility, orange) at the genes commonly shared (NKX2.1) differentially enriched (SOX9, SFTPC, and ABCA3) in the organoid samples. Peaks are described below the tracks. Black dashed boxes indicate the location of promoter regions. (G) Chromatin immunoprecipitation (ChIP)-qPCR analysis for quantifying relative enrichment of NKX2.1 binding on the promoter regions of type 2 alveolar lineage markers, *SFTPC, LAMP3*, and *SLC34A2* in 7–9 pcw, Lin^POS^, and alveolar organoids. Data were normalized to the IgG control; mean ± SD of three biological replicates. See also Figure S7 and Table S4.

To investigate how NKX2.1 regulates alveolar lineage differentiation, we analyzed NKX2.1 genomic occupancy by performing targeted DamID (DNA adenine methyltransferase identification).^18–20^ We compared NKX2.1 binding in FACs isolated tip cells from 7 to 9 pcw organoids (early/mid stage tips), Lin^POS^ organoids (late-stage tips), and Lin^POS^ organoids differentiated to AT2 fate (AT2-like) (Figure 6A). Overall chromatin accessibility (assessed from background methylation in Dam-only controls) was similar in all cell types (clusters 2–4; Figure S7A). However, we noticed two clusters of genes (clusters 1/5; Figure S7A) with enrichment for GO terms related to LB and Wnt signaling (Figures S7B and S7C), which were more open in the Lin^POS^ organoids and AT2-like cells. This indicates that open chromatin for regions associated with alveolar differentiation and Wnt signaling is a feature of alveolar-fated cells, consistent with the ATAC-seq data (Figure 5).

The gene feature distribution and the numbers of NKX2.1-bound peaks were similar in all organoid types (Figures 6B and S7D; Table S4). However, *k*-means clustering revealed a dynamic distribution of NKX2.1 binding across samples (Figures 6C and 6D). All cell types shared clusters 2, 4, and 8, related to Wnt signaling, cell-cycle, and cell-cell interactions (Figure S7E). We also annotated cell-type-enriched clusters based on NKX2.1 binding intensity (Figures 6C and 6D). The 7–9 pcw and Lin^POS^ tip NKX2-1 binding clusters were enriched for genes with GO terms related to the Notch/Wnt signaling path-ways and lung bud elongation (Figures 6D–6F). However, the Lin^POS^ tips also had differential enrichment for GO terms for respiratory gaseous exchange, lung development, and chromatin remodeling, suggesting that the binding preferences of NKX2.1 shifted to alveolar lineage-associated genes to initiate differentiation (Figures 6D–6F). Strikingly, bound genes in the AT2-like cells were enriched with GO terms associated with lipid transport, lung alveolus development, lysosomal/transmembrane transport, and vesicle-mediated transport (Figures 6D–6F), indicating that NKX2.1 directly promotes surfactant protein synthesis, trafficking, and secretion. These data are consistent with the observation that the differentiated AT2-like cells showed a higher production of mature SFTPC/SFTPB and transport of mature LBs (Figure 3). NKX2.1 therefore directly activates genes related to surfactant production and secretion in alveolar-fated cells. We confirmed this for *SFTPC, LAMP3*, and *SLC34A2* by chromatin immunoprecipitation (ChIP)-qPCR (Figure 6G).

Motif enrichment analysis showed that NKX2.1-binding sites commonly shared motifs for NKX2.1 and FOXP1, regulators of lung endoderm development^21^ (Figure S7F; Table S4), and a zinc-finger protein, ZNF770. We also found putative co-TFs differentially enriched in each organoid type (Figure S7G; Table S4), suggesting additional TFs work with NKX2.1 to coordinate alveolar differentiation and maturation. The combination of chromatin accessibility changes, differential binding, and cooperation with other factors provide a framework for the dynamic activity of NKX2.1 over the course of alveolar-fate acquisition.

### Organoid assays can be used to predict the effects of human genetic variation

**Figure 7:**
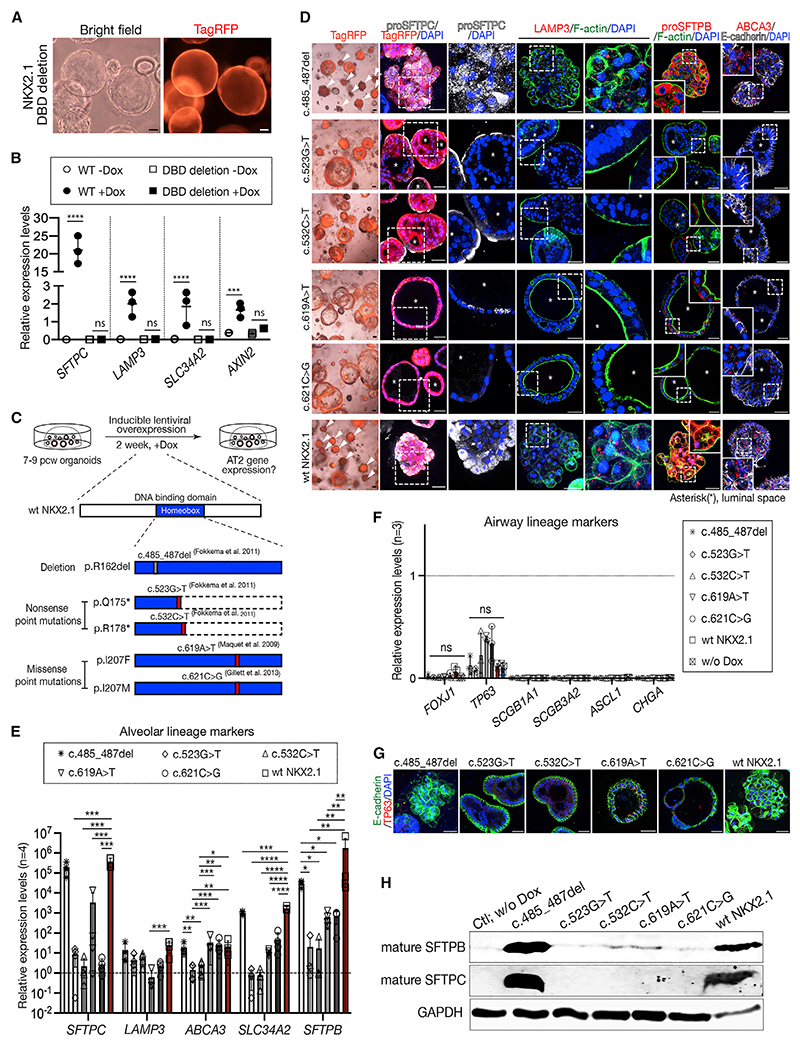
Analysis of naturally occurring human genetic variation using organoid assays (A and B) Morphology (A) and gene expression profile (B) in 7–9 pcw tip organoids overexpressing wild-type NKX2.1, or a NKX2.1 lacking a DNA-binding domain (DBD deletion), cultured for 2 weeks in the presence, or absence, of doxycycline (±DOX). (C) Diagram describing overexpression of wild-type and mutant forms of NKX2.1 in 7–9 pcw organoids using doxycycline-inducible lentiviral system. Individually, five different mutations were introduced into the DNA-binding homeobox domain; deletion of Arg^162^ (R162del),^22^ two nonsense point mutations (Q175*, R178*),^22^ and two missense point mutations (I207F, I207M)^23,24^ were tested. (D–H) Morphology and immunostaining (D and G), qRT-PCR (E and F), and western blot (H) analysis of 7–9 pcw organoids following overexpression of wild-type or mutant human NKX2.1 for 2 weeks. Data were normalized to doxycycline-non-treated lines (E) or to fresh lung-tissue-derived airway epithelial cells (F); mean ± SD of 4 (E) or 3 (F) biological replicates, respectively. Significance was evaluated by one-way ANOVA with Tukey multiple comparison post-test; *p < 0.05, **p < 0.01, ***p < 0.001. Western blot showing mature SFTPB and SFTPC. GAPDH was used for a loading control for the western blot assay. DAPI indicates nuclei. Scale bars, 50 μm.

OE of NKX2.1 lacking the homeodomain showed that DNA binding is essential for AT2 differentiation (Figures 7A and 7B). Naturally occurring human variants in the NKX2.1 homeodomain have been described.^22–24^ Many are associated with acute respiratory failure; others are predicted to be pathogenic. We hypothesized that the NKX2.1 OE assay would be a simple method to determine the effects of these variants on AT2-specific gene expression (Figure 7C). The variants differentially affected organoid morphology, cell fate specification, and surfactant protein production (Figures 7D–7G) with c.621C>G abrogating AT2 differentiation, c.619A>T causing partial AT2 differentiation, but the predicted pathogenic c.485_487 deletion behaving indistinguishably to the wild type. Expression of the NKX2.1 variants c.523G>T and c.532C>T resulted in the production of mis-localized pro-SFTPC, which was not processed to the mature form (Figures 7D and 7H), consistent with a defect in the surfactant processing and trafficking pathway.^25^ NKX2.1 therefore promotes multiple aspects of AT2 differentiation, not simply *SFTPC* transcription and, that the underlying pathology of the *NKX2.1* variants is due to inadequate surfactant production.

## Discussion

We show that the distal tip cells at 17–22 pcw of human lung development are intrinsically different to the earlier stages; retaining progenitor status yet exhibiting aspects of AT2 gene expression. Late-tip cell (Lin^POS^) organoids self-renew, capture features of late-stage human lung development, have extensive open chromatin, and can be readily differentiated to AT2-like cells. We have used this organoid system to demonstrate that Wnt signaling triggers NKX2.1-dependent human AT2 cell differentiation. Additionally, we show that antagonistic signaling interactions between differentiating fibroblasts and myofibroblasts in the developing alveolar niche provide a spatial constraint to Wnt activity, patterning the epithelium into specific lineages. Our organoid systems are readily genetically manipulated and can be used to study human genetic variation.

We demonstrate that the molecular acquisition of alveolar features precedes morphological changes occurring in the tip epithelial progenitors. Our Lin^POS^ organoids are derived from the CD44^+^,CD36^+^ 17–21 pcw tips. The CD44^+^,CD36^+^ cells are located at the tips of the organoids, maintain expression of late-tip markers, SN, and give rise to airway-fated cells in the organoid center (Figure 2). When provided with appropriate cues they differentiate to an AT2-like cell (Figure 3). *In vivo*, tip cells acquire AT2 markers gradually between 13 and 15 pcw (Figure 1). We hypothesize that during this transition period (~13–15 pcw) the tips are generating the final branch of the airway epithelium and at ~15 pcw switch their competence and generate alveolar-fated daughter cells. However, due to well-documented tip progenitor plasticity in transplantation assays,^26,27^ it is not currently possible to test this definitively.

We identify a Wnt-NKX2.1 axis as a key driver of human AT2 fate and patterning. Wnt signaling promotes AT2 differentiation of the tip epithelium *in vitro* and *in vivo* (Figures 3 and 4) while suppressing airway fate (Figure S6). This is consistent with previous reports in mouse.^28^ Similarly, human NKX2.1^+^ lung progenitors derived from PSCs expressed alveolar epithelial markers in response to Wnt.^6^ Our experiments with primary tissue provide a molecular and cellular context to the Wnt-induced alveolar patterning. Our data support a model in which opposing signals from differentiating alveolar fibroblasts (WNT2) and myofibroblasts (NOTUM) spatially restrict late-tip and AT2 identity in the late-stage human fetal lung (Figure 4). This is analogous to a recent mouse report where developing AT1 cells are aligned with, and signal to, differentiating myofibroblasts.^29^ It will be interesting to test in the future whether myofibroblast inhibition of AT2 cell fate also occurs in pulmonary fibrosis where myofibroblasts are expanded and AT2 cells lost.

We demonstrate that NKX2.1 is a key upstream TF driving the alveolar program while suppressing the airway program (Figure 5), consistent with reported roles in lung cancers.^30–32^ Our independent single-cell ATAC-seq data of the developing human lungs from 5 to 22 pcw showed that *in vivo* alveolar-fated cells, including AT1, AT2, and late-stage tip cells are highly enriched with NKX2.1 motifs, while the basal cells have TP63 motifs.^7^ The current experimental data show that differential binding of NKX2.1 facilitates alveolar differentiation and functional maturation (Figures 5 and 6), also explaining why pathogenic NKX2.1 mutations are incompatible with neonatal life (Figure 7). Some of the patient NKX2.1 mutations tested caused a partial/stalled AT2 differentiation phenotype, which is potentially rescuable.

By contrast, ectopic expression of TFAP2C promoted basal cell markers, but not other airway lineages (Figure 5). This could mean the culture conditions are permissive only for basal cell differentiation. Alternatively, TFAP2C may be specific for basal cell specification. The latter interpretation would be consistent with a report that TFAP2C acts as an upstream TF to TP63 during epidermal maturation.^33^ FGF signaling also promotes airway fate in the absence of Wnt signaling (Figure 4); consistent with our recent *in vivo* finding that airway fibroblasts form a proximal airway niche by secreting FGF7 and providing a physical barrier to block canonical Wnt ligands from reaching the airway epithelial layer.^7^ However, hPSC can be differentiated to AT2 cells in the presence of FGF7^14^ and we cannot exclude a role for FGF signaling in alveolar epithelial differentiation/proliferation.

We have identified a distinct late-tip progenitor cell in the developing human lung. Culture of these cells as organoids has allowed us to define the cellular and molecular roles of Wnt signaling and NKX2.1 in human alveolar cell development. Moreover, this differentiating organoid system will be useful for understanding the next phases of alveolar maturation in the developing human lung.

### Limitations of the study

We have developed organoid techniques for studying alveolar development in human fetal lungs. Isolation of specific fetal lung cells for organoid cultures can be performed using flow cytometry (Figures 2 and 4). Flow cytometry is inherently limited by differential epitope sensitivity to the enzymes used to generate single-cell suspensions. We controlled this using qRT-PCR to check the identity of our sorted cell populations, but we cannot exclude the possibility of some contamination from other cell lineages.

The organoid assay provided a convenient tool for testing predicted human pathogenic variants of *NKX2.1* (Figure 7). These naturally occurring mutations are observed as heterozygotes and predicted to function as dominant-negative, or neomorphic, proteins. Not all the predicted pathogenetic *NKX2.1* mutations resulted in phenotypes, and we are unable to distinguish whether these variants truly behave as wild type, or whether this is a limitation of the assay.

## Star+Methods

### Key Resources Table

**Table:** 

REAGENT or RESOURCE	SOURCE	IDENTIFIER
Antibodies		
Mouse monoclonal anti-ACTA2	Thermo Fisher Scientific	Cat#MA1-06110, RRID: AB_557419
Mouse monoclonal anti-THBD (CD141), PE conjugated	BioLegend	Cat#344104, RRID: AB_2255842
Rabbit monoclonal anti-PDGFRA	Cell Signaling Technology	Cat#3174, RRID: AB_2162345
Rabbit monoclonal anti-PDGFRA, APC conjugated	BioLegend	Cat# 313511, RRID:AB_493208
Rat monoclonal anti-CD44, APC conjugated	BioLegend	Cat# 103012, RRID:AB_312963
Rat monoclonal anti-CD44	Thermo Fisher Scientific	Cat# 17-0441-82, RRID:AB_469390
Rabbit polyclonal anti-SOX9	Merck	Cat#AB5535, RRID:AB_2239761
Sheep polyclonal anti-PDPN	R&D systems	Cat#AF3670, RRID:AB_2162070
Rat monoclonal anti-E-cadherin	Thermo Fisher Scientific	Cat#13-1900, RRID:AB_2533005
Mouse monoclonal anti-CD45, PE-Cyanine7 conjugated	Thermo Fisher Scientific	Cat#25-9459-42, RRID:AB_2573544
Mouse monoclonal anti-CD31, PE-Cyanine7 conjugated	Thermo Fisher Scientific	Cat#25-0319-42, RRID:AB_10854425
Mouse monoclonal anti-CD31	Abcam	Cat# ab9498, RRID:AB_307284
Mouse monoclonal anti-CD326, PE conjugated	BioLegend	Cat#324206, RRID:AB_756080
Mouse monoclonal anti-CD326, FITC conjugated	BioLegend	Cat# 324204, RRID:AB_756078
Mouse monoclonal anti-CD36, FITC conjugated	Thermo Fisher Scientific	Cat# 11-0369-42, RRID:AB_10718972
Rabbit polyclonal anti-CD36	Proteintech	Cat# 18836-1-AP, RRID:AB_10597244
Mouse monoclonal anti-CD9, PE-Cyanine7conjugated	BioLegend	Cat# 312115, RRID:AB_2728255
Rabbit polyclonal anti-Prosurfactant protein C	Millipore	Cat# AB3786, RRID:AB_91588
Rabbit polyclonal anti-NKX2.1 (TTF1)	Millipore	Cat# 07-601, RRID:AB_310743
Rabbit monoclonal anti-AP2 gamma/ TFAP2C	Abcam	Cat# ab218107, RRID:AB_2891087
Mouse monoclonal anti-alpha-smooth muscle actin (ACTA2)	Thermo Fisher Scientific	Cat# MA1-06110, RRID:AB_557419
Rabbit monoclonal anti-ACE2	Abcam	Cat# ab108252, RRID:AB_10864415
Mouse monoclonal antii-AXIN2	R&D systems	Cat# MAB6078, RRID:AB_2044608
Rabbit monoclonal anti-P63alpha	Cell Signaling Technology	Cat# 13109, RRID:AB_2637091
Goat polyclonal anti-SOX2	R&D systems	Cat# AF2018, RRID:AB_355110
Rabbit polyclonal anti-SOX9	Millipore	Cat# AB5535, RRID:AB_2239761
Rabbit polyclonal anti-LAMP3	Atlas Antibodies	Cat# HPA051467, RRID:AB_2681495
Mouse Monoclonal anti-HT2-280	Terrace biotech	Cat# TB-27AHT2-280, RRID:AB_2832931
Rabbit polyclonal anti-matureSFTPC	Seven Hills Bioreagents	Cat# WMAB-76694, RRID:N/A
Rabbit polyclonal anti-proSFTPC	Millipore	Cat# AB3786, RRID:AB_91588
Rabbit polyclonal anti-matureSFTPB	Seven Hills Bioreagents	Cat# WMAB-48604, RRID:N/A
Rabbit polyclonal anti-proSFTPB	Seven Hills Bioreagents	Cat# WMAB-55522, RRID:N/A
Mouse monoclonal anti-ABCA3	Seven Hills Bioreagents	Cat# WMAB-17G524, RRID:N/A
Rabbit polyclonal anti-ZO-1	Thermo Fisher Scientific	Cat# 40-2200, RRID:AB_2533456
Rabbit monoclonal anti-GATA6	Cell Signaling Technology	Cat# 5851, RRID:AB_10705521
Rabbit polyclonal anti-NOTUM	Novus Biological	Cat# NBP2-94699, RRID:N/A
Mouse monoclonal anti-KI67	BD Biosciences	Cat# 550609, RRID:AB_393778
Mouse monoclonal anti-GAPDH	Abcam	Cat# ab8245, RRID:AB_2107448
Normal Rabbit IgG	Cell Signaling Technology	Cat# 2729, RRID:AB_1031062
Biological samples		
Organoid lines: HDBR 13393, 13567, 14387,14404,14459,14556,14598, 14630, 14643, 14644, 14906, 14996, 14998	HDBR London and Newcastle	N/A
Organoid lines: BRC 1915, 1938, 1943	Brain Repair Center, University of Cambridge	N/A
Chemicals, peptides, and recombinant proteins		
ActinGreen™ 488 ReadyProbes™ Reagent	Thermo Fisher Scientific	Cat# R37110
2,2’-Thiodiethanol (TDE)	Merck	Cat# 166782
ABC99	Cambridge Bioscience	Cat# CAY25858
ActivX™ TAMRA-FP Serine Hydrolase Probe	Thermo Fisher Scientific	Cat# 88318
M-PER™ Mammalian Protein Extraction Reagent	Thermo Fisher Scientific	Cat# 78503
Halt™ Phosphatase Inhibitor Cocktail	Thermo Fisher Scientific	Cat# 78420
N2 supplement	Thermo Fisher Scientific	Cat#17502001
B27 supplement	Thermo Fisher Scientific	Cat#12587001
N-acetylcysteine	Merck	Cat#A9165
EGF	PeproTech	Cat#AF-100-15
FGF10	PeproTech	Cat#100-26
FGF7	PeproTech	Cat#100-19
Noggin	PeproTech	Cat#120-10C
R-spondin	Stem Cell Institute, University of Cambridge	N/A
CHIR99021	Stem Cell Institute, University of Cambridge	N/A
SB431542	Bio-techne	Cat# 1614
A83-01	Tocris	Cat# 2939
BMP4	Peprotech	Cat# 120-05
TGF-β1	Peprotech	Cat# 100-21
8-Bromoadenosine 3’5’-cyclic monophosphate (cAMP)	Merck	Cat# B5386
3-Isobutyl-1-methylxanthine (IBMX)	Merck	Cat# I5879
Y-27632	Merck	Cat# 688000
Dexamethasone (Dex)	Merck	Cat# D4902
DAPT	Merck	Cat# D5942
Doxycycline (Dox)	Merck	Cat# D9891
Trimethoprim (TMP)	Merck	Cat# 92131
Collagenase	Merck	Cat# C9891
Dispase	Thermo Fisher Scientific	Cat# 17105041
DNase	Merck	Cat# D4527
RBC lysis buffer	BioLegend	Cat# 420301
Cell Recovery Solution	Corning	Cat# 354253
Collagen	Merck	Cat# CLS3493
Micrococcal Nuclease (MNase)	Cell Signaling Technology	Cat# 10011S
RIPA buffer	Merck	Cat# R0278
Critical commercial assays		
CD326 (EpCAM) MicroBeads, human	Miltenyi Biotec	Cat# 130-061-101
In-Fusion® HD Cloning Plus	Takara	Cat# 638909
RNeasy Mini Kit	Qiagen	Cat# 74004
PicoPure™ RNA Isolation Kit	Thermo Fisher Scientific	Cat# KIT0204
SimpleChIP® Chromatin immunoprecipitation kit	Cell Signaling Technology	Cat# 9002
Chromium Single Cell V(D)J Kits (v1)	10X	N/A
Deposited data		
Bulk RNA-seq, ATAC-seq, DamID-seq of organoids	NCBI’s GEO	GSE178529
scRNA-seq of human fetal lungs	ArrayExpress^7^	E-MTAB-11278
scRNA-seq of lung organoids	ArrayExpress	E-MTAB-11435
Oligonucleotides		
gRNA-NKX2.1_1: 5’-GTCTGACGGCGGCAGAAGAG-3’	Horlbeck et al.^34^	N/A
gRNA-NKX2.1_2: 5’-GGACCAACAGTGCGGCCCCA-3’	Horlbeck et al.^34^	N/A
gRNA-NKX2.1_2: 5’-GAAATGAGCGAGCGAGTCTG-3’	Horlbeck et al.^34^	N/A
gRNA-TFAP2C_1: 5’-GGCGGTCTTGACACTCGCGG-3’	Horlbeck et al.^34^	N/A
gRNA-TFAP2C_2: 5’-GTCGCCAGGACACACTGTTC-3’	Horlbeck et al.^34^	N/A
gRNA-TFAP2C_3: 5’-GGTCACTGGACACGCATCGG-3’	Horlbeck et al.^34^	N/A
Recombinant DNA		
pLenti-hSPC-eGFP-EF1 a-TagRFP	This paper	N/A
pLenti-tetON-KRAB-dCas9-DHFR-EF1a- TagRFP-2A-tet3G	Sun et al.^5^	Addgene: #167935, RRID:Addgene_167935
pLenti-U6-gRNA-EF1a-EGFP-CAAX	Sun et al.^5^	Addgene: #167936, RRID:Addgene_167936
pLenti-tetON-NKX2-1-EF1a-TagRFP- 2A-tet3G	Sun et al.^5^	Addgene: #167942; RRID:Addgene_167942
pLenti-tetON-NKX2-1-delDBD-EF1a- TagRFP-2A-tet3G	This paper	N/A
pLenti-tetON-NKX2-1-R162del-EF1a- TagRFP-2A-tet3G	This paper	N/A
pLenti-tetON-NKX2-1-Q175*-EF1a- TagRFP-2A-tet3G	This paper	N/A
pLenti-tetON-NKX2-1-R178*-EF1a- TagRFP-2A-tet3G	This paper	N/A
pLenti-tetON-NKX2-1-I207F-EF1a- TagRFP-2A-tet3G	This paper	N/A
pLenti-tetON-NKX2-1-I207M -EF1a- TagRFP-2A-tet3G	This paper	N/A
pLenti-SFFV-mNG-Dam-NKX2.1	This paper	N/A
pLenti-tetON-TFAP2C-EF1a-TagRFP- 2A-tet3G	This paper	N/A
pLenti-tetON-NOTUM-EF1a-TagRFP- 2A-tet3G	This paper	N/A
pLenti-tetON-GATA6-EF1a-TagRFP- 2A-tet3G	This paper	N/A
Software and algorithms		
ImageJ (version: 2.1.0)	Schneider et al.^35^	https://imagej.nih.gov/ij/; RRID:SCR_003070
GraphPad Prism software (version: 9.1.0)	GraphPad Prism	GraphPad Prism (https://graphpad.com); RRID:SCR_015807
FlowJo software (version: 10.0.0)	FlowJo	FlowJo (https://www.flowjo.com/); RRID:SCR_008520
EdgeR (version 3.16.5)	Choi et al.^36^	https://bioconductor.org/packages/release/bioc/html/edgeR.html
DAVID	da Huang et al.^37^	https://david.ncifcrf.gov/
Enrichr	Kuleshov et al.^38^	https://maayanlab.doud/Enrichr/
fgsea	Korotkevich et al.^39^	https://montilab.github.io/hypeR-docs/articles/docs/fgsea.html
MACS2 (version: 2.1.2)	Zhang et al.^40^	https://github.com/macs3-project/MACS
HOMER (version: 4.11)	Heinz et al.^41^	http://homer.ucsd.edu/homer/
Cellxgene (version: 0.16.7)	Megill et al.^42^	https://github.com/chanzuckerberg/cellxgene
GREAT	McLean et al.^43^	https://github.com/bgruening/galaxytools/issues/184
MEME	McLeayand Bailey^44^	https://meme-suite.org/meme/doc/meme-chip.html?man_type=web
IGV (version: 2.4.19)	Thorvaldsdottir et al.^45^	https://igv.org/
HOCOMOCO Human (version: 11)	Kulakovskiy et al.^46^	https://hocomoco11.autosome.org/
Bedtools (version: 2.26.0)	Quinlan and Hall^47^	https://github.com/arq5x/bedtools2/releases?after=v2.26.0
Scanpy (version: 1.8.2)	Wolf et al.^48^	https://github.com/scverse/scanpy
STARsolo (version: 2.7.3a)	Kaminow et al.^49^	https://github.com/alexdobin/STAR/blob/master/docs/STARsolo.md
EmptyDrop	Lun et al.^50^	https://github.com/MarioniLab/DropletUtils
Other		
SH800S Cell Sorter	Sony Biotechnology	N/A
BD Influx™ Cell Sorter	BD biosciences	N/A

### Resource Availability

### Lead contact

Lead contact Further information and requests for resources and reagents should be directed to and will be fulfilled by the lead contact, Emma L. Rawlins (e.rawlins@gurdon.cam.ac.uk).

### Materials availability

Lung organoid lines used in this study are available from the lead contact, Emma L. Rawlins (e.rawlins@gurdon.cam.ac.uk), with a completed Materials Transfer Agreement.

### Data and code availability

- Sequencing data have been deposited at ArrayExpress and GEO and are publicly available. Accession numbers are listed in the key resources table. Processed single cell sequencing data reported in this paper are available at https://fetal-lung.cellgeni.sanger.ac.uk/.
- This paper does not report original code.
- Any additional information required to reanalyse the data reported in this paper is available from the lead contact upon request.

## Experimental Model and Subject Details

### Human embryonic and fetal lung tissue

Human embryonic and fetal lung tissues were provided from terminations of pregnancy from Cambridge University Hospitals NHS Foundation Trust under permission from NHS Research Ethical Committee (96/085) and the MRC/Wellcome Trust Human Developmental Biology Resource (London and Newcastle, University College London (UCL) site REC reference: 18/LO/0822; Newcastle site REC reference: 18/NE/0290; Project 200454; www.hdbr.org). Sample age ranged from 4 to 23 weeks of gestation (post-conception weeks; pcw). Stages of the samples were determined according to their external physical appearance and measurements. All the samples used for the current study had no known genetic abnormalities. Sample gender was unknown at the time of collection and was not determined.

## Method Details

### *In vitro* culture of human fetal lung organoids

The isolated tip epithelial cells were embedded in Matrigel (Corning, 356231) and cultured in 48-well plates in self-renewing (SN) medium: Advanced DMEM/F12 supplemented with 1x GlutaMax, 1 mM HEPES and Penicillin/Streptomycin, 1X B27 supplement (without Vitamin A), 1X N2 supplement, 1.25 mM n-Acetylcysteine, 50 ng/ml recombinant human EGF (PeproTech, AF-100-15), 100 ng/ml recombinant human Noggin (PeproTech, 120-10C), 100 ng/ml recombinant human FGF10 (PeproTech, 100-26), 100 ng/ml recombinant human FGF7 (PeproTech, 100-19), 3 μM CHIR99021 (Stem Cell Institute, University of Cambridge) and 10 μM SB431542 (Bio-Techne, 1614). The culture medium was replaced every 2 days and the organoids were usually split 1:3 once per week by breaking them into small fragments. Numbers of replicates are indicated in figure legends. To activate dual SMAD signalling (Figure S4B),10 ng/μl recombinant human BMP4 (Peprotech, 120-05) and 10 ng/μl recombinant human TGF-β1 (Peprotech, 100-21) were added to the medium instead of SB431542 and Noggin.

To perform *in vitro* co-culture experiments, freshly sorted 2x10^5^ PDGFRA^+^CD141^+^ myofibroblasts or PDGFRA^−^CD141^−^ fibroblasts were mixed with *SFTPC*-eGFP^+^ Lin^POS^ organoids in 100 μl Matrigel and then loaded into an insert of transwell (Merck, CLS3493). On the bottom well plates, coated with Collagen (Merck, CLS3493), 4 x 10^5^ PDGFRA^−^CD141^−^ fibroblasts were plated in the culture medium containing 2% fetal bovine serum (FBS; Thermo Fisher Scientific, 10500064) in the Advanced DMEM/F12 supplemented with 1x GlutaMax, 1 mM HEPES and Penicillin/Streptomycin. 100 μl culture medium was added every 2 days. For coculture of PDGFRA^+^CD141^+^ myofibroblasts with PDGFRA^−^CD141^−^ fibroblasts, 2x10^5^ of PDGFRA^+^CD141^+^ myofibroblasts were plated on the insert and 4x10^5^ of PDGFRA^−^CD141^−^ fibroblasts were plated on the bottom well plate. After 2 weeks of co-cultures the organoids, or the mesenchymal cells, were harvested for further analysis.

### Isolation of tip epithelial cells, myofibroblasts, and alveolar fibroblasts

For isolation of CD44^+^, or CD44^+^CD36^+^, tip epithelium directly from the distal lung tissues, the tissues were finely dissected into tiny pieces and enzymatically digested into single cells by incubating in a dissociation solution containing 0.125 mg/ml Collagenase (Merck, C9891), 1 U/ml Dispase (Thermo Fisher Scientific, 17105041) and 0.1 U/μl DNase (Merck, D4527), in a rotating incubator for 1 hour at 37°C. After rinsing in washing buffer containing 2% FBS in cold PBS the cells were filtered through a 100 μm strainer and harvested by centrifugation. The cell pellets were resuspended and treated with RBC lysis buffer (BioLegend, 420301). Next, the cells were rinsed in the washing buffer and then incubated with primary antibodies against CD45 (1:100; PE-Cy7 conjugated, Thermo Fisher Scientific, 25-9459-42), CD31 (1:100; PE-Cy7 conjugated, Thermo Fisher Scientific, 25-0319-42), EPCAM (1:100; PE-conjugated; BioLegend, 324206), CD44 (1:200; APC-conjugated; BioLegend, 103012), and CD36 (1:100; FITC, conjugated; Thermo Fisher Scientific, 11-0369-42), with a viability dye, Zombie (Biolegend, 423113) for 25 min on ice. Following removal of dead cells and immune/endothelial cells, the EPCAM+ epithelial cells were sorted by CD44 and/or CD36 expression by FACS (BD Influx™ Cell Sorter) (Figure 1G).

Alternatively, the tip epithelial cells were isolated by EPCAM^+^ magnetic-activated cell sorting (MACS) beads according to the manufacturer’s instruction (CD326 MicroBeads, human, Miltenyi Biotec) from the distal lung tissues. Then, the enriched EPCAM^+^ epithelial cells were sorted by CD44 and/or CD36 expression by FACS (SH800S Cell Sorter) to more purely enrich the tip cell population (Figure 2A).

To purify myofibroblasts and alveolar fibroblasts, the single cells dissociated from the distal lung tissues were incubated with the following primary antibodies: CD45 (1:100; PE-Cy7 conjugated, Thermo Fisher Scientific, 25-9459-42), CD31 (1:100; PE-Cy7 conjugated, Thermo Fisher Scientific, 25-0319-42), CD9 (1:100; PE-Cy7 conjugated, BioLegend, 312115), EPCAM (1:100; FITC-conjugated, BioLegend, 324204), PDGFRA (1:100; APC-conjugated, BioLegend, 313511), CD141 (1:100; PE-conjugated, BioLegend, 344104), with the viability dye, Zombie (Biolegend, 423113). After removing dead cells, immune/endothelial cells, airway smooth muscle cells, and epithelial cells, the cells are sorted by PDGFRA and/or CD141 expression using BD Influx Cell Sorter. The sorted cells were directly applied to an organoid coculture or a gene expression analysis.

### Alveolar differentiation of Lin^POS^ organoids

The Lin^POS^ organoids were embedded in Matrigel and cultured for 7 days in alveolar type 2 (AT2) differentiation medium: Advanced DMEM/F12 supplemented with 1x GlutaMax, 1 mM HEPES and Penicillin/Streptomycin, 1X B27 supplement (without Vitamin A), 1x N2 supplement, 1.25 mM n-Acetylcysteine, 10 mM CHIR99021, 50 μM Dexamethasone (Merck, D4902), 0.1 M 8-Bromoadenosine 3’5’-cyclic monophosphate (cAMP; Merck, B5386), 0.1 M 3-Isobutyl-1-methylxanthine (IBMX; Merck, 15679), 50 mM DAPT (Merck, D5942) with 10 mM SB431542 or 10 mM A83-01 (Tocris, 2939). The culture medium was replaced every 2 days without passaging.

### Lentiviral transduction

To introduce a reporter system into the tip epithelial cells, the lentiviral vector pHAGE hSPC-eGFP-W given from Darrell Kotton (Addgene plasmid # 36450; http://n2t.net/addgene:36450; RRID: Addgene_36450) was modified by inserting EF1a-promoter TagRFP cassette. The tip epithelial cells were infected with the modified lentiviral vector for 24 hours at 37°C in a single cell suspension in the SN medium containing 10 μM Y-27632 (Merck, 688000). After 24 hours, the cells were embedded to the Matrigel and cultured in the SN medium containing 10 μM Y-27632 for another 48 hours to support single cell survival. The cultured cells were further sorted by eGFP/TagRFP signals to enrich the infected cells.

For overexpressing NKX2.1 and/or TFAP2C, Tet-ON 3G doxycycline (Dox)-inducible lentiviral vector (Takara, 631337) was modified by inserting EF1a-TagRFP-2A-tet3G with tetON-NKX2-1 CDS, or by inserting EF1a-mNeonGreen-2A-tet3G with tetON-TFAP2C CDS. For generating NKX2.1 variants, naturally occurring mutations in NKX2.1 binding domain region was selected from Leiden Open Variation Database 3.0 ^22^ (www.lovd.nl/3.0) and two previously reported clinical cases^23,24^ – 1 amino acid deletion^22^ (p.R162del), two nonsense point mutations^22^ (p.Q175* and p.R178*), and two missense point mutations^23,24^ (p.I207F and p.I207M). NKX2.1 CDS harbouring each mutation was amplified and inserted by Infusion (638909, Takara) cloning into the tetON-NKX2.1/EF1a-TagRFP-2A-tet3G Dox-inducible lentiviral vector. NKX2.1 CDS lacking the entire DNA binding domain was inserted into the EF1a-TagRFP-2A-tet3G Dox-inducible lentiviral vector by Infusion cloning.

For the NKX2.1 or TFAP2C knock-down experiment, a modified Dox-inducible CRISPRi vector was gifted;^5^ N-terminal KRAB-dCas9 (a gift from Bruce Conklin, Addgene plasmid # 73498) fused with a destabilising domain, dihydrofolate reductase (DHFR) sequence that is only stabilised by trimethoprim (TMP) treatment, was sub-cloned into the EF1a-TagRFP-2A-tet3G Dox-inducible lentiviral vector.^5^ Treatment of 2 μg/ml Dox (Merck, D9891) with 10 nmol/L TMP (Merck, 92131) in the SN medium stabilizes the functional KRAB-dCas9 protein. Three gRNAs targeting NKX2.1^34^ were individually subcloned into gRNA lentivirus as follows: gRNA-1; 5’-GTCTGACGGCGGCAGAAGAG-3’, gRNA-2; 5’-GGACCAACAGTGCGGCCCCA-3’, gRNA-3; 5’-GAAATGAGCGAGCGAGTCTG-3’. The gRNAs for targeting TFAP2C: gRNA-1; 5’-GGCGGTCTTGACACTCGCGG-3’, gRNA-2; 5’-GTCGCCAGGACACACTGTTC-3’, gRNA-3; 5’-GGTCACTGGACACGCATCGG-3’.

Single cells dissociated from the organoids were infected and the infected cells were sorted by TagRFP and/or mNeonGreen fluorescent signal using FACS (Figure 5) after 48 hours of infection. The sorted TagRFP^+^ and/or mNeonGreen^+^ cells were cultured in the Matrigel in the absence of Dox or TMP for 1 week. After the cells were grown into a typical organoid, the Dox and/or TMP were added and culture continued for additional 2 weeks.

For transducing the N-terminal Dam-NKX2.1 fusion protein to the organoids, NKX2.1 CDS was inserted into the SFFV- mNeonGreen-Dam lentiviral vector by infusion cloning (see Table S4 for vector map). 200,000 CD44^+^ cells sorted from the 7-9 pcw organoids, CD36^+^/CD44^+^ cells sorted from the Lin^POS^ organoids, or single-cell dissociated alveolar organoids were infected with the lentivirus harbouring mNeoGreen-Dam only or mNeoGreen-Dam-NKX2.1 for 2 days.

### Immunostaining of organoids and lung tissues

For immunostaining of human lung tissue sections, the lungs were fixed in 4% paraformaldedyde (PFA; Merck, 158127) overnight, washed in PBS and 15%, 20% and 30% sucrose (w/v) in PBS before embedding in Optimum Cutting Temperature (OCT) medium (Merck, F4680). 12 μm thick frozen sections were collected and permeabilised using 0.3% Triton-X in PBS for 15 min. Antigen retrieval was performed by heating the slides in 10 mM Na-Citrate buffer at pH 6.0 in a microwave for 5 min. Then slides were treated with blocking solution containing 5% NDS, 1% Bovine Serum Albumin (BSA), 0.1% Triton-X in PBS at room temperature for 1 hour.

For whole-mount immunostaining of lung organoids, the Matrigel was completely removed from the cultured organoids using Cell Recovery Solution (Corning, 354253) followed by fixation in 4% PFA for 30 min on ice. After rinsing in PBS washing solution containing 0.2% (v/v) Triton X-100 and 0.5% (w/v) BSA, the samples were transferred to a round-bottom 96 well plate and incubated in permeabilization/blocking solution containing 0.2% (v/v) Triton X-100, 1% (w/v) BSA, and 5% normal donkey serum (NDS) in PBS, overnight at 4°C. For primary antibody treatment, the following antibodies were treated to the organoids and the tissue slices at 4°C overnight: proSFTPC (1:200; Merck, AB3786), E-cadherin (1: 500; Thermo Fisher Scientific, 13-1900), NKX2.1 (1:200; Millipore, 07-601), TFAP2C (1:200; Abcam, ab218107), CD44 (1:200; Thermo Fisher Scientific, 17-0441-82), CD36 (1:200; Proteintech, 18836-1-AP), alpha-smooth muscle actin (1:500; Thermo Fisher Scientific, MA1-06110), ACE2 (1:100; Abcam, ab108252), AXIN2 (1:200; R&D Systems, MAB6078), PDPN (1:200; R&D Systems, AF3670), CD31 (1:200; Abcam, ab9498), PDGFRA (1:200; Cell Signaling Technology, 3174), TP63 (1:200; Cell Signaling Technology, 13109), SOX2 (1: 500, R&D systems, AF2018), SOX9 (1: 500, Merck, AB5535), LAMP3 (1:100; Atlas Antibodies, HPA051467), HTII-280 (1:200; Terrace Biotech, TB-27AHT2-280), mature SFTPC (1:200; Seven Hills Bioreagents, WRAB-76694), proSFTPB (1:200; Seven Hills Bioreagents, WRAB-55522), ABCA3 (1:200; Seven Hills Bioreagents, WMAB-17G524), CD141 (1:100; PE-conjugated; BioLegend, 344104), ZO-1 (1:200; Thermo Fisher Scientific, 40-2200), GATA6 (1:200; Cell Signaling Technology, 5851), NOTUM (1:1000; Novus Biologicals, NBP2-94699), and KI67 (1:200; BD Biosciences, 550609). F-actin staining reagent (1 droplet in 200 μl; Thermo Fisher Scientific, R37110) was treated at room temperature for 30 min, followed by nuclei counter staining. After three washes with PBS, 97% (v/v) 2’–2’-thio-diethanol (TDE, Merck, 166782) was treated for clearing. Images were collected under Leica SP8 confocal microscope.

### *In situ* hybridization chain reaction (*in situ* HCR)

*In situ* HCR v3.0 was performed according to the manufacturer’s procedure (Molecular Instruments^36^). Probes were designed according to the protocol and amplifiers with buffers were purchased from Molecular Technologies. Sequence information of the probes for detecting *SFTPC, WNT2, NOTUM, WIF1, AXIN2, SOX9, FGFR4, TPPP3, SCGB3A2, SPOCK2*, and *TFAP2C* mRNA targets is listed in Table S3. Briefly, frozen human tissue sections were cut from 20 μm up to 100 μm from lungs fixed overnight in 4% PFA in DEPC-treated PBS and processed to cryoblocks. Lung sections were carefully rinsed in nuclease-free water, followed by 10 μg/mL proteinase K treatment (Thermo Fisher Scientific, AM2546), and 2 pmol of each probe was treated at 37°C overnight. After washing, the tissue was incubated with 6 pmol of the amplifiers at room temperature overnight for amplification. The amplifiers, consisting of a pair of hairpins conjugated to fluorophores, Alexa 546, 647, or 488, were snap-cooled separately and added at final 0.03 μM to the tissue. After removing excess hairpins in 5X SSC (sodium chloride sodium citrate) buffer (5X SSCT), containing 0.1% Triton X-100, nuclei were counter-stained with DAPI.

To combine *in situ* HCR with antibody immunostaining, the frozen human tissue sections from 20 μm up to 100 μm thickness were permeabilised using 0.3% Triton-X in DEPC-treated PBS for 3 min at room temperature. The lung sections were treated with probes at 37°C overnight. After washing in 5X SSCT, incubated with 6 pmol of the amplifiers at room temperature overnight for amplification. After washing three times in 5X SSCT, the tissues were treated with blocking solution containing 5% NDS, 1% BSA, 0.1% Triton-X in DEPC-treated PBS at room temperature for 1 hour and directly treated with a primary antibody against ACTA2 (1:500; Thermo Fisher Scientific, MA1-06110) or E-cadherin (1:200; Thermo Fisher Scientific, 13-1900) overnight at 4°C, followed by a secondary antibody treatment (1:500; Thermo Fisher Scientific, A10036) at room temperature for 3 hours. After washing three times in 5X SSCT, nuclei were stained with DAPI. Finally, the tissues were processed to 2’-2’-thio-diethanol (TDE, Sigma, 166782) for clearing and mounting: 10 %, 25 %, 50 % (v/v) TDE in 1x DEPC-treated PBS for 1 hour and 97% TDE overnight at 4°C. Images were collected under Leica SP8 confocal microscope.

### RNA extraction, cDNA synthesis, qRT-PCR analysis, and bulk RNA-sequencing

Organoids were removed from the Matrigel and lysed. Total RNA was extracted according to the RNeasy Mini Kit (Qiagen, 74004) protocol. For cells freshly purified from human lung tissues were directly lysed using 100 μl lysis buffer from PicoPure™ RNA Isolation Kit (Thermo Fisher Scientific, KIT0204). First Strand cDNA synthesis was performed using High-Capacity cDNA Reverse Transcription Kit (Applied Biosystems, 4368814). Then, cDNA was diluted 1:50 for qRT-PCR reaction (SYBR Green PCR Master Mix; Applied Biosystems, 4309155). Primer sequence information is listed in Table S3. Data is presented as fold change, calculated by ddCt method, using *ACTB* as housekeeping reference gene. For bulk RNA-seq, RNA quality was validated on Agilent 2200 Tapestation. The RNA-seq libraries were generated at the Cancer Research UK Cambridge Institute and sequenced on an Illumina HiSeq 4000. A list of differentially expressed genes was extracted using the counted reads and R package edgeR^51^ version 3.16.5 for the 3 pairwise comparisons (Table S1). GO biological processes term enrichment, KEGG pathway, and gene set enrichment analysis were performed using DAVID,^37^ Enrichr,^52^ and R package fgsea package,^39^ respectively.

### Immunoblotting

The organoid samples were harvested and lysed (RIPA buffer; Merck, R0278) after complete removal of the Matrigel and run on 12.5 % ~ 20 % SDS PAGE gels. Proteins on the gels were transferred onto PVDF membrane using BioRad Mini Trans-Blot system (BioRad, Mini Trans-Blot® Cell). The membranes were washed with pure water and blocked with 5% skimmed milk in 0.1% Tween-20/PBS (PBST) for 30 min at room temperature. Membranes were incubated with primary antibodies against NKX2.1 (1:200; Millipore, 07-601), proSFTPC (1:1000; Millipore, AB3786), mature SFTPC (1:1000; Seven Hills Bioreagents, WRAB-76694), mature SFTPB (1:1000; Seven Hills Bioreagents, WRAB-48604), NOTUM (1:1000; Novus Biologicals, NBP2-94699), and GAPDH (1:5000; Abcam, ab8245) in the blocking buffer overnight at 4 °C. After washing with PBST, secondary antibodies conjugated with fluorescence dyes (1:5000; anti-mouse IRDye ® 800CW and anti-rabbit IRDye 680RD; Abcam, ab216774 and ab216779, respectively) were treated at room temperature for 3 hours. The membranes were washed in PBST and developed using Li-Cor Odyssey imaging system.

### Activity-based protein profiling on SDS-PAGE gel

*In situ* gel-based activity-based protein profiling was performed according to the manufacturer’s instruction (ActivX™ TAMRA-FP Serine Hydrolase Probe, Thermo Fisher Scientific, 88318). 1 x 10^6^ of freshly isolated alveolar fibroblasts (PDGFRA^−^CD141^−^) and myofibroblasts (PDGFRA^−^CD141^−^) from late-stage lung tissues were lysed (Thermo Fisher Scientific, 78503), in the presence of a phosphatase inhibitor cocktail (1:100; Thermo Fisher Scientific, 78420), on ice for 10 min. 10 μg of lysate at 2 mg/mL concentration was treated with 1μM ABC99 (Cambridge Bioscience, CAY25858), a selective, irreversible NOTOM inhibitor, or DMSO only for 30 min at 37°C. 2 μM of the serine hydrolase FP probe was incubated for 30 min at room temperature. The reactions stopped by adding 4X Laemmli reducing sample buffer (BioRad, 1610747) and boiling for 5 minutes. The labelled proteins were loaded by SDS-PAGE, followed by fluorescent gel imaging.

### Chromatin immunoprecipitation

Chromatin immunoprecipitation (ChIP) was performed according to SimpleChIP® Chromatin immunoprecipitation protocol (Cell Signaling Technology, 9002). In brief, the organoids were harvested and enzymatically dissociated into single cells using TrypLE Express Enzyme (Thermo Fisher Scientific, 12605010). Then the cells were crosslinked with 1% formaldehyde for 15 min at room temperature and the reaction was quenched by glycine at a final concentration of 0.125 M. Chromatin was digested with 1 μl MNase (Cell Signaling Technologies, 10011S) for 20 min at 37°C, followed by sonication for 12 cycles of 30 seconds on and 30 seconds off using Biorupter (Diagenode, UCD-300), to length of an average size of 150-900 bp. 5 μg of digested chromatin samples was treated with antibodies against rabbit IgG (1:100; Cell Signaling Technology, 2729) or NKX2.1 (1:100; Millipore, 07-601). The amount of immunoprecipitated DNA was quantified by qPCR using primers specific for promoter regions of SFTPC, LAMP3, and SLC34A2. Fold enrichment values are presented as the fold-change over the level of ChIP with negative control IgG antibody (ChIP signal/IgG signal). Sequence information of the primers for targeting SFTPC, LAMP3, and SLC34A2 promoter regions is listed in Table S3.

### Bulk ATAC-sequencing

Genome-wide chromatin accessibility of lung organoids was assessed as previously described.^53^ In brief, 50,000 cells were harvested from organoids and lysed in lysis buffer (10 mM Tris-HCl, pH 7.4, 10 mM NaCl, 3 mM MgCl2, 0.1% (v/v) IGEPAL CA-630). The lysate was treated in 50 μL reactions with Nextera TDE1 transposase (Illumina, 15027865) for 30 min at 37°C. The purified DNA was amplified and indexed using Nextra DNA CD Indexes (Illumina, 20018707), and size distribution of the DNA libraries was analysed using High-sensitivity Qubit dsDNA Assay Kit (ThermoFisher, Q32851) and Agilent 2200 Tapestation. The libraries were sequenced on an Illumina HiSeq 4000. Peak calling was done using MACS2 algorithm^54^ (version 2.1.1) and further processed to extract differential peaks (Table S2). Then, the differential peak data was further used for analysing transcription factor motifs using HOMER^41^ software in combined with RNA-seq data.

### Electron microscopy imaging

The organoid samples were fixed in 2 % formaldehyde/2 % glutaraldehyde in 0.05 M sodium cacodylate buffer (NaCAC), pH 7.4, containing 2 mM calcium chloride (Merck, C27902) overnight at 4°C. After washing in 0.05 M NaCAC at pH 7.4, the samples were osmicated for 3 days at 4°C. After washing in deionised water (DIW), the samples were treated twice with 0.1 % (w/v) thiocarbohydrazide (Merck, 223220) in DIW for each 20 min and 1 hour at room temperature in the dark, followed by block-staining with uranyl acetate (2 % uranyl acetate in 0.05 M maleate buffer pH 5.5) for 3 days at 4°C. Then, the samples were dehydrated in a graded series of ethanol (50%/70%/95%/100%/100% dry) 100% dry acetone and 100% dry acetonitrile, three times in each for at least 5 min. Next, the samples were infiltrated with a 50:50 mixture of 100% dry acetonitrile/Quetol resin (TAAB, Q005) without BDMA (TAAB, B008) overnight, followed by 3 days in 100% Quetol without BDMA. The sample was infiltrated for 5 days in 100% Quetol resin with BDMA, exchanging the resin each day. The Quetol resin mixture is: 12 g Quetol 651, 15.7 g NSA (TAAB, N020), 5.7 g MNA (TAAB, M012) and 0.5 g BDMA. Samples were placed in embedding moulds and cured at 60°C for 3 days.

Thin sections were cut using an Ultracut E ultramicrotome (Leica) and mounted on melinex plastic coverslips. The coverslips were mounted on aluminium SEM stubs using conductive carbon tabs and the edges of the slides were painted with conductive silver paint. Then, the samples were sputter coated with 30 nm carbon using a Quorum Q150 T E carbon coater and imaged in a Verios 460 scanning electron microscope (FEI, Thermo Fisher Scientific) at 4 keV accelerating voltage and 0.2 nA probe current in back-scatter mode using the concentric backscatter detector in immersion mode at a working distance of 3.5-4 mm; 1,536 x 1,024 pixel resolution, 3 μs dwell time, 4 line integrations. Stitched maps were acquired using FEI MAPS software using the default stitching profile and 10% image overlap.

### Organoid single-cell RNA sequencing

Four biological replicates of the Lin^POS^ and alveolar organoids were harvested individually and enzymatically dissociated into single cells using TrypLE Express Enzyme. The cell suspension was passed through a 30 μm filter, pelleted, and resuspended in appropriate volume with 0.04% BSA/PBS, and single cell RNA seq was carried out according to 10X Chromium Single Cell 5’ Kits (v1). Library generation for 10x Genomics analysis were performed following the Chromium Single Cell 5′ Reagents Kits (10x Genomics) and sequenced on a NovaSeq 6000 S4 Flowcell (paired-end (PE), 150-bp reads) to achieve an average of 50,000 PE reads per cell. The single-cell RNA seq data were mapped (STARsolo^49^ v2.7.3a) to GRCh38 reference (v3.0.0; Ensembl 93), followed by cell calling post-processed with an implementation of EmptyDrops extracted from Cell Ranger 3.0.2.

### Targeted DamID sequencing

For Targeted DamID sequencing, genomic DNA was extracted from whole organoids or sorted cells and processed as previously described.^20^ Sequencing was performed as single end 100 bp reads by the Gurdon Institute NGS core facility using an illumina NovaSeq 6000^19^. Raw fastq files were analysed with a modified version of the damidseq_pipeline. Reads were mapped to a bowtie2 indexed GRCh38 genome assembly (hg38), binned into 5’-GATC-3’ fragments and each NKX2-1 fusion protein sample was normalised against a separate Dam-only replicate. Each organoid sample was treated as an individual replicate. For each of these replicates, the NKX2-1 fusion samples were normalized against a paired Dam-only control derived from the same biological sample (RPM normalization, 300 bp bins). Binding intensity values were quantile normalized across all replicates for each stage and back-transformed (“unlog”). Dam-only samples were analysed in parallel without normalization as a proxy for chromatin accessibility.^55^ For visualisation, files were converted to bigwig format using bedGraphToBigWig (v4) and imported into the Integrative Genomics Viewer (IGV v2.4.19). Macs2 (v2.1.2) was used to call broad peaks for each NKX2-1 fusion and Dam-only pair using the bam files generated by the damidseq_pipeline. Peaks were filtered to only those present in 2/3 replicates using bedtools (v2.26.0) and surpassing FDR<10^-5^ (Table S4). Distribution across genomic features was analysed and peak-gene associations were identified using GREAT.^43^ Binding intensities from genes associated with peaks were extracted from bedgraph files and averaged across gene bodies, including -1kb from the TSS. In order to derive binding patterns across time points, these genes were subjected to k-means clustering, with parameters optimised according to silhouettes calculated with R-cluster package (v2.0.7-1). Heatmaps were generated using the ggplot2 R package. *De novo* motif enrichment analysis was performed using AME package from MEME suite^44^ with the HOCOMOCO Human (v11) motif database as reference.

## Quantification and Statistical Analysis

Data are expressed as average ± standard deviation (SD). Statistical significance was evaluated by unpaired student’s *t* test, 1- or 2-way ANOVA with Tukey/Bonferroni/Dunnett comparison multiple comparison post-test; ns: not significant, *P<0.05, **P<0.01, ***P<0.001, and ****P<0.0001.

## Supplementary Material

Supplemental information can be found online at https://doi.org/10.1016/j.stem.2022.11.013.

Supplementary Material

## Highlights

- Human fetal lung tip progenitor cells commit to alveolar fate from 15 pcw *in vivo*
- Late-stage tip-derived organoids readily differentiated to mature cell fates
- NOTUM^+^ myofibroblasts spatially pattern differentiating human alveolar-type-2 cells
- Lineage-determining TFs facilitate organoid modeling of neonatal lung disease

## In brief

Lim and colleagues identify the timing of alveolar-fate specification in human fetal lungs. Their stage-specific organoids allow functional analysis of mechanisms controlling fate maintenance and differentiation in the developing alveolar niche, facilitating the modeling of neonatal lung disease.
