# Supplementary material for: Organoid modeling of human fetal lung alveolar development reveals mechanisms of cell fate patterning and neonatal respiratory disease
Source: Cell Stem Cell. Author manuscript; Available in PMC 2025 Dec 9. (PMC7618456; doi:10.1016/j.stem.2022.11.013)
Supplement: Supplementary Material [file EMS211309-supplement-Supplementary_Material.zip › 1-s2.0-S193459092200460X-mmc1.pdf]

Cell Stem Cell, Volume *30*

Supplemental Information

Organoid modeling of human fetal lung alveolar development reveals mechanisms of cell fate patterning and neonatal respiratory disease

Kyungtae Lim, Alex P.A. Donovan, Walfred Tang, Dawei Sun, Peng He, J. Patrick Pett, Sarah A. Teichmann, John C. Marioni, Kerstin B. Meyer, Andrea H. Brand, and Emma L. Rawlins

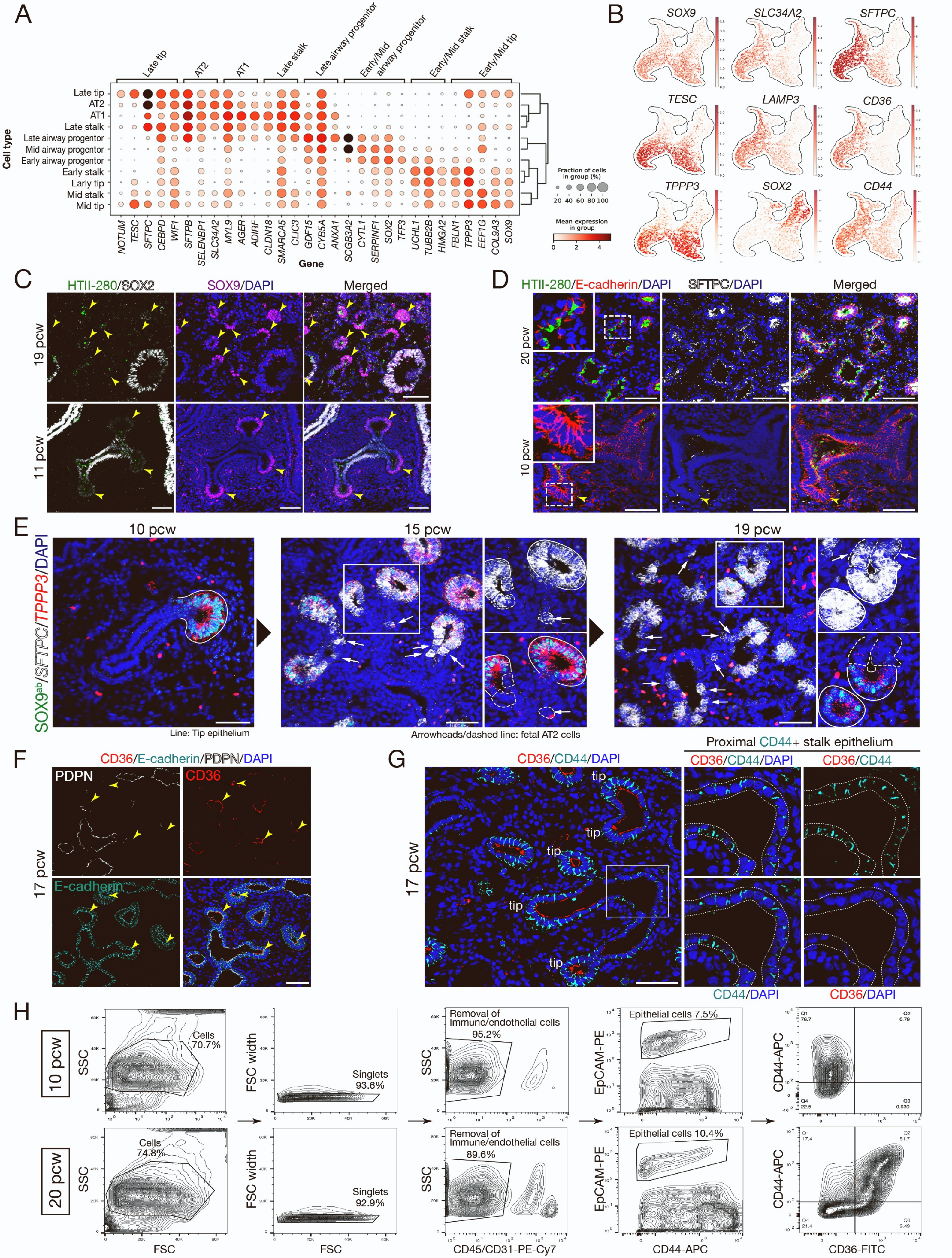

A
Late tip
AT2
AT1
Late stalk
Late airway progenitor
Early/Mid airway progenitor
Early/Mid stalk
Early/Mid tip
Cell type
Late tip
AT2
AT1
Late stalk
Late airway progentor
Mid airway progentor
Early airway progentor
Early stalk
Early tip
Mid stalk
Mid tip
NOTUM TESC SFTPC CEBPD WIF1 SFTPB SELENBP1 SLC34A2 MYL9 AGER ADIRF CLDN18 SMARCA5 CLIC3 GDF15 CYB5A ANXA1 SCGB3A2 CYTL1 SERPINF1 SOX2 TFF3 UCHL1 TUBB2B HMGA2 FBLN1 TPPP3 EEF1G COL9A3 SOX9
Gene
Fraction of cells in group (%)
20 40 60 80 100
Mean expression in group
0 2 4
B
SOX9
SLC34A2
SFTPC
TESC
LAMP3
CD36
TPPP3
SOX2
CD44
C
HTII-280/SOX2
SOX9/DAPI
Merged
19 pcw
11 pcw
D
HTII-280/E-cadherin/DAPI
SFTPC/DAPI
Merged
20 pcw
10 pcw
E
10 pcw
15 pcw
19 pcw
SOX9ab/SFTPC/TPPP3/DAPI
Line: Tip epithelium
Arrowheads/dashed line: fetal AT2 cells
F
CD36/E-cadherin/PDPN/DAPI
PDPN
CD36
E-cadherin
17 pcw
G
CD36/CD44/DAPI
Proximal CD44+ stalk epithelium
CD36/CD44/DAPI
CD36/CD44
tip
17 pcw
CD44/DAPI
CD36/DAPI
H
10 pcw
20 pcw
SSC
FSC
Cells 70.7%
Cells 74.8%
FSC width
Singlets 93.6%
Singlets 92.9%
Removal of Immune/endothelial cells 95.2%
Removal of Immune/endothelial cells 89.6%
CD45/CD31-PE-Cy7
EpCAM-PE
Epithelial cells 7.5%
Epithelial cells 10.4%
CD44-APC
Q1 76.7
Q2 0.79
Q4 22.5
Q3 0.030
Q1 17.4
Q2 51.7
Q4 21.4
Q3 9.49
CD36-FITC

Figure S1. Characterization of the lung tip epithelium at the late stage. Related to Figure 1.

(A and B) Dot plot (A) and UMAP (B) showing representative genes of each cell type in distal human lung tissues from 5 to 22 pcw.

(C and D) Human fetal lung at early-mid and late stages; 11, 19 pcw (C) and 10, 20 pcw (D). Tip epithelium (arrowheads) is marked by E-cadherin, SFTPC, HTII-280, and SOX9. SOX2, airway epithelium (C). DAPI, nuclei. Scale bars, 50 µm.

(E) Time course analysis of human fetal lung tissues at early stage, 10 pcw, and late stages, 15 and 19 pcw, by *in situ* HCR (*SFTPC* and *TPPP3*) and antibody immunostaining ($SOX9^{ab}$). The early-stage tip epithelium at 10 pcw expresses SOX9 and *TPPP3*, and gains the expression of the alveolar lineage marker, *SFTPC*, at late stages, 15 and 19 pcw. The $SFTPC^{+}$ stalk cells (hereafter, fetal AT2 cells) at 15 and 19 pcw lack SOX9 and *TPPP3* expression ($SOX9^{-}TPPP3^{-}SFTPC^{+}$). Lines, tip epithelium. Arrowheads/dashed lines, fetal AT2 cells. DAPI, nuclei. Scale bars, 50 µm.

(F and G) Frozen sections of human fetal lung tissues at 17 pcw. Stained for CD36, E-cadherin and PDPN (F) and CD36, CD44 (G). Arrowheads indicate $CD36^{+}PDPN^{-}$ tips. Inset (G) shows a CD44+, CD36- stalk epithelial region. DAPI, nuclei. Scale bars, 50 µm.

(H) Flow cytometry to isolate the human lung tip epithelial population at 10 (*upper*) and 20 pcw (*lower*). Note that the apparent CD44-,CD36+ population on the bottom-right FACs plot, quadrant 3, cannot be located in the tissue by immunostaining and additional controls show that it is likely to be a FACs-artefact caused by differential sensitivity of the CD36 and CD44 epitopes to digestion by the enzymes used to generate a single cell suspension.

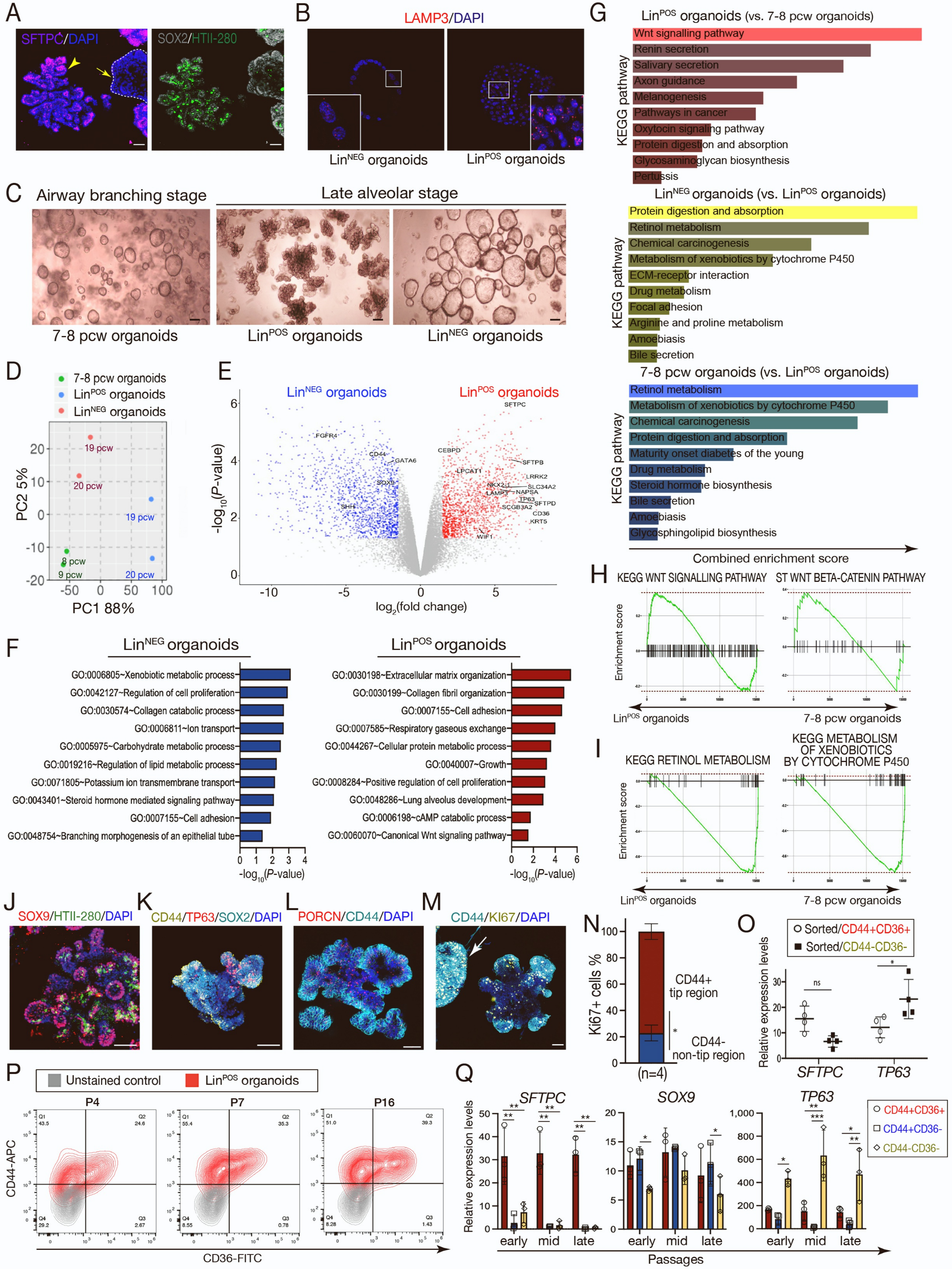
A
SFTPC/DAPI
SOX2/HTII-280
B
LAMP3/DAPI
LinNEG organoids
LinPOS organoids
C
Airway branching stage
Late alveolar stage
7-8 pcw organoids
LinPOS organoids
LinNEG organoids
D
7-8 pcw organoids
LinPOS organoids
LinNEG organoids
PC2 5%
PC1 88%
19 pcw
20 pcw
8 pcw
9 pcw
E
LinNEG organoids
LinPOS organoids
-log10(P-value)
log2(fold change)
F
LinNEG organoids
GO:0006805~Xenobiotic metabolic process
GO:0042127~Regulation of cell proliferation
GO:0030574~Collagen catabolic process
GO:0006811~Ion transport
GO:0005975~Carbohydrate metabolic process
GO:0019216~Regulation of lipid metabolic process
GO:0071805~Potassium ion transmembrane transport
GO:0043401~Steroid hormone mediated signaling pathway
GO:0007155~Cell adhesion
GO:0048754~Branching morphogenesis of an epithelial tube
LinPOS organoids
GO:0030198~Extracellular matrix organization
GO:0030199~Collagen fibril organization
GO:0007155~Cell adhesion
GO:0007585~Respiratory gaseous exchange
GO:0044267~Cellular protein metabolic process
GO:0040007~Growth
GO:0008284~Positive regulation of cell proliferation
GO:0048286~Lung alveolus development
GO:0006198~cAMP catabolic process
GO:0060070~Canonical Wnt signaling pathway
-log10(P-value)
G
LinPOS organoids (vs. 7-8 pcw organoids)
Wnt signalling pathway
Renin secretion
Salivary secretion
Axon guidance
Melanogenesis
Pathways in cancer
Oxytocin signalling pathway
Protein digestion and absorption
Glycosaminoglycan biosynthesis
Pertussis
LinNEG organoids (vs. LinPOS organoids)
Protein digestion and absorption
Retinol metabolism
Chemical carcinogenesis
Metabolism of xenobiotics by cytochrome P450
ECM-receptor interaction
Drug metabolism
Focal adhesion
Arginine and proline metabolism
Amoebiasis
Bile secretion
7-8 pcw organoids (vs. LinPOS organoids)
Retinol metabolism
Metabolism of xenobiotics by cytochrome P450
Chemical carcinogenesis
Protein digestion and absorption
Maturity onset diabetes of the young
Drug metabolism
Steroid hormone biosynthesis
Bile secretion
Amoebiasis
Glycosphingolipid biosynthesis
KEGG pathway
Combined enrichment score
H
KEGG WNT SIGNALLING PATHWAY
ST WNT BETA-CATENIN PATHWAY
Enrichment score
LinPOS organoids
7-8 pcw organoids
I
KEGG RETINOL METABOLISM
KEGG METABOLISM OF XENOBIOTICS BY CYTOCHROME P450
J SOX9/HTII-280/DAPI
K CD44/TP63/SOX2/DAPI
L PORCN/CD44/DAPI
M CD44/KI67/DAPI
N
Ki67+ cells %
CD44+ tip region
CD44- non-tip region
(n=4)
O
Sorted/CD44+CD36+
Sorted/CD44-CD36-
Relative expression levels
SFTPC
TP63
P
Unstained control
LinPOS organoids
P4
P7
P16
CD44-APC
CD36-FITC
Q
SFTPC
SOX9
TP63
Relative expression levels
CD44+CD36+
CD44+CD36-
CD44-CD36-
early
mid
late
Passages

Figure S2. Characterization of the late-stage lung tip organoids. Related to Figure 2.

(A and B) Immunofluorescence analysis of the Lin^{POS} (arrowhead) and Lin^{NEG} organoids (arrow) at passage 1 cultured in the self-renewing medium, showing the alveolar lineage markers, SFTPC, HTII-280 (A) and LAMP3 (B, *lower* panel) were expressed in the Lin^{POS} organoids, but not in the Lin^{NEG} organoids. DAPI, nuclei. Scale bar, 50 µm.

(C) Morphology of 7-8 pcw organoids derived from airway branching stage, and the Lin^{POS} and Lin^{NEG} organoids from late alveolar stage. The Lin^{POS} and Lin^{NEG} organoids grown from $EPCAM^{+}$ tip epithelial cells were manually separated and cultured in the self-renewing culture condition.

(D) Principal component analysis of bulk-RNA seq data using 7-8 pcw organoids, Lin^{POS} and Lin^{NEG} organoids.

(E) Volcano plot showing differentially expressed genes between Lin^{POS} organoids (*red*) versus Lin^{NEG} organoids (*dark blue*); $log_2FC > 4$.

(F) Gene ontology (GO) enrichment analysis performed for biological process (BP)- associated GO terms on the differentially expressed genes between the Lin^{POS} and Lin^{NEG} organoids; $log_2FC > 4$.

(G) KEGG pathway analysis using Enrichr. Length of coloured bars indicates combined enrichment score by adjusted p-value < 0.05.

(H and I) Gene set enrichment (GSEA) analysis of the differentially expressed genes of the Lin^{POS} organoids (H) and 7-8 pcw organoids (I).

(J-M) Immunofluorescence images of the Lin^{NEG} and Lin^{POS} organoids originating from sorted $CD44^{+}CD36^{+}$ tip epithelium from 20 pcw lung. Antibodies against SOX9, HTII-280 (J), CD44, TP63, SOX2 (K), CD44, PORCN (L) and CD44, KI67 (M). Arrow (M) indicates a Lin^{NEG} organoid. DAPI indicates nuclei. Scale bar, 50 µm.

(N) The percentage of $KI67^{+}$ cells in the $CD44^{+}$ tip and $CD44^{-}$ non-tip regions are represented as mean ± SD of biological 4 replicates.

(O) Expression of lineage markers was investigated by qRT-PCR in the Lin^{POS} organoids and airway-like spheres at passage 1 derived from the $CD44^{+}CD36^{+}$ or $CD44^{-}CD36^{-}$ passage zero subpopulations respectively. Data was normalized to $EPCAM^{+}$ cells freshly sorted from 20 pcw tip tissues and represented as mean ± SD of 4 biological replicates. Significance was evaluated by 2-way ANOVA with Bonferroni multiple comparison post-test; ns: not significant, *$P<0.05$.

(P and Q). FACS analysis (P) of the cellular composition of Lin^{POS} organoid lines at multiple passages: early passage (2-5); mid passage (6-12); late passages (14-20) by CD36 (FITC) and CD44 (APC) and gene expression profiling (Q) of each fraction, $CD44^{+}CD36^{+}$, $CD44^{+}CD36^{-}$, and $CD44^{-}CD36^{-}$, by qRT-PCR. Red, stained. Gray, unstained control. Data normalized to fresh EPCAM+ cells from 20 pcw distal tissues; mean ± SD of biological 3 replicates. Significance was evaluated by 2-way ANOVA with Tukey multiple comparison post-test; * $P<0.05$, ** $P<0.01$, *** $P<0.001$, and ****$P<0.0001$.

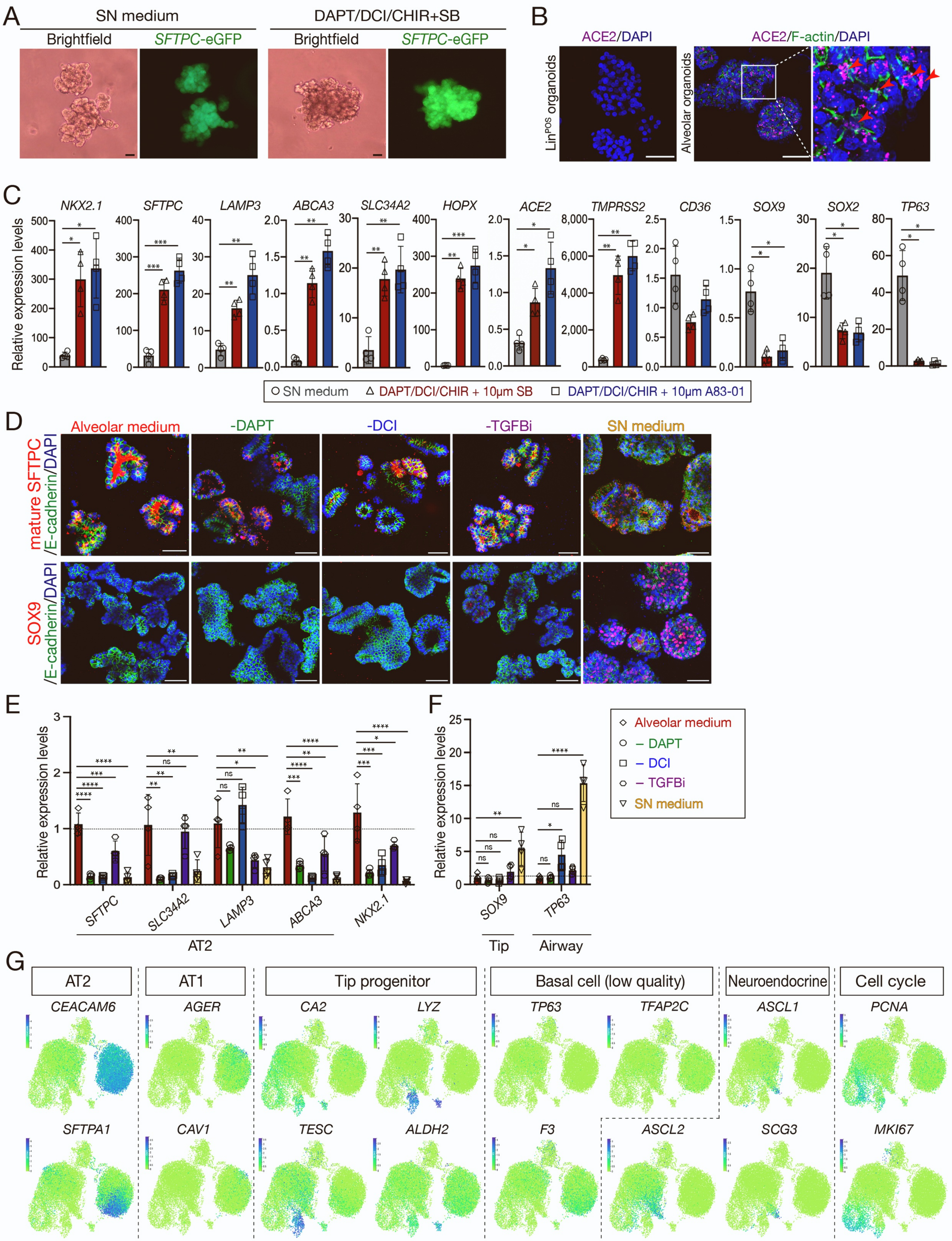

A
SN medium
DAPT/DCI/CHIR+SB
Brightfield
SFTPC-eGFP
Brightfield
SFTPC-eGFP
B
ACE2/DAPI
ACE2/F-actin/DAPI
Lin^POS organoids
Alveolar organoids
C
NKX2.1
SFTPC
LAMP3
ABCA3
SLC34A2
HOPX
ACE2
TMPRSS2
CD36
SOX9
SOX2
TP63
Relative expression levels
SN medium
DAPT/DCI/CHIR + 10μm SB
DAPT/DCI/CHIR + 10μm A83-01
D
Alveolar medium
-DAPT
-DCI
-TGFBi
SN medium
mature SFTPC/E-cadherin/DAPI
SOX9/E-cadherin/DAPI
E
Relative expression levels
SFTPC
SLC34A2
LAMP3
ABCA3
NKX2.1
AT2
F
Relative expression levels
SOX9
TP63
Tip
Airway
Alveolar medium
– DAPT
– DCI
– TGFBi
SN medium
G
AT2
AT1
Tip progenitor
Basal cell (low quality)
Neuroendocrine
Cell cycle
CEACAM6
AGER
CA2
LYZ
TP63
TFAP2C
ASCL1
PCNA
SFTPA1
CAV1
TESC
ALDH2
F3
ASCL2
SCG3
MKI67

Figure S3. Alveolar differentiation of the cultured late stage tip cells. Related to Figure 3.

(A) Morphology and fluorescent images of the Lin^{POS} organoids cultured in control SN medium, or in DAPT/DCI/CHIR with SB, for 1 week.

(B) Immunofluorescent analysis of ACE2 in the Lin^{POS} organoids and alveolar organoids. Phalloidin (F-actin) marks apical membrane of epithelial cells in the organoids. Scale bar, 50 µm.

(C) qRT-PCR of Lin^{POS} organoids cultured in the SN medium or in DAPT/DCI/CHIR with SB, or A83-01, for 1 week. Data were normalized to $EPCAM^{+}$ cells freshly isolated from 20 pcw tip tissues; mean ± SD of four biological replicates. Significance was evaluated by 1-way ANOVA with Tukey multiple comparison post-test; *P<0.05, **P<0.01, ***P<0.001.

(D-F) Fluorescence (D) and qRT-PCR (E, F) analysis of the alveolar organoids upon withdrawal of each component, DAPT, DCI, or TGFBi (SB431542), from the alveolar differentiation medium for 1 week. Expression level in SN medium is shown as the negative control. Data normalized to the alveolar medium condition; mean ± SD, n = 3. Significance evaluated by 1-way ANOVA with Tukey multiple comparison post-test; ns: not significant, *P<0.05, **P<0.01, ***P<0.001, ****P<0.0001. DAPI, nuclei. Scale bar, 50 µm.

(G) UMAP plots showing transcript expression of marker genes specific to AT2, AT1, tip progenitor, basal cell, neuroendocrine lineages and cell cycle.

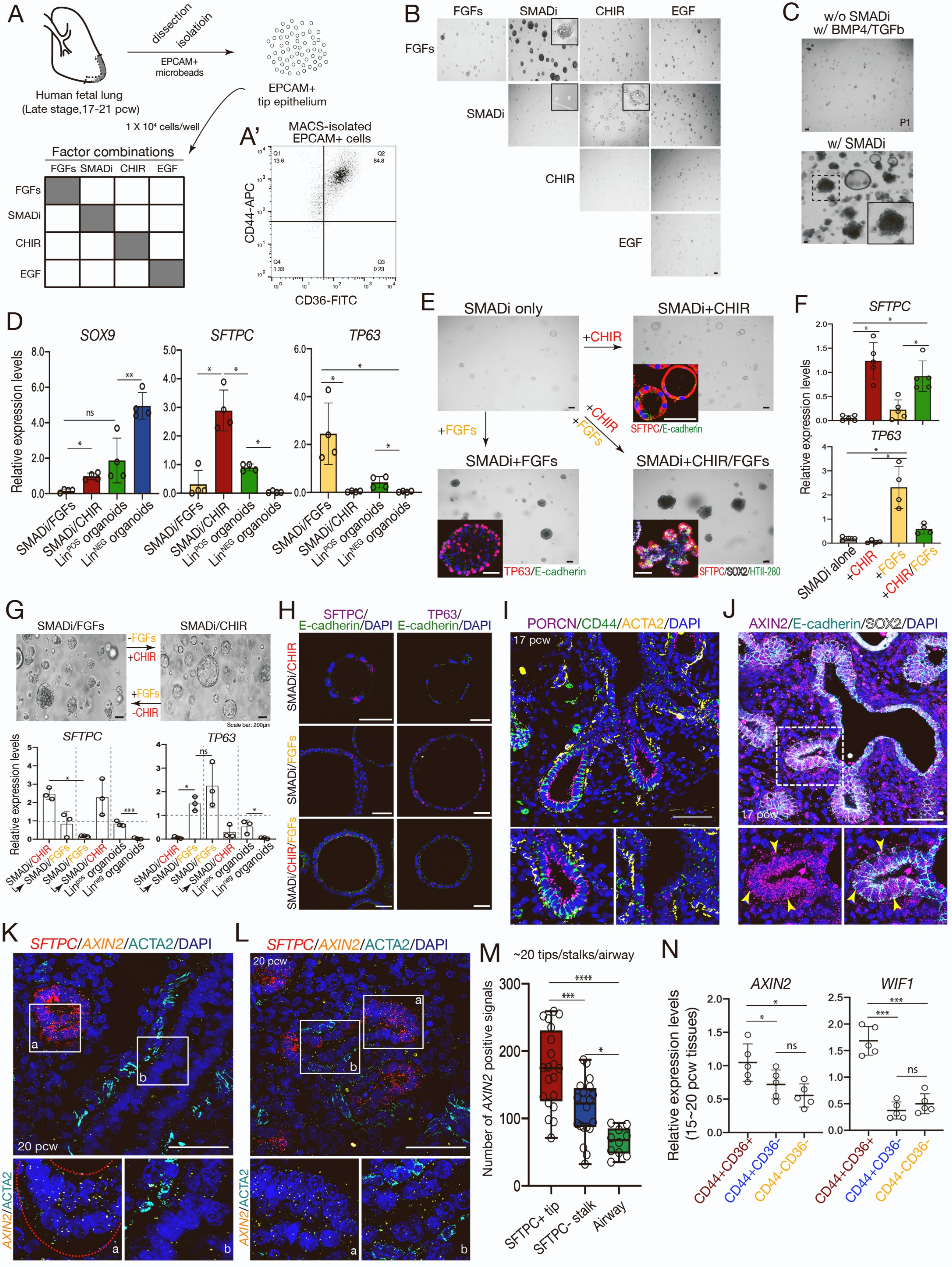
A
dissection
isolatioin
EPCAM+ microbeads
Human fetal lung
(Late stage,17-21 pcw)
EPCAM+ tip epithelium
1 X 10⁴ cells/well
Factor combinations
FGFs SMADi CHIR EGF
FGFs
SMADi
CHIR
EGF
A'
MACS-isolated EPCAM+ cells
Q1 13.6
Q2 84.8
Q4 1.33
Q3 0.23
CD44-APC
CD36-FITC
B
FGFs SMADi CHIR EGF
FGFs
SMADi
CHIR
EGF
C
w/o SMADi w/ BMP4/TGFb
P1
w/ SMADi
D
SOX9
SFTPC
TP63
Relative expression levels
ns
SMADi/FGFs
SMADi/CHIR
Lin^POS organoids
Lin^NEG organoids
E
SMADi only
+CHIR
SMADi+CHIR
SFTPC/E-cadherin
+FGFs
+CHIR +FGFs
SMADi+FGFs
TP63/E-cadherin
SMADi+CHIR/FGFs
SFTPC/SOX2/HTII-280
F
SFTPC
TP63
Relative expression levels
SMADi alone
+CHIR
+FGFs
+CHIR/FGFs
G
SMADi/FGFs
-FGFs
+CHIR
SMADi/CHIR
+FGFs
-CHIR
Scale bar: 200μm
SFTPC
TP63
Relative expression levels
ns
SMADi/CHIR
SMADi/FGFs
SMADi/FGFs
SMADi/CHIR
Lin^pos organoids
Lin^neg organoids
H
SFTPC/ E-cadherin/DAPI
TP63/ E-cadherin/DAPI
SMADi/CHIR
SMADi/FGFs
SMADi/CHIR/FGFs
I
PORCN/CD44/ACTA2/DAPI
17 pcw
J
AXIN2/E-cadherin/SOX2/DAPI
17 pcw
K
SFTPC/AXIN2/ACTA2/DAPI
20 pcw
AXIN2/ACTA2
a
b
L
SFTPC/AXIN2/ACTA2/DAPI
20 pcw
AXIN2/ACTA2
a
b
M
~20 tips/stalks/airway
Number of AXIN2 positive signals
SFTPC+ tip
SFTPC- stalk
Airway
N
AXIN2
WIF1
Relative expression levels (15~20 pcw tissues)
ns
CD44+CD36+
CD44+CD36-
CD44-CD36-

Figure S4. High plasticity of the late-stage tip epithelial cells in response to the signalling cues. Related to Figure 4.

(A) Diagram showing *in vitro* culture of the freshly isolated tip epithelial cells for 2 weeks in single or pairwise combinations of signalling cues: FGFs (FGF7, FGF10), SMADi (Noggin, SB431542), CHIR (CHIR99021) and EGF. (A') The isolated $EPCAM^{+}$ tip epithelial cells from the distal lung tissue at 20 pcw were analysed by CD44 and CD36 expression using FACS.

(B) Morphology of the tip organoids cultured in different culture conditions for 2 weeks. Representative image from 1 biological replicate is shown; n=4 biological replicates in total. Scale bar, 200 µm.

(C) Tip epithelial cells cultured with, or without, SMAD inhibition for 3 weeks. The self-renewal medium condition (w/ SMADi; *lower* panel) was used for positive control. Scale bar, 200 µm.

(D) Relative mRNA levels of *SOX9*, *SFTPC* and *TP63* measured by qRT-PCR. Normalized to a Lin^{POS} organoid line; mean ± SD of 4 independent biological replicates. Significance was evaluated by 1-way ANOVA with Tukey multiple comparison post-test; ns: not significant, *$P<0.05$ and **$P<0.01$.

(E and F) After 2 weeks growing in SMADi conditions, cells were sequentially transferred to culture medium containing CHIR, or FGFs, or CHIR/FGFs. After a further 2 weeks of exposure to the different culture conditions, the epithelial organoids were stained with lineage makers including SFTPC or TP63 (E) and relative mRNA levels of *SFTPC* and *TP63* were measured by qRT-PCR (F). Data were normalized to the Lin^{POS} organoids; mean ± SD of at least 4 biological replicates. Significance was evaluated by 1-way ANOVA with Tukey multiple comparison post-test; ns: not significant, *$P<0.05$, **$P<0.01$ and ***$P<0.001$. Scale bar, 200 µm.

(G) Dynamic cell fate plasticity of the tip epithelial cells depending on Wnt and FGF signals. The morphology of the epithelial organoids was changed when the culture conditions were switched from SMADi/FGFs to SMADi/CHIR, or vice versa (upper panels). The gene expression profile (lower panels) was analysed by qRT-PCR following 2 weeks exposure to the 2nd medium. Data were normalized to freshly isolated Lin^{POS} organoids; mean ± SD of 3 biological replicates. Significance was evaluated by 1-way ANOVA with Tukey multiple comparison post-test; ns: not significant, *$P<0.05$, **$P<0.01$, and ***$P<0.001$.

(H) Immunofluorescence analysis of 8 pcw organoids in the different conditions at passage 0. Antibodies against SFTPC, TP63, E-cadherin and SOX2 were used. Scale bar, 50 µm.

(I-M) Frozen sections of human fetal lung tissues at 17 pcw (I,J) and 20 pcw (K,L) were immunostained for PORCN, CD44, and ACTA2 (I), AXIN2, E-cadherin and SOX2 (J), or ACTA2 followed by *in situ* HCR for *SFTPC* and *AXIN2* (K, tip (a) and airway (b); L, tip (a) and stalk (b)). Arrowheads (J) indicate $AXIN2^{+}$ tip epithelial cells. Red dashed line in the inset (K) indicates $SFTPC^{+}$ tip epithelial cells. *AXIN2* signals were counted from 20 areas of tips and stalks and 10 areas of airway across 3 independent lung tissues at 18-20 pcw (M). Significance was evaluated by 1-way ANOVA

with Tukey multiple comparison post-test; ns: not significant, *$P<0.05$, **$P<0.01$, ***$P<0.001$ and ****$P<0.0001$. Scale bar, 50 μm.

(N) Gene expression profile of the freshly isolated lung epithelial cells from the late stage human lung tissues sorted by $CD44^+CD36^+$, $CD44^+CD36^-$ and $CD44^-CD36^-$. Data normalized to freshly isolated $EPCAM^+$ cells from 20 pcw tip tissues; mean ± SD of 5 biological replicates aged from 15~20 pcw. Significance was evaluated by 1-way ANOVA with Tukey multiple comparison post-test; ns: not significant, *$P<0.05$, **$P<0.01$ and ***$P<0.001$.

DAPI indicates nuclei.

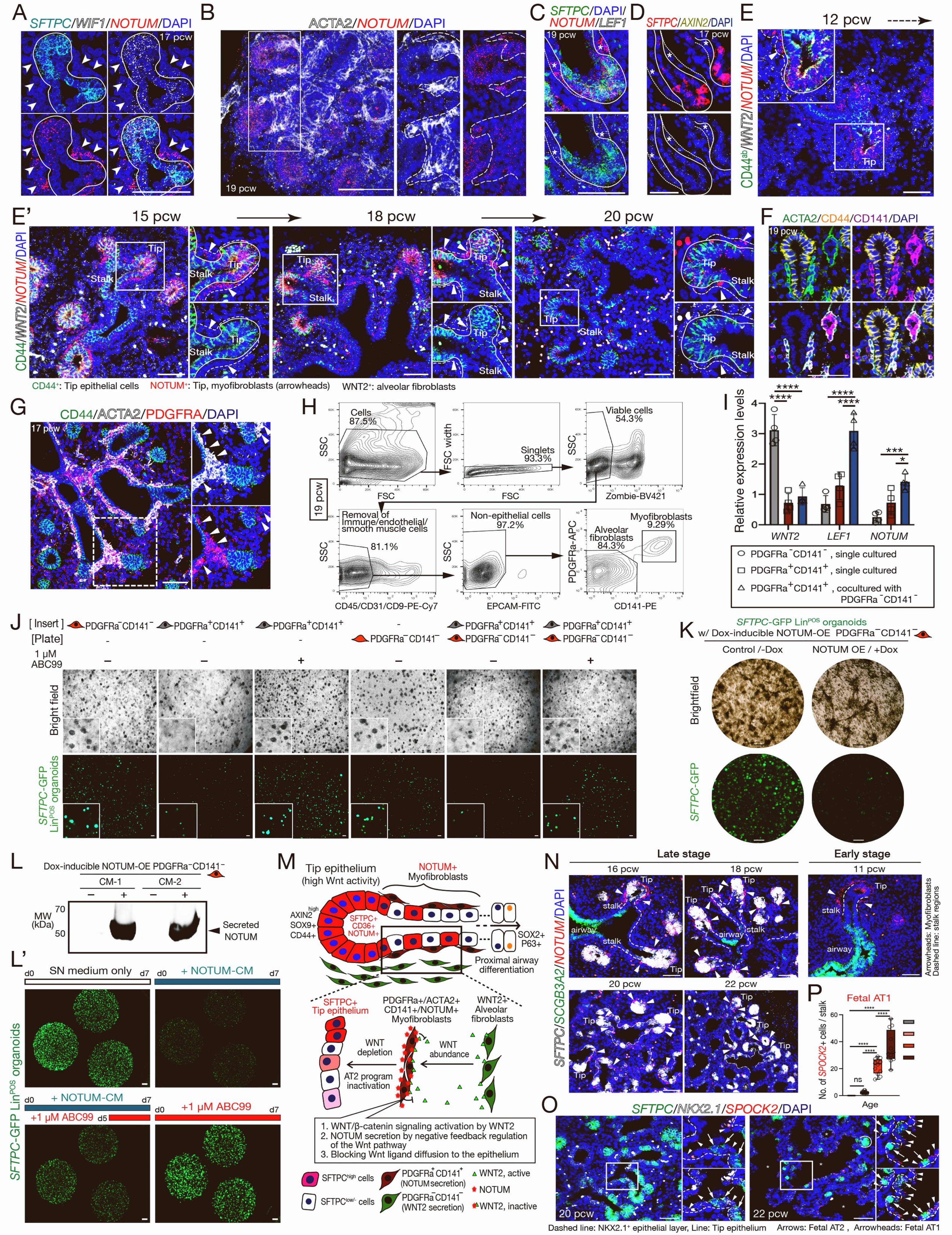
A
SFTPC/WIF1/NOTUM/DAPI
17 pcw
B
ACTA2/NOTUM/DAPI
19 pcw
C
SFTPC/DAPI/NOTUM/LEF1
19 pcw
D
SFTPC/AXIN2/DAPI
17 pcw
E
12 pcw
CD44ab/WNT2/NOTUM/DAPI
Tip
E'
15 pcw
18 pcw
20 pcw
CD44/WNT2/NOTUM/DAPI
Tip
Stalk
CD44+: Tip epithelial cells
NOTUM+: Tip, myofibroblasts (arrowheads)
WNT2+: alveolar fibroblasts
F
ACTA2/CD44/CD141/DAPI
19 pcw
G
CD44/ACTA2/PDGFRA/DAPI
17 pcw
H
19 pcw
Cells 87.5%
Singlets 93.3%
Viable cells 54.3%
SSC
FSC
FSC width
Zombie-BV421
Removal of Immune/endothelial/smooth muscle cells
81.1%
Non-epithelial cells 97.2%
Alveolar fibroblasts 84.3%
Myofibroblasts 9.29%
CD45/CD31/CD9-PE-Cy7
EPCAM-FITC
PDGFRa-APC
CD141-PE
I
Relative expression levels
WNT2
LEF1
NOTUM
PDGFRa−CD141−, single cultured
PDGFRa+CD141+, single cultured
PDGFRa+CD141+, cocultured with PDGFRa−CD141−
J
[Insert]
[Plate]
1 μM ABC99
PDGFRa−CD141−
PDGFRa+CD141+
Bright field
SFTPC-GFP LinPOS organoids
K
SFTPC-GFP LinPOS organoids
w/ Dox-inducible NOTUM-OE PDGFRa−CD141−
Control /-Dox
NOTUM OE / +Dox
Brightfield
SFTPC-GFP
L
Dox-inducible NOTUM-OE PDGFRa−CD141−
CM-1
CM-2
MW (kDa)
70
50
Secreted NOTUM
L'
SN medium only
+ NOTUM-CM
+1 μM ABC99
d0
d5
d7
SFTPC-GFP LinPOS organoids
M
Tip epithelium (high Wnt activity)
NOTUM+ Myofibroblasts
AXIN2high
SOX9+
CD44+
SFTPC+ CD36+ NOTUM+
SOX2+ P63+
Proximal airway differentiation
SFTPC+ Tip epithelium
PDGFRa+/ACTA2+ CD141+/NOTUM+ Myofibroblasts
WNT2+ Alveolar fibroblasts
WNT depletion
WNT abundance
AT2 program inactivation
1. WNT/β-catenin signaling activation by WNT2
2. NOTUM secretion by negative feedback regulation of the Wnt pathway
3. Blocking Wnt ligand diffusion to the epithelium
SFTPChigh cells
SFTPClow/- cells
PDGFRa+CD141+ (NOTUM secretion)
PDGFRa−CD141− (WNT2 secretion)
WNT2, active
NOTUM
WNT2, inactive
N
Late stage
Early stage
16 pcw
18 pcw
11 pcw
20 pcw
22 pcw
SFTPC/SCGB3A2/NOTUM/DAPI
Tip
stalk
airway
Arrowheads: Myofibroblasts
Dashed line: stalk regions
P
Fetal AT1
No. of SPOCK2+ cells / stalk
Age
ns
O
SFTPC/NKX2.1/SPOCK2/DAPI
20 pcw
22 pcw
Dashed line: NKX2.1+ epithelial layer, Line: Tip epithelium
Arrows: Fetal AT2, Arrowheads: Fetal AT1

Figure S5. Wnt-responsive NOTUM$^+$ myofibroblasts in the distal human fetal lung pattern differentiating alveolar epithelium. Related to Figure 4.

(A-D) Fluorescence imaging of human fetal lung sections at 17 (A, D) and 19 pcw (B, C), *in situ* HCR and immunostaining. (A) *SFTPC*, *WIF1*, *NOTUM*. (B) ACTA2, *NOTUM.* (C) *SFTPC*, *NOTUM, LEF1*. (D) *SFTPC*, *AXIN2*. Arrowheads and asterisks indicate ACTA2$^+$ *NOTUM*$^+$ myofibroblasts. Lines and dashed lines indicate the boundaries of epithelial cells and myofibroblasts, respectively.

(E) Time course analysis of distal human lung tissues at 12, 15, 18, and 20 pcw, using *in situ* HCR and immunostaining. *WNT2*, alveolar fibroblasts, white; *NOTUM*, myofibroblasts, red; CD44, tip epithelium, green. Arrowheads indicate myofibroblast population at the adjacent tip regions. Lines and dashed lines indicate the boundaries of epithelial cells and myofibroblasts, respectively.

(F, G) Identification of a combination of surface antigens, CD141 and PDGFRA, targeting the myofibroblasts, by immunostaining. (F) ACTA2, CD44, CD141. (G) CD44, ACTA2, PDGFRA.

(H) Flow cytometry to isolate alveolar fibroblasts and myofibroblasts from the late-stage lung at 19 pcw.

(I) Relative mRNA levels from myofibroblasts and alveolar fibroblasts cultured alone, or cocultured in transwells. Data were normalized to the whole freshly isolated lung fibroblast population; mean ± SD of biological 4 replicates. Significance was evaluated by unpaired student *t*-test; *P<0.05, **P<0.01, ***P<0.001.

(J) *In vitro* coculture assay of *SFTPC*-GFP$^+$ Lin^{POS} organoids with freshly isolated PDGFRA$^-$CD141$^-$ alveolar fibroblasts and/or PDGFRA$^+$CD141$^+$ myofibroblasts. 1µM ABC99 was used to inhibit NOTUM activity. Scale bar, 100 µm.

(K) Coculture of the *SFTPC*-GFP$^+$ Lin^{POS} organoids with the alveolar fibroblasts overexpressing doxycycline (Dox)-inducible NOTUM (NOTUM-OE), in DMEM/FBS 2% medium for 1 week. Scale bar, 100 µm.

(L, L') Conditioned medium (CM) was collected every 2 days for 1 week from the cultured PDGFRA$^-$ CD141$^-$ NOTUM-OE alveolar fibroblasts in the presence or absence of Dox and concentrated using 30K MWCO Concentrator (Thermo Fisher; 88529). (L) Western blot detection of the secreted NOTUM in the CM. DMEM+FBS 2% medium were used for the CM preparation; N = 2. (L') The *SFTPC*-GFP Lin^{POS} organoids were treated with/without the CM in the presence or absence of 1 µM ABC99, in the self-renewal (SN) medium, for 1 week. Scale bar, 100 µm.

(M) Summary diagram showing the spatial regulation of Wnt signalling mediated by ACTA2$^+$PDGFRa$^+$CD141$^+$ myofibroblasts in the distal regions of human lung tissues during the late-stage.

(N) Time course analysis of *NOTUM*+ myofibroblasts, *SCGB3A2*$^+$ terminal airway cells, and *SFTPC*$^+$ tip and stalk cells from early (11 pcw) to late stages (16, 18, 20, and 22 pcw), by *in situ* HCR. At the early stage (at 11 pcw), the tip epithelium that alveolar differentiation competence is absent, directly

differentiates to *SCGB3A2*$^{+}$ distal airway after a short range of intermediate stalks. At the later stages of development, the *SCGB3A2*$^{+}$ airway cells are rarely observed in the distal lung regions near the distal tip/stalk; e.g. 20 pcw afterward. Instead, from 16 to 22 pcw, the alveolar fated-tip epithelium facing *NOTUM*$^{+}$ myofibroblasts began to produce the *SFTPC*+ fetal AT2 cells along the tip-stalk axis until reaching at the *SCGB3A2*$^{+}$ terminal bronchiole regions. *NOTUM*, red; *SCGB3A2*, green; white, *SFTPC*. DAPI, nuclei. Arrowheads and dashed line indicate the myofibroblasts and stalk epithelial cells, respectively.

(O and P) Time-course visualization (O) and quantitation (P) of *SFTPC*$^{-}$/*SPOCK2*$^{+}$ stalk cells (hereafter, fetal AT1 cells) in the fetal lung tissues at 20 pcw afterward, analysed by *in situ* HCR. The fetal AT1 cells are located at the stalk regions, next to the fetal AT2 cells. *SPOCK2*, red, AT1 cell marker; *SFTPC*, green, AT2 cell marker; white, *NKX2.1*, lung epithelial cell marker. DAPI, nuclei. Arrowheads, fetal AT1 cells. Arrows, fetal AT2 cells. Line, tip epithelium. Dashed line, NKX2.1+ epithelial layer.

DAPI indicates nuclei. Scale bar, 50 µm.

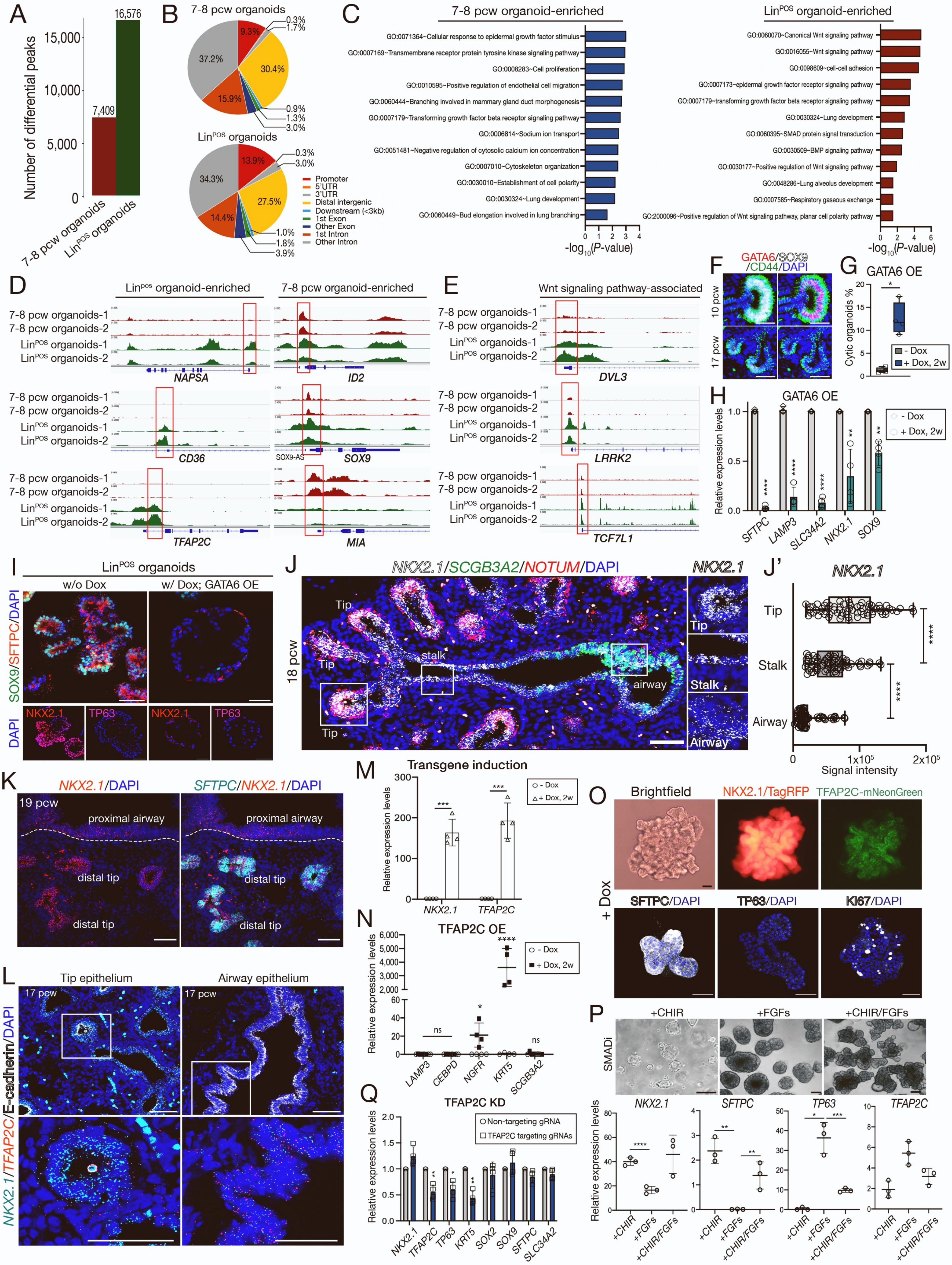
A
Number of differential peaks
16,576
7,409
7-8 pcw organoids
Lin^POS organoids
B
7-8 pcw organoids
Lin^POS organoids
Promoter
5'UTR
3'UTR
Distal intergenic
Downstream (<3kb)
1st Exon
Other Exon
1st Intron
Other Intron
C
7-8 pcw organoid-enriched
GO:0071364~Cellular response to epidermal growth factor stimulus
GO:0007169~Transmembrane receptor protein tyrosine kinase signaling pathway
GO:0008283~Cell proliferation
GO:0010595~Positive regulation of endothelial cell migration
GO:0060444~Branching involved in mammary gland duct morphogenesis
GO:0007179~Transforming growth factor beta receptor signaling pathway
GO:0006814~Sodium ion transport
GO:0051481~Negative regulation of cytosolic calcium ion concentration
GO:0007010~Cytoskeleton organization
GO:0030010~Establishment of cell polarity
GO:0030324~Lung development
GO:0060449~Bud elongation involved in lung branching
-log10(P-value)
Lin^POS organoid-enriched
GO:0060070~Canonical Wnt signaling pathway
GO:0016055~Wnt signaling pathway
GO:0098609~cell-cell adhesion
GO:0007173~epidermal growth factor receptor signaling pathway
GO:0007179~transforming growth factor beta receptor signaling pathway
GO:0030324~Lung development
GO:0060395~SMAD protein signal transduction
GO:0030509~BMP signaling pathway
GO:0030177~Positive regulation of Wnt signaling pathway
GO:0048286~Lung alveolus development
GO:0007585~Respiratory gaseous exchange
GO:2000096~Positive regulation of Wnt signaling pathway, planar cell polarity pathway
-log10(P-value)
D
Lin^pos organoid-enriched
7-8 pcw organoid-enriched
NAPSA
ID2
CD36
SOX9
TFAP2C
MIA
E
Wnt signaling pathway-associated
DVL3
LRRK2
TCF7L1
F
GATA6/SOX9/CD44/DAPI
10 pcw
17 pcw
G
GATA6 OE
Cytic organoids %
- Dox
+ Dox, 2w
H
GATA6 OE
Relative expression levels
SFTPC
LAMP3
SLC34A2
NKX2.1
SOX9
I
Lin^POS organoids
w/o Dox
w/ Dox; GATA6 OE
SOX9/SFTPC/DAPI
NKX2.1
TP63
DAPI
J
NKX2.1/SCGB3A2/NOTUM/DAPI
18 pcw
Tip
stalk
airway
Stalk
Airway
J'
NKX2.1
Signal intensity
K
NKX2.1/DAPI
SFTPC/NKX2.1/DAPI
19 pcw
proximal airway
distal tip
L
Tip epithelium
Airway epithelium
17 pcw
NKX2.1/TFAP2C/E-cadherin/DAPI
M
Transgene induction
Relative expression levels
NKX2.1
TFAP2C
N
TFAP2C OE
LAMP3
CEBPD
NGFR
KRT5
SCGB3A2
O
Brightfield
NKX2.1/TagRFP
TFAP2C-mNeonGreen
+ Dox
SFTPC/DAPI
TP63/DAPI
KI67/DAPI
P
SMADi
+CHIR
+FGFs
+CHIR/FGFs
NKX2.1
SFTPC
TP63
TFAP2C
Q
TFAP2C KD
Non-targeting gRNA
TFAP2C targeting gRNAs
NKX2.1
TFAP2C
TP63
KRT5
SOX2
SOX9
SFTPC
SLC34A2

Figure S6. NKX2.1 drives the onset of the alveolar program whilst supressing the airway program. Related to Figure 5.

(A) Analysis of chromatin accessibility in the 7-8 pcw and Lin^{POS} organoids by bulk-ATAC seq. Approximately 90,000 peaks were found in total, and 7,409 and 16,576 of differentially enriched peaks were identified in between 7-8 pcw organoids and Lin^{POS} organoids, respectively (Table S2; fold change > 2 and FDR< 0.05).

(B) Pie charts representing the genomic distribution of global accessible chromatin regions in the 7-8 pcw organoids and Lin^{POS} organoids.

(C) Biological Process-associated GO term analysis using the differential accessible chromatin regions highly enriched in the 7-8 pcw organoids and Lin^{POS} organoids.

(D and E) IGV image shots of representative ATAC seq tracks at loci showing differentially accessible chromatin regions between the 7-8 pcw organoids and Lin^{POS} organoids. Red box indicates the promoter regions.

(F) Fluorescence imaging of tip epithelium in 10 and 17 pcw lung tissues, by immunostaining. Red, GATA6; white, SOX9; green, CD44.

(G) Proportions of cystic organoids morphologically converted from the folded Lin^{POS} organoids were counted after 2 weeks of GATA6 overexpression in the self-renewal medium. Significance was evaluated by student t-test; *P<0.05.

(H, I) Gene expression (H) and immunofluorescence (I) analysis of the Lin^{POS} organoids overexpressed with GATA6. The qRT-PCR data (H) was normalized to the Lin^{POS} organoids treated without Dox; mean ± SD of biological 4 replicates. Data was normalized to the Lin^{POS} organoids treated with DMSO only; mean ± SD of biological 3 replicates. Significance was evaluated by 1-way ANOVA (H); *P<0.05, **P<0.01, ***P<0.001, ****P<0.0001.

(J) *In situ* HCR analysis of human fetal lung tissues at 18 pcw. *NKX2.1*, white; *NOTUM*, red; *SCGB3A2*, green. Dashed line indicates the boundary of the stalk epithelial tube. (J') Intensity of NKX2.1 signals at tip, stalk, and airway regions were measured using ImageJ. Mean ± SD of 4 biological replicates of late stage lungs at different ages, 15 to 18 pcw. Significance was evaluated by one-way ANOVA; *P<0.05, **P<0.01, ***P<0.001, ****P<0.0001.

(K and L) *In situ* HCR images for detecting transcripts, *SFTPC* or *TFAP2C* with *NKX2.1,* followed by immunostaining (L. E-cadherin), in 17 (L) and 19 pcw (K) fetal lung tissues.

(M) Transgene induction following doxycycline treatment for 2 weeks measured by qRT-PCR. Data was normalized to the untreated group; mean ± SD of four biological replicates. Significance was evaluated by 1-way ANOVA with Tukey multiple comparison post-test; ns: not significant, ****P<0.0001.

(N) qRT-PCR analysis of 7-9 pcw organoids overexpressing TFAP2C for 2 weeks. Data was normalized to EPCAM^{+} positive cells freshly isolated from 20 pcw tip tissues; mean ± SD of

biological 4 replicates. Significance was evaluated by 1-way ANOVA with Tukey multiple comparison post-test; ns: not significant, *$P<0.05$, **$P<0.01$, ***$P<0.001$ and ****$P<0.0001$.

(O) Morphology and fluorescent images of the 7-9 pcw organoids overexpressing both NKX2.1 and TFAP2C for 2 weeks.

(P) qRT-PCR of endogenous *NKX2.1*, *SFTPC*, and *TP63* in the Lin^{POS} organoids cultured in medium containing CHIR, FGF7 or CHIR/FGF7. Data was normalized to EPCAM^{+} cells freshly isolated from 20 pcw tip tissues; mean ± SD of three biological replicates. Significance was evaluated by 1-way ANOVA with Tukey multiple comparison post-test; ns: not significant, *$P<0.05$, **$P<0.01$, ***$P<0.001$, ****$P<0.0001$.

(Q) Knock-down (KD) of endogenous *TFAP2C* in the Lin^{POS} organoids by CRISPR-dCas9-KRAB system. Data was normalized to non-targeting gRNAs; mean ± SD of 3 biological replicates. Significance was evaluated by 1-way ANOVA with Tukey multiple comparison post-test; *$P<0.05$, **$P<0.01$, ***$P<0.001$, ****$P<0.0001$.

DAPI indicates nuclei. Scale bar, 50 µm.

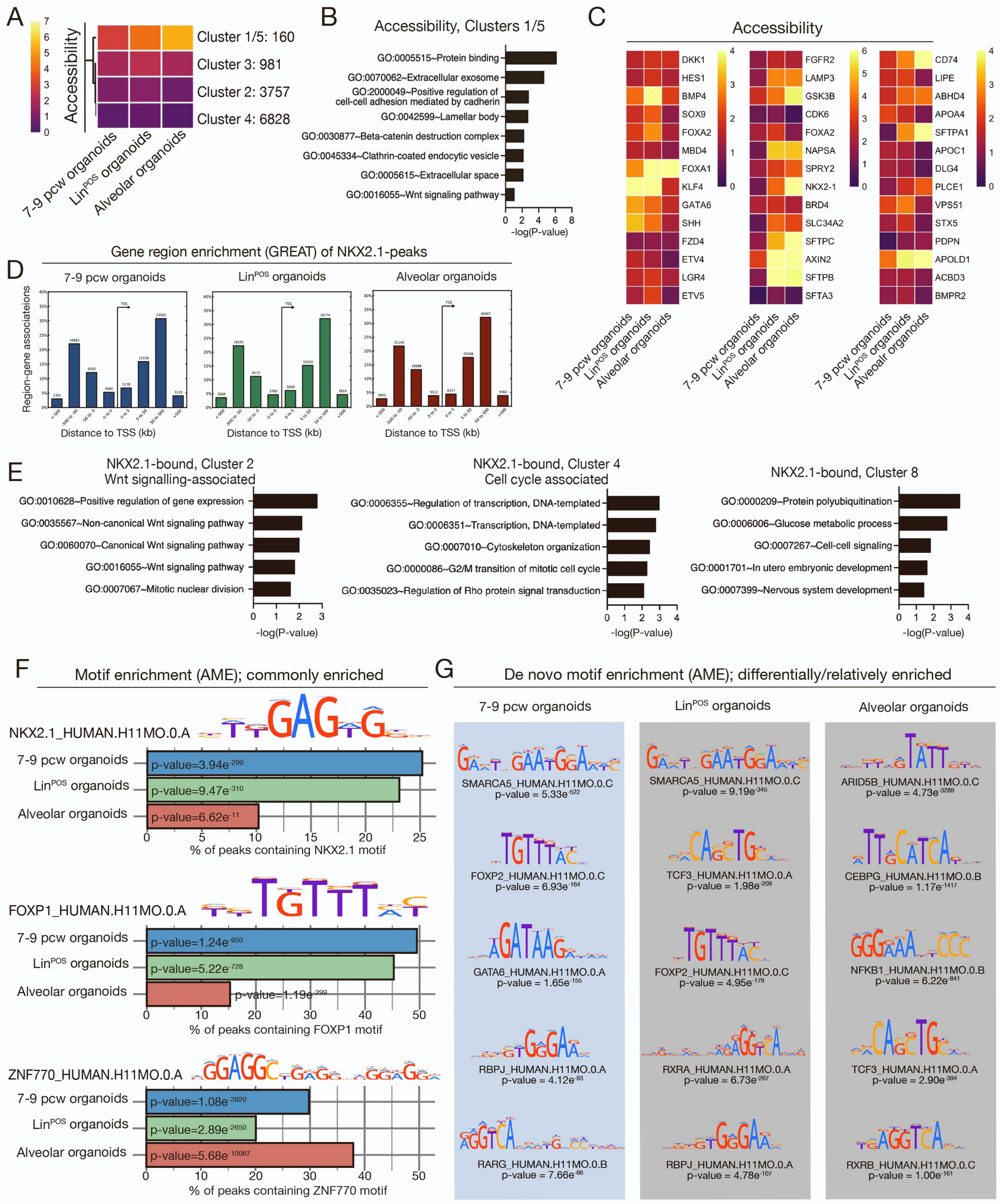

A
Accessibility
Cluster 1/5: 160
Cluster 3: 981
Cluster 2: 3757
Cluster 4: 6828
7-9 pcw organoids
Lin POS organoids
Alveolar organoids
B
Accessibility, Clusters 1/5
GO:0005515~Protein binding
GO:0070062~Extracellular exosome
GO:2000049~Positive regulation of cell-cell adhesion mediated by cadherin
GO:0042599~Lamellar body
GO:0030877~Beta-catenin destruction complex
GO:0045334~Clathrin-coated endocytic vesicle
GO:0005615~Extracellular space
GO:0016055~Wnt signaling pathway
-log(P-value)
C
Accessibility
DKK1
HES1
BMP4
SOX9
FOXA2
MBD4
FOXA1
KLF4
GATA6
SHH
FZD4
ETV4
LGR4
ETV5
FGFR2
LAMP3
GSK3B
CDK6
FOXA2
NAPSA
SPRY2
NKX2-1
BRD4
SLC34A2
SFTPC
AXIN2
SFTPB
SFTA3
CD74
LIPE
ABHD4
APOA4
SFTPA1
APOC1
DLG4
PLCE1
VPS51
STX5
PDPN
APOLD1
ACBD3
BMPR2
Alveoalr organoids
D
Gene region enrichment (GREAT) of NKX2.1-peaks
7-9 pcw organoids
Lin POS organoids
Alveolar organoids
Region-gene associateions
Distance to TSS (kb)
E
NKX2.1-bound, Cluster 2
Wnt signalling-associated
GO:0010628~Positive regulation of gene expression
GO:0035567~Non-canonical Wnt signaling pathway
GO:0060070~Canonical Wnt signaling pathway
GO:0016055~Wnt signaling pathway
GO:0007067~Mitotic nuclear division
NKX2.1-bound, Cluster 4
Cell cycle associated
GO:0006355~Regulation of transcription, DNA-templated
GO:0006351~Transcription, DNA-templated
GO:0007010~Cytoskeleton organization
GO:0000086~G2/M transition of mitotic cell cycle
GO:0035023~Regulation of Rho protein signal transduction
NKX2.1-bound, Cluster 8
GO:0000209~Protein polyubiquitination
GO:0006006~Glucose metabolic process
GO:0007267~Cell-cell signaling
GO:0001701~In utero embryonic development
GO:0007399~Nervous system development
-log(P-value)
F
Motif enrichment (AME); commonly enriched
NKX2.1_HUMAN.H11MO.0.A
p-value=3.94e-299
p-value=9.47e-310
p-value=6.62e-11
% of peaks containing NKX2.1 motif
FOXP1_HUMAN.H11MO.0.A
p-value=1.24e-950
p-value=5.22e-728
p-value=1.19e-299
% of peaks containing FOXP1 motif
ZNF770_HUMAN.H11MO.0.A
p-value=1.08e-3920
p-value=2.89e-2650
p-value=5.68e-10087
% of peaks containing ZNF770 motif
G
De novo motif enrichment (AME); differentially/relatively enriched
7-9 pcw organoids
Lin POS organoids
Alveolar organoids
SMARCA5_HUMAN.H11MO.0.C
p-value = 5.33e-522
FOXP2_HUMAN.H11MO.0.C
p-value = 6.93e-164
GATA6_HUMAN.H11MO.0.A
p-value = 1.65e-155
RBPJ_HUMAN.H11MO.0.A
p-value = 4.12e-93
RARG_HUMAN.H11MO.0.B
p-value = 7.66e-66
SMARCA5_HUMAN.H11MO.0.C
p-value = 9.19e-345
TCF3_HUMAN.H11MO.0.A
p-value = 1.98e-209
FOXP2_HUMAN.H11MO.0.C
p-value = 4.95e-179
RXRA_HUMAN.H11MO.0.A
p-value = 6.73e-267
RBPJ_HUMAN.H11MO.0.A
p-value = 4.78e-107
ARID5B_HUMAN.H11MO.0.C
p-value = 4.73e-3289
CEBPG_HUMAN.H11MO.0.B
p-value = 1.17e-1417
NFKB1_HUMAN.H11MO.0.B
p-value = 6.22e-841
TCF3_HUMAN.H11MO.0.A
p-value = 2.90e-394
RXRB_HUMAN.H11MO.0.C
p-value = 1.00e-161

Figure S7. NKX2.1-DamID-seq analysis of lung tip organoids and alveolar organoids. Related to Figure 6.

(A) Heatmap illustrating k-means clustering of chromatin accessibility across the organoid samples (Dam-only). Colours represent binding intensities from genes associated with peaks, that was averaged across gene bodies, including -1kb from the TSS.

(B) GO enrichment analysis of BP-associated GO terms on the Clusters 1 and 5 showing increased binding intensity across the organoid samples.

(C) Heatmap describing the representative genes that showed relatively high chromatin accessibility for each sample.

(D) Distribution across genomic features analysed using GREAT.

(E) GO enrichment analysis of BP-associated GO terms on the Clusters 2 (Wnt signalling-associated), 4 (cell cycle-associated), and 6.

(F and G) Motif enrichment analysis performed using AME package from MEME suite. Commonly enriched (F) and differentially/relatively enriched (G) motifs were selected with *p*-value across the organoid samples.
