# Supplementary material for: Organoid modeling of human fetal lung alveolar development reveals mechanisms of cell fate patterning and neonatal respiratory disease
Source: Cell Stem Cell. Author manuscript; Available in PMC 2025 Dec 9. (PMC7618456; doi:10.1016/j.stem.2022.11.013)
Supplement: Supplementary Material [file EMS211309-supplement-Supplementary_Material.zip › 1-s2.0-S193459092200460X-mmc9.pdf]

Cell Stem Cell

Article

Organoid modeling of human fetal lung alveolar development reveals mechanisms of cell fate patterning and neonatal respiratory disease

Graphical abstract

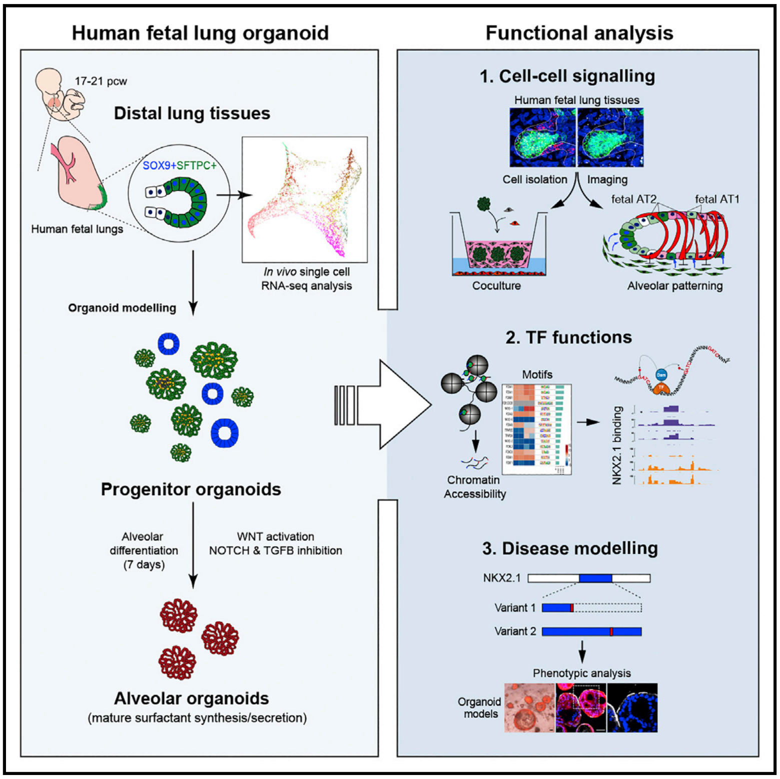

Highlights

- Human fetal lung tip progenitor cells commit to alveolar fate from 15 pcw *in vivo*
- Late-stage tip-derived organoids readily differentiated to mature cell fates
- NOTUM^{+} myofibroblasts spatially pattern differentiating human alveolar-type-2 cells
- Lineage-determining TFs facilitate organoid modeling of neonatal lung disease

Authors

Kyungtae Lim, Alex P.A. Donovan, Walfred Tang, ..., Kerstin B. Meyer, Andrea H. Brand, Emma L. Rawlins

Correspondence

elr21@cam.ac.uk

In brief

Lim and colleagues identify the timing of alveolar-fate specification in human fetal lungs. Their stage-specific organoids allow functional analysis of mechanisms controlling fate maintenance and differentiation in the developing alveolar niche, facilitating the modeling of neonatal lung disease.

Check for updates

Lim et al., 2023, Cell Stem Cell *30*, 20–37
January 5, 2023 © 2022 The Author(s). Published by Elsevier Inc.
https://doi.org/10.1016/j.stem.2022.11.013

CellPress

CellPress
OPEN ACCESS

Cell Stem Cell

Article

Organoid modeling of human fetal lung alveolar development reveals mechanisms of cell fate patterning and neonatal respiratory disease

Kyungtae Lim,[1,2,3] Alex P.A. Donovan,[1,2] Walfred Tang,[1,2,3] Dawei Sun,[1,2,3,6] Peng He,[4,5] J. Patrick Pett,[4] Sarah A. Teichmann,[4] John C. Marioni,[4,5] Kerstin B. Meyer,[4] Andrea H. Brand,[1,2] and Emma L. Rawlins[1,2,3,7,*]
[1]Wellcome Trust, CRUK Gurdon Institute, University of Cambridge, Cambridge CB2 1QN, UK
[2]Department of Physiology, Development and Neuroscience, University of Cambridge, Cambridge CB2 3DY, UK
[3]Wellcome Trust, MRC Stem Cell Institute, Jeffrey Cheah Biomedical Centre Cambridge Biomedical Campus, Puddicombe Way, Cambridge CB2 0AW, UK
[4]Wellcome Sanger Institute, Hinxton, Cambridge CB10 1SA, UK
[5]European Molecular Biology Laboratory, European Bioinformatics Institute (EMBL-EBI), Wellcome Genome Campus, Cambridge CB10 1SD, UK
[6]Present address: Broad Institute of Massachusetts Institute of Technology and Harvard, Cambridge, MA 02142, USA
[7]Lead contact
*Correspondence: elr21@cam.ac.uk
https://doi.org/10.1016/j.stem.2022.11.013

SUMMARY

Variation in lung alveolar development is strongly linked to disease susceptibility. However, underlying cellular and molecular mechanisms are difficult to study in humans. We have identified an alveolar-fated epithelial progenitor in human fetal lungs, which we grow as self-organizing organoids that model key aspects of cell lineage commitment. Using this system, we have functionally validated cell-cell interactions in the developing human alveolar niche, showing that Wnt signaling from differentiating fibroblasts promotes alveolar-type-2 cell identity, whereas myofibroblasts secrete the Wnt inhibitor, NOTUM, providing spatial patterning. We identify a Wnt-NKX2.1 axis controlling alveolar differentiation. Moreover, we show that differential binding of NKX2.1 coordinates alveolar maturation, allowing us to model the effects of human genetic variation in *NKX2.1* on alveolar differentiation. Our organoid system recapitulates key aspects of human fetal lung stem cell biology allowing mechanistic experiments to determine the cellular and molecular regulation of human development and disease.

INTRODUCTION

During human lung development the airway tree is formed by branching between ~5 and 16 post-conception weeks (pcw). From ~16 to 26 pcw, the most distal epithelial tubes narrow, come into proximity to capillaries, and start to differentiate as alveolar epithelium.[1,2] Preterm infants born at more than ~22 pcw have a rudimentary gas exchange surface and can survive if provided with specialized intensive care. However, the molecular mechanisms underlying human alveolar development remain largely unknown.

The human tip epithelium is SOX9/SOX2 dual-positive during airway branching, functions as a multipotent progenitor and has been cultured as organoids.[3,4] From ~16 pcw, as alveolar differentiation begins, tip progenitors become SOX9 single-positive and more cuboidal in shape.[3] We hypothesized that growth of tip organoids from 16 to 22 pcw lungs would provide an improved model for studying human alveolar differentiation. In addition, human embryonic lung organoids are amenable to viral transduction and gene editing,[5] and we speculated that we would be able to obtain genetically manipulable mature human alveolar epithelial cells from such organoids.

Differentiation of human iPSCs to alveolar lineages suggests that Wnt signaling is essential for alveolar fate.[6] However, Wnt ligands are expressed widely in developing human lungs,[7] and how spatial patterning of cell fates is achieved is unknown. In mouse lungs, Nkx2.1 is essential for alveolar differentiation and maintenance and binds to promoters of alveolar type 1 (AT1)- and alveolar type 2 (AT2)-cell-specific genes.[8] Heterozygous missense mutations in the *NKX2.1* homeodomain cause brain-lung-thyroid syndrome, which includes disrupted surfactant gene expression and interstitial lung disease.[9,10] However, whether NKX2.1 simply promotes surfactant synthesis,[10] or has additional roles in human lung alveolar differentiation is unknown.

We find that SOX9^{+} human lung tip progenitors acquire an AT2 gene expression signature by 15 pcw and can grow as self-renewing (SN) organoids that can be readily differentiated. This has allowed us to determine upstream signals and downstream transcription factors (TFs) that promote lineage commitment, identify the role of myofibroblasts in spatial patterning of the developing alveolus and to build models of human neonatal lung disease. This work also provides a source of genetically manipulable human AT2 cells for disease modeling.

 © 2022 The Author(s). Published by Elsevier Inc.
This is an open access article under the CC BY license (http://creativecommons.org/licenses/by/4.0/).

CellPress
OPEN ACCESS

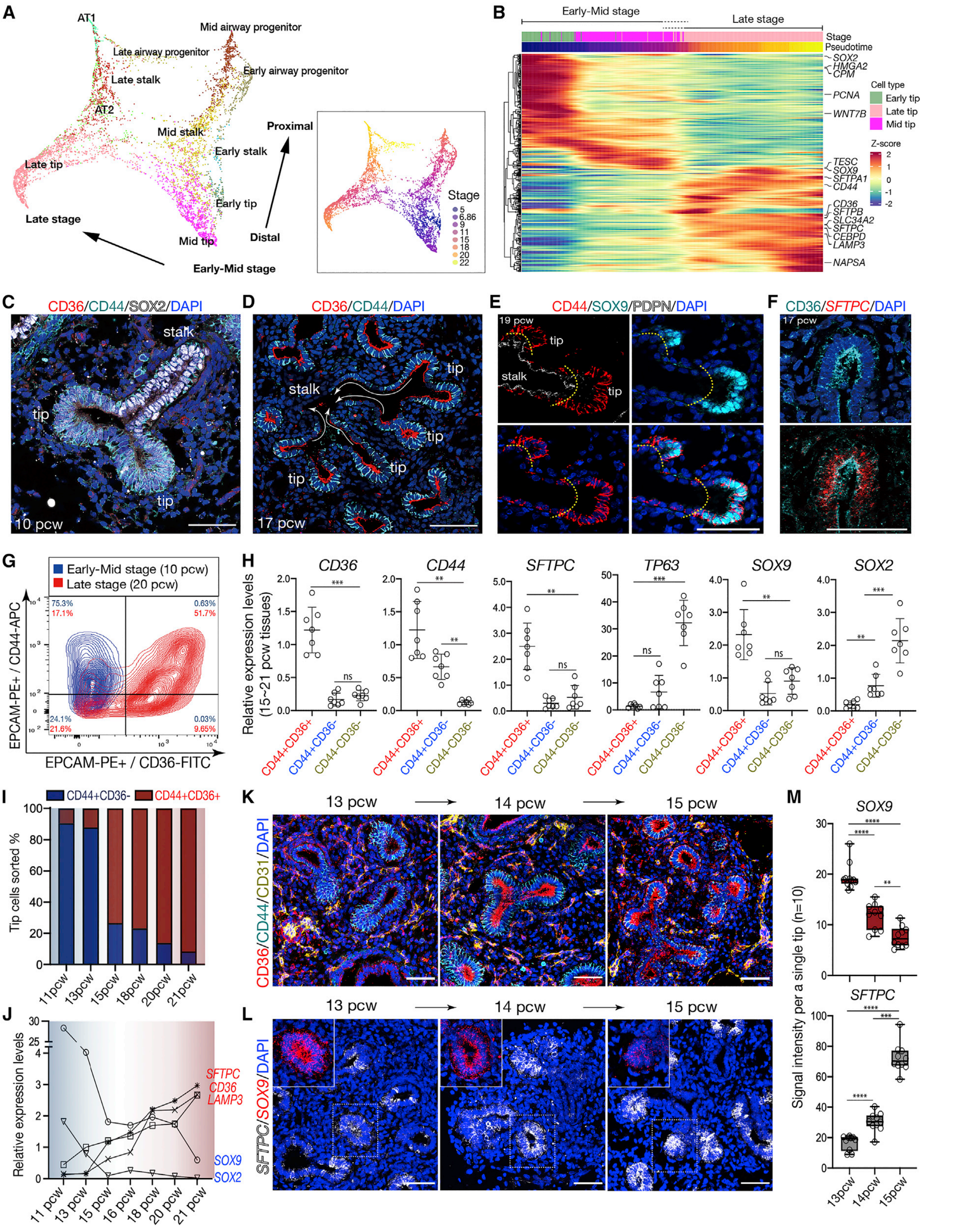

(legend on next page)

RESULTS

Human fetal lung tip progenitor cells intrinsically activate alveolar lineage markers from 15 pcw *in vivo*

We investigated distal regions of human fetal lungs by single-cell RNA sequencing (scRNA-seq)[7] (Figure 1A). 11 epithelial cell clusters were identified, including tip, stalk, airway progenitors, AT2, and AT1 cells, each with distinct markers (Figures 1A, S1A, and S1B). Force-directed embedding showed that the clusters were largely grouped into two distinct regions by stage: 5–11 pcw (“early-mid”), and 15–22 pcw (“late”) (Figure 1A). Along the distal to proximal axis of the plot, the early- and mid-stage tips were transcriptionally connected to stalk cells, followed by airway progenitors. Whereas late-stage tips were closely linked to stalk and airway progenitors and to AT2 and AT1 cells (Figure 1A), suggesting late-stage tip cells gain alveolar differentiation competence. Differential gene expression analysis showed that late-stage tip cells co-expressed alveolar lineage markers, such as *SFTPC, LAMP3*, and *SLC34A2*, with tip progenitor markers (Figures 1B, S1A, and S1B). Analysis of tissue sections confirmed that 10–12 pcw distal tips were columnar and marked by SOX9, SOX2, and *TPPP3*. 17–22 pcw distal tip epithelium contained more cuboidal $SOX2^-$, $SOX9^+$, $TPPP3^+$ cells, which co-expressed AT2 cell markers, SFTPC, and HTII-280 (Figures S1C–S1E). Fetal AT2 cells were located outside of the tip region and did not co-express progenitor markers (Figure S1E). We identified surface markers to distinguish between early-mid (airway)- and late (alveolar)-stage tips (Figures 1B and S1B). CD44 marks tip epithelial cells across all stages of lung development tested. Whereas CD36 is specific to the ~15–22 pcw stage tips where it is co-expressed with CD44, *SFTPC* and SOX9. A lower level of both CD44 and CD36 extends into the $SOX9^-$/$PDPN^+$ tip-adjacent (stalk) cells, and CD44 extends further proximally into the differentiating stalk (Figures 1C–1F, S1F, and S1G).

Distal lung regions were dissected to enrich for the tip, and $EPCAM^+$ cells were sorted for CD44 and/or CD36. At 10 pcw, 75% of sorted cells were $CD44^+$, and $CD44^+CD36^+$ cells were rare (Figures 1G and S1H). By contrast, at 20 pcw 53% of sorted cells were $CD44^+CD36^+$ and only 17% were single $CD44^+$ (Figures 1G and S1H). qRT-PCR showed that the 17–20 pcw $CD44^+CD36^+$ cells robustly expressed *CD36*, *CD44*, *SFTPC*, and *SOX9*, but extremely low levels of the airway markers, *TP63* and *SOX2*, consistent with immunostaining (Figure 1H). In contrast, single $CD44^+$ cells showed a higher level of *SOX2*, but much lower levels of *SFTPC* and *SOX9*, suggesting they are derived from the $CD44^+SOX9^-$ stalk region (Figures 1H and S1G). Finally, the $CD44^-CD36^-$ cells had higher levels of TP63 and *SOX2*, but low *SFTPC* and *SOX9*, indicating that they are derived from more proximal airway-lineage cells (Figure 1H). Therefore, dual expression of CD44 and CD36 marks the tip epithelial population in the 15–21 pcw stage lung, and this population co-expresses tip progenitor and alveolar markers.

Gradual acquisition of tip alveolar lineage signature during human lung development

Flow cytometric analysis showed that the expression of CD36 was robustly acquired between 13 and 15 pcw, prior to morphological alveolar differentiation (Figure 1I). Similarly, in the $CD36^+$ cells, *SFTPC*, *CD36*, and *LAMP3* mRNA began to increase from 13 pcw (Figure 1J). We confirmed that CD36 was detectable in the tip epithelium at 14 pcw, moreover the intensity of *SFTPC* transcripts increased while *SOX9* gradually lowered during this transition period (Figures 1K–1M). Showing that the acquisition of AT2 lineage signatures occurs gradually in the tip, prior to the differentiation of the alveolar epithelium.

Organoids derived from late-stage distal tip epithelium exhibit alveolar lineage signatures

To determine the fate potential of the 17–21 pcw distal tip, $CD44^+CD36^+$ cells were cultured for 3 weeks (Figure 2A) in our established self-renewing (SN) medium.[2] Two morphologically distinct organoids formed: cystic and folded (Figure 2B). Folded organoids consisted of cuboidal cells and expressed both progenitor and AT2 markers, including an *SFTPC*-eGFP reporter (Figure 2C). By contrast, cystic organoids had columnar cell shape and expressed tip progenitor, but not AT2, markers resembling the airway branching stage (early/mid) tips (Figures 2B–2E, S2A, and S2B). We refer to the folded and cystic organoids isolated from 17 to 21 pcw lungs as lineage positive (Lin^{POS}) and negative (Lin^{NEG}).

Figure 1. Human fetal lung tip progenitor cells acquire alveolar features during normal development

(A) Force-directed embedding of single-cell transcriptomes of cells derived from distal human lung tissues from 5 to 22 pcw, by cell types and stages.
(B) Trajectory heatmap showing differential marker gene expression among early, mid, and late-stage tip cells.
(C–F) Surface antigens, CD44 and CD36, mark tip epithelium at early-mid and late stages. Lungs at 10 (C), 17 (D and F), and 19 pcw (E) were stained with CD36 and CD44 and/or SOX2 and SOX9 antibodies. Arrows (D) show patterning from distal to proximal regions. Yellow dashed lines (E) indicate separation of $SOX9^+$ tip regions from the $PDPN^+$ stalk. The *SFTPC* transcript was visualized by *in situ* hybridization chain reaction (HCR) (F) following immunostaining for CD36.
(G) Flow cytometry of the human lung tip epithelial population at 10 (blue) and 20 pcw (red).
(H) qRT-PCR of the freshly purified lung epithelial cells sorted from the late-stage lungs. Data normalized to fresh $EPCAM^+$ cells from 20 pcw distal tissues; mean ± SD, n = 7 (15–21 pcw). Significance evaluated by one-way ANOVA with Tukey multiple comparison post-test; ns: not significant, *p < 0.05, **p < 0.01, *** p < 0.001.
(I) Proportion of the freshly purified tip epithelium as $CD44^+CD36^-$ or $CD44^+CD36^+$ at 11, 13, 15, 18, 20, and 21 pcw; n = 1 each time.
(J) Relative mRNA levels of the tip progenitor markers, *SOX9* and *SOX2*, and type 2 alveolar lineage markers, *SFTPC*, *CD36*, and *LAMP3*, in $CD44^+CD36^-$ tip epithelial population at 11 and 13 pcw, and in $CD44^+CD36^+$ tip epithelial population at 15, 16, 18, 20, and 21 pcw, by qRT-PCR. Data were normalized to fresh $EPCAM^+$ cells from 20 pcw tip tissues; n = 1 at each stage.
(K and L) Human fetal lung tissues during the transition from 13 to 15 pcw were stained using antibodies against CD36, CD44, and CD31 (K), or for *SFTPC* and *SOX9* mRNA (L). Three 13 pcw, two 14 pcw, and two 15 pcw samples.
(M) Signal intensity of *SOX9* and *SFTPC* transcripts in Figure 1L. Ten tip regions analyzed per stage, and the intensity represented as mean ± SD. Significance evaluated by one-way ANOVA with Tukey multiple comparison post-test; ns: not significant, *p < 0.05, **p < 0.01, ***p < 0.001, ****p < 0.001.
DAPI, nuclei. Scale bars, 50 μm.
See also Figure S1.

CellPress
OPEN ACCESS

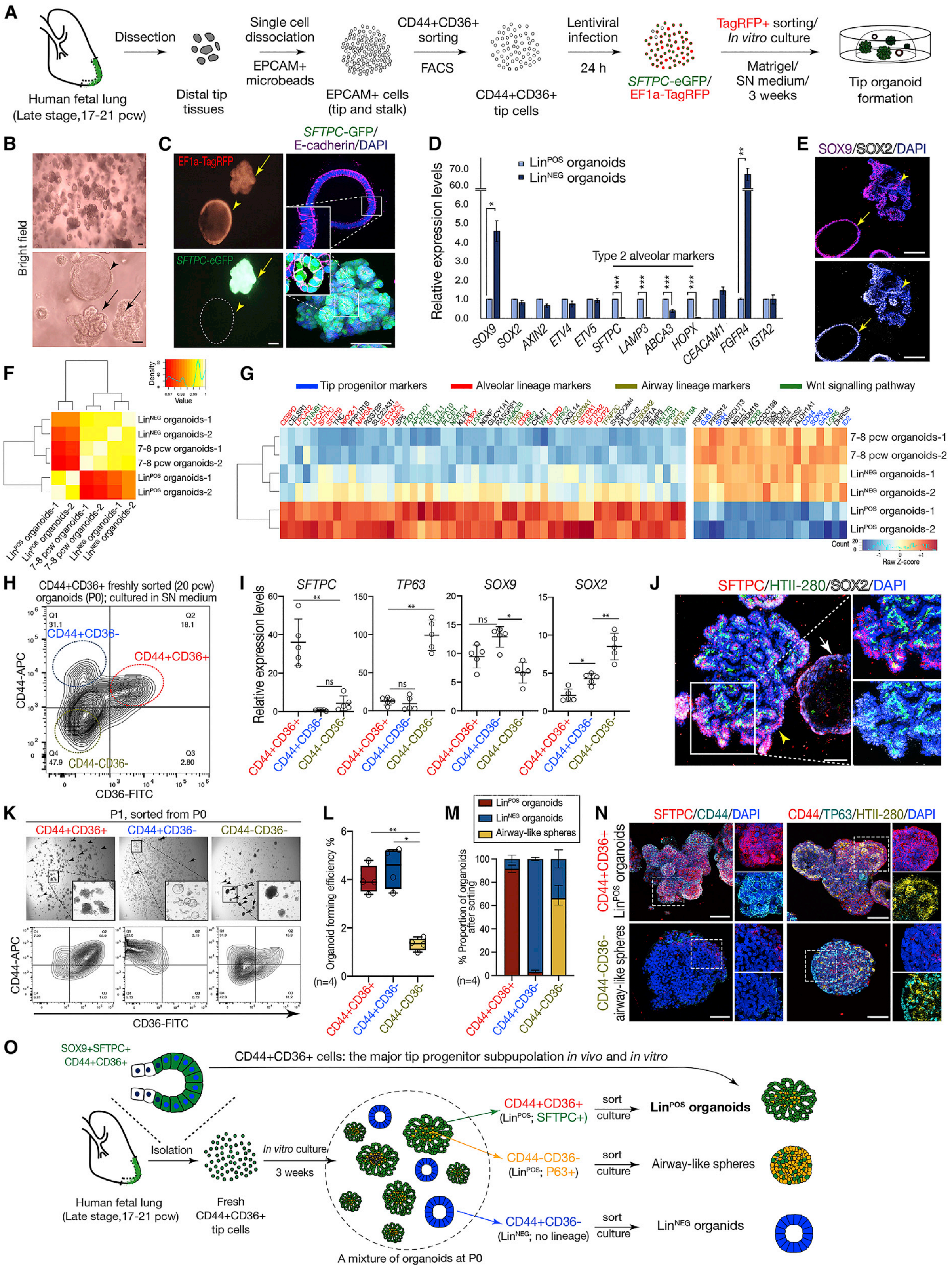

(legend on next page)

We performed RNA-seq to compare the transcriptome of passaged early-mid stage tip organoids derived from 7 to 8 pcw with passaged 17–21 pcw Lin^{NEG} and Lin^{POS} organoids (Figure S2C). Hierarchical clustering and principal component analysis showed that the Lin^{NEG} organoids were very similar to the 7–8 pcw (early-mid tip) organoids, but distinct from the Lin^{POS} organoids (Figures 2F and S2D). We identified >280 differentially expressed genes between the Lin^{POS} and Lin^{NEG} organoids (Table S1; Figure S2E; $log_2FC > 4$, $p < 0.05$). Similar to the 7–8 pcw organoids, the Lin^{NEG} organoids were highly associated with gene ontology (GO) terms related to ion transport and branching morphogenesis, confirming that they resemble the early-mid distal tips. Whereas the Lin^{POS} organoids had significant GO terms for respiratory gaseous exchange and lung alveolus development, as well as canonical Wnt pathway signaling (Figures S2F–S2I). Moreover, Lin^{POS} organoids were enriched for AT2 markers, Wnt signaling-related genes and low levels of airway genes (Figure 2G). These data confirm that the passaged Lin^{POS} organoids recapitulate key molecular characteristics of the alveolar/late-stage lung tip progenitors.

Late-tip epithelial cells function as multipotent stem cells *in vitro*

We tested whether any organoids from the 17 to 21 pcw mixed Lin^{POS} and Lin^{NEG} population retained CD44 and CD36 expression after 3 weeks culture (Figure 2H). The CD44$^+$CD36$^+$ cells showed the highest level of *SFTPC* with a moderate level of *SOX9*, but very low levels of *SOX2* and *TP63*. They were located at the tips of the Lin^{POS} organoids where they expressed SFTPC, HTII-280, SOX9, PORCN, CD44, and KI67 (Figures 2I, 2J, and S2J–S2N). The CD44$^-$CD36$^-$ cells had the highest levels of airway markers *TP63* and *SOX2*. They corresponded to the inner parts of the Lin^{POS} organoids where scattered TP63$^+$ cells were found (Figures 2I, 2J, and S2K). By contrast, the CD44$^+$CD36$^-$ cells had the highest level of *SOX9* and a moderate level of *SOX2*, but no lineage markers, and corresponded to the Lin^{NEG} organoids which had uniform CD44, SOX2, and SOX9 (Figures 2I, 2J, and S2M). These data suggested that the CD44$^+$CD36$^+$ late-stage tip cells originally plated had self-renewed (at the tips) and differentiated toward airway lineages (in the center) to form the Lin^{POS} organoids.

We tested whether the CD44$^+$CD36$^+$ cells continued to self-renew upon passaging. P0 organoids (mixed population of Lin^{POS} and Lin^{NEG}) were sorted as CD44$^+$CD36$^+$, CD44$^+$CD36$^-$ and CD44$^-$CD36$^-$ and cultured separately in the SN medium. Only the CD44$^+$CD36$^+$ cells were able to generate a large proportion of Lin^{POS} organoids with folded structure and progenitor/AT2 gene signature (Figures 2K–2O and S2O). In contrast, the CD44$^+$CD36$^-$ cells, derived from Lin^{NEG} organoids, produced Lin^{NEG} organoids. The CD44$^-$CD36$^-$ cells, derived from the center of the Lin^{POS} organoids, largely formed airway-fated spheres expressing a significantly higher level of *TP63*/TP63 (Figures 2K–2O and S2O). We confirmed that the CD44$^+$CD36$^+$-cell-derived Lin^{POS} organoids self-renew and maintain their cellular organization for multiple passages (Figures S2P and S2Q). These data demonstrate that the CD44$^+$CD36$^+$ cells are the major progenitor subpopulation *in vitro* and can maintain Lin^{POS} organoids. We have therefore captured the alveolar stage lung tip epithelial population that co-expresses SOX9 and AT2 markers in the Lin^{POS} organoids.

Cultured 17–21 pcw tip cells differentiate readily into alveolar-type-2 cells

Our scRNA-seq data (Figures 1A and 1B) suggested that the late-stage tip cells gain alveolar differentiation competence. We therefore tested whether the Lin^{POS} organoids could differentiate into AT2 cells. In medium containing DAPT (γ-secretase inhibitor; Notch inhibition), DCI (dexamethasone, cyclic AMP [cAMP], and 3-Isobutyl-1-methylxanthine [IBMX]), CHIR (CHIR99021; Wnt agonist) and SB431542 (TGF-β inhibition), *NKX2-1*, *SFTPC*, and *ACE2* were upregulated and *SOX9, SOX2*, and *TP63* downregulated (Figures 3A and S3A–S3C).

Figure 2. CD36, CD44 dual-positive tip cells self-renew and undergo lineage commitment *in vitro* to form late-stage lung organoids
(A) Isolation and viral infection of CD44$^+$CD36$^+$ tip epithelial cells from human fetal lungs at 17–21 pcw, late stage, and *in vitro* culture in self-renewing (SN) medium.
(B and C) Gross morphology (B) of the cultured epithelial tip organoids. Detailed morphology (C) E-cadherin (magenta); *SFTPC*-GFP and *TagRFP*. Arrows and arrowhead indicate folded Lin^{POS} organoids and cystic Lin^{NEG} organoids, respectively. Scale bars, 100 μm.
(D) Gene expression profile of the Lin^{POS} and Lin^{NEG} organoids. Data are quantified by qRT-PCR; mean ± SD of 4 biological replicates. Significance evaluated by unpaired Student's t test; *$p < 0.05$, **$p < 0.01$, ***$p < 0.001$.
(E) Immunofluorescence analysis of the Lin^{POS} (arrowheads) and Lin^{NEG} organoids (arrow) at passage 1 cultured in the SN medium, showing co-expression of SOX9 and SOX2. DAPI, nuclei. Scale bars, 50 μm.
(F) Hierarchical clustering analysis of bulk-RNA-seq data using 7–8 pcw, Lin^{POS} and Lin^{NEG} organoids.
(G) Heatmap analysis of selected genes highly enriched in the 7–8 pcw, Lin^{NEG} and Lin^{POS} organoids.
(H and I) Late-stage lung tip organoids sorted into 3 populations at passage zero by FACS using antibodies against CD36 and CD44 (H). The sorted P0 cell populations were analyzed by qRT-PCR (I). Data were normalized to total EPCAM$^+$ cells freshly sorted from 20 pcw tissues; mean ± SD (n = 5). Significance was evaluated by one-way ANOVA with Tukey multiple comparison post-test; *$p < 0.05$, **$p < 0.01$.
(J) Late-stage tip organoids at passage zero cultured in self-renewal medium stained with SFTPC, HTII-280 and SOX2 antibodies. Arrowheads indicate the tip-like, SFTPC$^+$ subpopulation in the Lin^{POS} organoids. Arrows indicate the Lin^{NEG} organoids.
(K–N) Passage 1 organoids were grown from the sorted CD44$^+$CD36$^+$, CD44$^+$CD36$^-$, or CD44$^-$CD36$^-$ populations at passage 0 and reanalyzed for CD44 and CD36 at the end of passage 1 (K; Figure 2O). Arrows and arrowheads indicate the Lin^{POS} and Lin^{NEG} organoids. The organoid forming efficiency (L), the proportion (M), and the fluorescence images (N) of the organoids of each morphological sub-type at passage 1 was measured at 3 weeks after plating. Data were represented as mean ± SD of 4 biological replicates.
(O) Diagram summarizing the organoid experiments performed. CD44$^+$CD36$^+$ cells from late-stage lung tissues are the major tip progenitor subpopulation *in vitro*, growing into self-renewing Lin^{POS} organoids showing key features of the late-stage lung tip cells.
Scale bars, 100 μm.
See also Figure S2 and Table S1.

CellPress
OPEN ACCESS

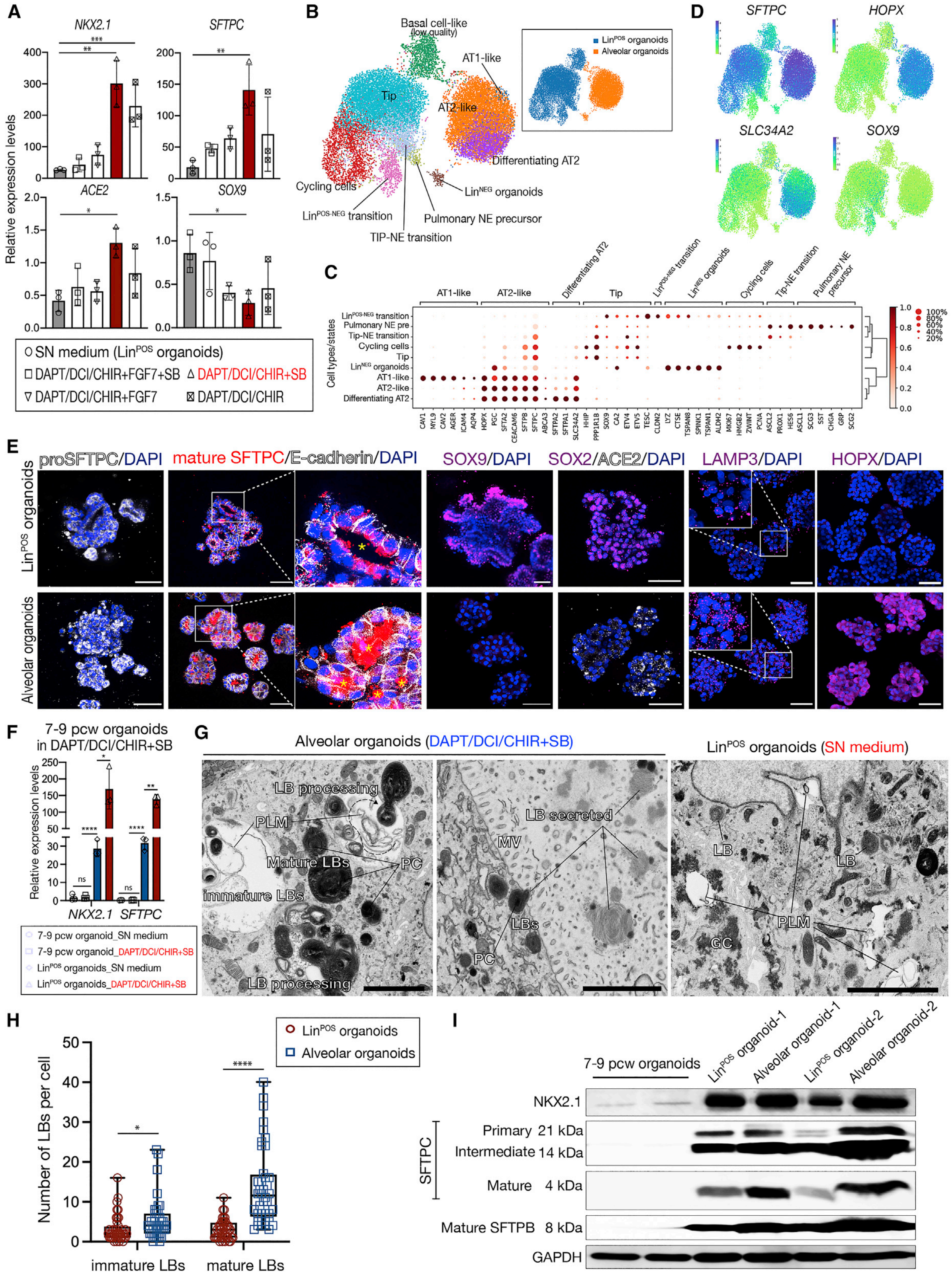

(legend on next page)

Similarly, the *SFTPC*-GFP reporter and the AT2-specific LAMP3, HOPX, and ACE2 proteins increased[11] (Figures 3A, 3E, S3B, and S3C). Withdrawal of DAPT, DCI, or SB431542 caused reduction of mature SFTPC and AT2 gene expression (Figures S3D–S3F). scRNA-seq showed that Lin^{POS} organoids mostly comprised tip and cycling tip cells with small subpopulations of pulmonary neuroendocrine (NE) precursors, basal-like cells, and transitional cell states (Figures 3B–3D). Whereas, differentiated organoids comprised AT2-like cells, with a small number of AT1-like cells (Figures 3C, 3D, and S3G). The medium condition efficiently differentiates the Lin^{POS} organoids to alveolar lineages.

We directly compared the ability of 7–9 pcw, and 17–21 pcw Lin^{NEG} and Lin^{POS} organoids to differentiate to alveolar fates (Figure 3F). This confirmed that 17–21 pcw tips (Lin^{POS} organoids) are in a distinct differentiation-ready state, confirming that cell-intrinsic changes occur in tip progenitors during development.

Electron microscopy revealed that the Lin^{POS} organoids in the SN medium contained rare, immature lamellar bodies (LBs) usually surrounded by glycogen (GC) (Figures 3G and 3H). Higher numbers of LBs with a characteristic surfactant projection core (PC)[12,13] were produced and secreted in the differentiated organoids (Figures 3E, 3G, and 3H). NKX2.1 protein, pro-SFTPC and SFTPB processing (Figure 3I), and SFTPC secretion (Figure 3E)[13] were increased following AT2 differentiation. These data demonstrate that the Lin^{POS} organoids are readily differentiated to a mature AT2 fate and suggest that NKX2.1 levels are important in this process.

Coordinated control of the late-stage tip epithelial cell fate by Wnt and FGF signaling

To determine which factors in SN medium are most important for promoting airway versus alveolar fate of the late-stage tips, freshly isolated tips from 17 to 21 pcw lungs were directly exposed to pairwise signal combinations (Figures 4A, S4A, and S4B). The cells did not grow in the absence of SMADi (SMAD inhibitors; Noggin and SB431542) (Figures S4B and S4C). However, we observed that two distinct populations of organoids were obtained by combining SMADi with CHIR, or with FGFs (FGF7 and FGF10) (Figure S4B). Organoids grown in SMADi/CHIR had a thin epithelium and hollow lumen. They could not be passaged and expressed high levels of the AT2 marker *SFTPC*, greater than the Lin^{POS} organoids. In contrast, organoids grown in SMADi/FGF formed spheres with a small lumen, a thicker proliferative epithelium, and expressed the highest level of the basal cell marker TP63 (Figures 4A, S4B, and S4D). In both conditions *SOX9* was lower than the Lin^{POS} and Lin^{NEG} organoids (Figure S4D). These data indicate that Wnt and FGF signaling promote the lineage determination of the 17–21 pcw tip epithelium to alveolar or airway lineages; in agreement with previous data for Wnt.[6,14] Moreover, when SMADi/CHIR/FGFs were combined (equivalent to our SN medium) organoids displayed a mixture of alveolar and airway characteristics, as in the Lin^{POS} organoids (Figure 4A).

We demonstrated that the 17–21 pcw tips are highly plastic and can switch readily between alveolar and airway differentiation by altering the medium and observing rapid organoid morphology and gene expression changes (Figures S4E–S4G). Freshly isolated 8 pcw distal tip epithelial cells did not show similar levels of differentiation when grown in the same conditions (Figure S4H). These data again indicate that the 17–21 pcw tips are intrinsically different to the airway-stage (8 pcw) tips, being both highly plastic and in a differentiation-ready state.

NOTUM-expressing myofibroblasts pattern the epithelial Wnt response during alveolar formation

We reasoned that Wnt and FGF signaling also control lineage determination of the late-tip epithelium *in vivo* and investigated the role of Wnt. In 17–21 pcw tissue, the *SFTPC*$^+$ tips expressed higher levels of the Wnt targets *AXIN2, WIF1*, and PORCN, compared with stalk and airway epithelium (Figures S4I–S4N and S5A–S5D). Moreover, we confirmed our single-cell data showing that *WNT2* is co-expressed with *FGFR4* in alveolar fibroblasts throughout these stages[7,11] (Figure 4B). This led us to question how Wnt-responsive *SFTPC* could be precisely restricted to the tip epithelium in the presence of widespread *WNT2*. A secreted Wnt inhibitor, *NOTUM*,[15] is expressed in the distal tip epithelium and the myofibroblasts that surround the differentiating stalk cells (Figures 4C, 4C′, and S5B–S5E). The *NOTUM*$^+$ myofibroblasts co-express the Wnt targets *LEF1* and *AXIN2*, suggesting that they also respond to Wnt (Figures S5C and S5D). We hypothesized that in response to WNT2 the myofibroblasts locally secrete NOTUM,

Figure 3. Efficient *in vitro* differentiation of Lin^{POS} organoids to alveolar cell fate

(A) qRT-PCR of the Lin^{POS} organoids cultured in SN medium or alveolar-induction culture conditions containing combinations of DAPT, dexamethasone/cyclic AMP/IBMX (DCI), CHIR with/without SB431542 (SB) and FGF7, for 1 week. *NKX2.1*, *SFTPC*, and *SOX9* levels were normalized to EPCAM$^+$ cells, and *ACE2* was normalized to EPCAM$^-$ cells, freshly sorted from 20 pcw tip tissues; mean ± SD of four biological replicates. Significance was evaluated by one-way ANOVA with Dunnett multiple comparison post-test; *p < 0.05, **p < 0.01, ***p < 0.001.

(B) UMAP embedding of single-cell RNA sequencing profile from Lin^{POS} and alveolar organoids colored by cell types/states, showing heterogeneous nature of the organoids. Inbox describes the origin of the cell sources in the UMAP.

(C) Dot plot describing differential marker gene expression level and cell proportion within a cluster, by cell types/states.

(D) UMAP plots showing transcript expression of AT2 lineage markers, *SFTPC*, *HOPX*, *SLC34A2*, and tip progenitor marker, *SOX9*, in the organoids.

(E) Immunofluorescent analysis of Lin^{POS} and alveolar organoids. Asterisk (*) indicates a lumen. DAPI, nuclei. Scale bars, 50 μm.

(F) qRT-PCR of *NKX2.1* and *SFTPC* in 7–9 pcw tip and Lin^{POS} organoids cultured in the SN medium or in DAPT/DCI/CHIR plus SB. Data were normalized to EPCAM$^+$ cells freshly isolated from 20 pcw tip tissues; mean ± SD of three biological replicates. Significance was evaluated by one-way ANOVA with Tukey multiple comparison post-test; ****p < 0.0001.

(G) Electron microscopy images of the alveolar organoids (left) and the Lin^{POS} organoids (right). LBs, lamellar bodies; PC, projection core; MV, microvilli; GC, glycogen; and PLM, primitive lipid membrane within a pool of monoparticulate glycogen at an early stage in the formation of LBs. Scale bars, 3 μm.

(H) Numbers of LBs per cells in the Lin^{POS} organoids (red) and alveolar organoids (blue). Immature and mature LBs were measured in total 40 cells from two biological samples for each condition. Significance evaluated by unpaired Student's t test; *p < 0.05, **p < 0.01, ***p < 0.001, ****p < 0.0001.

(I) Western blot showing NKX2.1 level and SFTPB/SFTPC processing in the 7–9 pcw, Lin^{POS}, and alveolar organoids. GAPDH (glyceraldehyde-3-phosphate dehydrogenase) was used for a loading control.

See also Figure S3.

CellPress
OPEN ACCESS

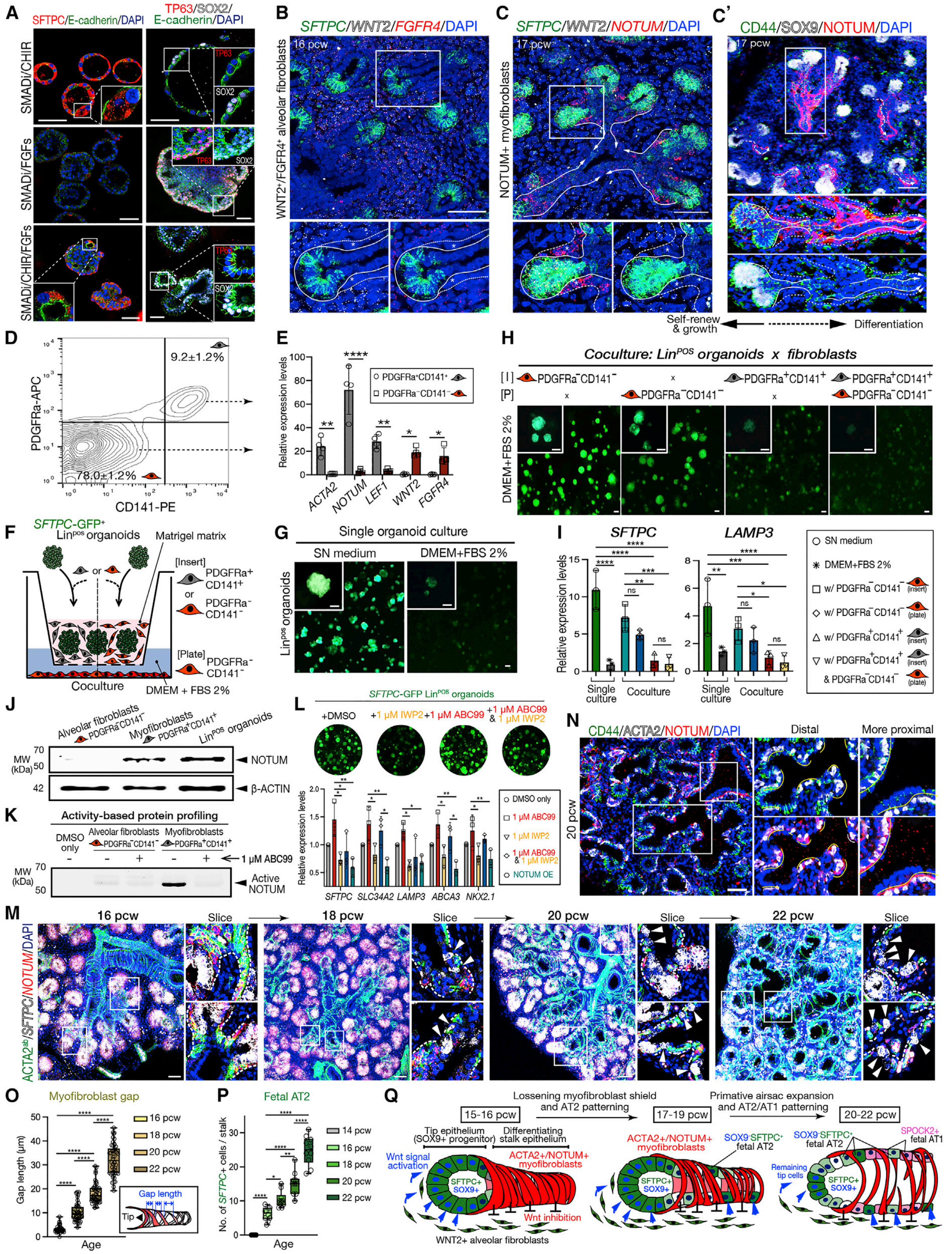

(legend on next page)

preventing the stalk epithelium from experiencing a high level of Wnt activity, allowing cells to turn off *SFTPC* and exit the tip fate. We identified surface antigens for the isolation of WNT2$^+$ fibroblasts or NOTUM$^+$ myofibroblasts (Figures 4E and S5F–S5H). Isolated PDGFRA$^+$CD141$^+$ myofibroblasts express high levels of *ACTA2*, *NOTUM*, and *LEF1*. Whereas PDGFRA$^-$CD141$^-$ fibroblasts express high levels of *WNT2* and *FGFR4* (Figures 4D and 4E). This gene expression is maintained if the cell types are cultured individually for 14 days. When freshly isolated fibroblasts and myofibroblasts were co-cultured, the levels of myofibroblast *LEF1* and *NOTUM* increased, suggesting that they are indeed responding to WNT2 from the fibroblasts (Figure S5I).

We asked whether co-culture with the PDGFRA$^-$CD141$^-$ fibroblasts, or PDGFRA$^+$CD141$^+$ myofibroblasts, could affect *SFTPC* expression in the Lin^{POS} organoids (Figure 4F). Lin^{POS} organoids robustly express *SFTPC*-GFP when cultured in SN medium, but not in 2% FBS (Figure 4G). However, co-culture with PDGFRA$^-$CD141$^-$ fibroblasts can substitute for SN medium and maintain *SFTPC*-GFP and endogenous *SFTPC* and *LAMP3* (Figures 4H and 4I). By contrast, co-culture of the Lin^{POS} organoids with PDGFRA$^+$CD141$^+$ myofibroblasts, or both PDGFRA$^+$CD141$^+$ myofibroblasts and PDGFRA$^-$CD141$^-$ fibroblasts, did not support AT2 gene expression. We propose that *in vivo* WNT2-expressing alveolar fibroblasts promote *SFTPC* expression in the distal tip. Moreover, that differentiating stalk cells are protected from the Wnt signal by the NOTUM-secreting myofibroblasts allowing them to turn off *SFTPC* and enter a differentiation program (Figure S5M).

To test the specific role of NOTUM-mediated inhibition of Wnt, we performed loss- and gain-of-function studies by treating with a NOTUM inhibitor ABC99, by overexpressing NOTUM (NOTUM-OE), or using conditioned medium (NOTUM-CM) (Figures 4J, 4K, and S5J–S5L).[16,17] In the co-culture, treatment with 1-μM ABC99 efficiently inhibited mesenchymal and epithelial NOTUM activity, resulting in prolonged expression of *SFTPC*-GFP reporter even in the absence of exogenous Wnt agonists (Figure S5J). By contrast, the Lin^{POS} organoids co-cultured with the NOTUM-OE fibroblasts, or treated with the NOTUM-CM, lose *SFTPC*-GFP reporter expression, which is restored by ABC99 treatment (Figures S5K and S5L). This shows that Wnt activity in the Lin^{POS} organoids relies on both paracrine signaling from the fibroblasts and partly on autocrine Wnt. Indeed, treatment of 1-μM IWP2, an inhibitor of Porcupine that is essential for Wnt secretion and activation, resulted in reduction of *SFTPC*-GFP and AT2 gene transcription in the Lin^{POS} organoids, which could be restored by ABC99 treatment (Figure 4L). These data demonstrate that NOTUM activity in the distal tip epithelium and the myofibroblasts function as a highly efficient Wnt regulatory machinery that controls epithelial tip cell fate in the developing lung.

Figure 4. Spatial patterning of the differentiating alveolar epithelium by modulating Wnt signaling activity

(A) Immunofluorescence analysis of the tip organoids at passage zero cultured in the different culture conditions. Antibodies against E-cadherin, SFTPC, TP63, and SOX2 were used. Scale bars, 20 μm.

(B and C) Frozen sections of 15–17 pcw human fetal lung stained by *in situ* HCR and/or antibodies. (B) 16 pcw, *SFTPC*, *WNT2*, and *FGFR4* probes. (C) 17 pcw, *SFTPC*, *WNT2*, and *NOTUM* probes. (C′) 17 pcw, CD44, SOX9, and NOTUM antibodies. Asterisks (B and C) represent *NOTUM*$^+$ myofibroblasts in the tissues. Lines and dashed lines indicate the boundaries of epithelial cells and myofibroblasts, respectively. Scale bars, 50 μm.

(D) Isolation of PDGFRA$^+$CD141$^+$ myofibroblasts and PDGFRA$^-$CD141$^-$ alveolar fibroblasts from human fetal lung tissues at 17–21 pcw using a combination of PDGFRA-APC and CD141-PE antibodies.

(E) qRT-PCR of PDGFRa$^+$CD141$^+$ myofibroblasts and PDGFRa$^-$CD141$^-$ alveolar fibroblasts freshly isolated from 17 to 21 pcw human lung tissues. Data were normalized to the total isolated fibroblast population; mean ± SD of biological 4 replicates. Significance was evaluated by unpaired Student's t test; *p < 0.05, **p < 0.01, ***p < 0.001.

(F) Diagram illustrating *in vitro* co-culture of the isolated PDGFRa$^+$CD141$^+$ myofibroblasts and PDGFRa$^-$CD141$^-$ alveolar fibroblast with Lin^{POS} tip organoids expressing *SFTPC*-GFP.

(G and H) *SFTPC*-GFP signal of Lin^{POS} tip organoids.

(G) Cultured alone in self-renewing (SN) or DMEM + 2% FBS medium.

(H) Co-cultured with freshly isolated fibroblast subpopulations. I, insert; P, plate. Scale bars, 100 μm.

(I) qRT-PCR for *SFTPC* and *LAMP3*, 2 weeks after *in vitro* culture. Mean ± SD of 3 biological replicates. Significance was evaluated by one-way ANOVA; ns: not significant, *p < 0.05, **p < 0.01, ***p < 0.001.

(J) Western blot assay of NOTUM expression in PDGFRA$^-$CD141$^-$ alveolar fibroblasts, PDGFRA$^+$CD141$^+$ myofibroblasts, and Lin^{POS} organoids. β-ACTIN was used for a loading control. 52 kDa, NOTUM.

(K) Activity-based protein profiling assay of the alveolar fibroblasts and the myofibroblasts for detecting an enzymatically active form of NOTUM.

(L) An inhibitor of Porcupine (1-μM IWP2) or NOTUM (1-μM ABC99) was added to the Lin^{POS} organoids in the SN medium for 1 week and analyzed by qRT-PCR. The medium was replaced every 2 days. DMSO only, a positive control. NOTUM overexpression (OE), a negative control. Data were normalized to the Lin^{POS} organoids treated with DMSO only; mean ± SD of biological 3 replicates. Significance was evaluated by one-way ANOVA; *p < 0.05, **p < 0.01.

(M) Time course analysis of spatial configuration of ACTA2$^+$*NOTUM*$^+$ myofibroblasts and *SFTPC*$^+$ tip epithelium in the human fetal lung tissues at 16, 18, 20, and 22 pcw, by *in situ* HCR followed by antibody immunostaining. Red, *NOTUM*; white, *SFTPC*; green, ACTA2. Arrowheads and dashed lines in "slice" images indicate fetal AT2 cells and myofibroblasts at the stalk regions, respectively. The *SFTPC*$^+$ fetal AT2 cells are located at the gap between discontinued lines of the myofibroblasts. Thickness, 50 μm.

(N) Immunofluorescence analysis of 20 pcw human fetal lung using antibodies against CD44, ACTA2, and NOTUM. Yellow lines indicate ACTA2$^+$NOTUM$^+$ myofibroblasts.

(O) Measurement of gap length between the myofibroblasts surrounding the stalk epithelial tubes. 50 measurements from 2 biological replicates at each age. Significance was evaluated by one-way ANOVA; *p < 0.05, **p < 0.01, ***p < 0.001, ****p < 0.0001.

(P) Number of *SFTPC*$^+$ fetal AT2 cells was counted in the developing fetal lungs at 14, 16, 18, 20, and 22 pcw. Significance was evaluated by one-way ANOVA; *p < 0.05, **p < 0.01, ***p < 0.001, ****p < 0.0001.

(Q) Diagram describes the emergence of alveolar-type cells along with conformational changes of myofibroblasts.

DAPI, nuclei.

See also Figures S4 and S5.

CellPress
OPEN ACCESS

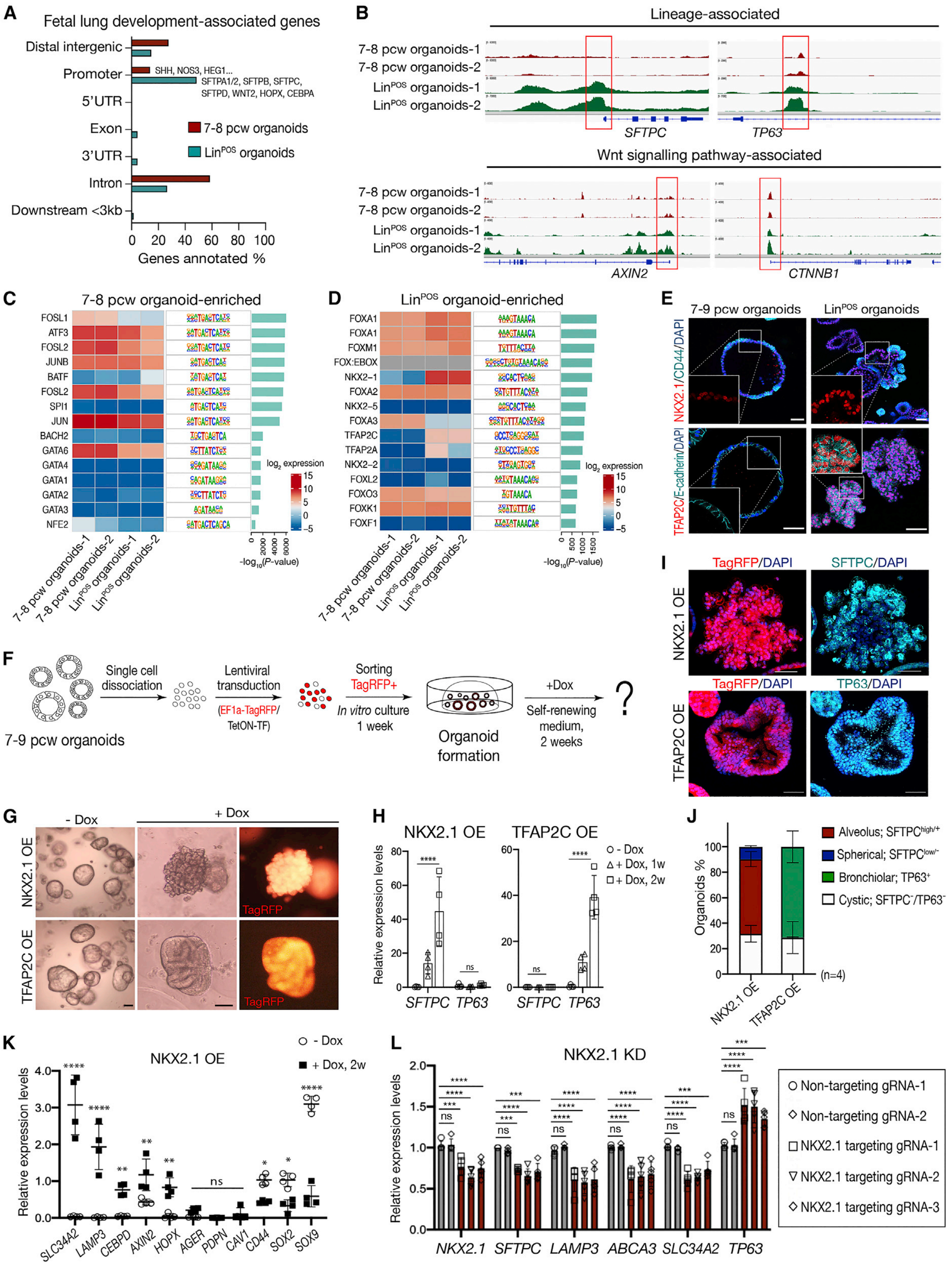

(legend on next page)

The distribution of Wnt and NOTUM-expressing cells is spatiotemporally regulated *in vivo*, consistent with an additional role in AT2 patterning. At 16 pcw the ACTA2$^+$*NOTUM*$^+$ myofibroblasts tightly shield the adjacent lower tip and the stalk regions, likely blocking Wnt signaling and allowing the tip cells to turn off *SFTPC* (and *SOX9*) and enter the stalk state. At 18–22 pcw, the myofibroblasts became loosely coiled and the stalk epithelium between them is now exposed to the *WNT2*$^+$ alveolar fibroblasts (Figures 4M–4O and 4Q). Simultaneously, *SFTPC*$^+$ differentiating AT2 cells were observed in the stalk and increased in number over time, filling the gaps between the myofibroblasts (Figures 4M, 4P, 4Q, S5N, and S5O; Videos S1, S2, and S3). At 20 pcw onward, *NKX2.1*$^+$, *SPOCK2*$^+$ differentiating AT1 cells emerged at the *SFTPC*$^-$ stalk regions and were aligned with the myofibroblasts (Figures 4Q, S5O, and S5P). Overall, these data show dynamic, spatiotemporal changes of the myofibroblasts in the alveolar niche of the late-stage lungs and support the concept that the differentiating alveolar epithelium is patterned by the myofibroblasts (Figures 4Q and S5M).

NKX2.1 is a major driving force for alveolar differentiation in the tip epithelial organoids

To identify putative TFs for cell differentiation, we analyzed chromatin accessibility of 7–8 pcw and Lin^{POS} organoids by bulk ATAC-seq (assay for transposase-accessible chromatin using sequencing). There were ~2-fold more differentially open chromatin regions in the Lin^{POS} than 7–8 pcw organoids, consistent with increased cell-type complexity of Lin^{POS} organoids (Figure S6A; Table S2). The gene feature distribution of the differentially opened chromatin was similar in both organoid types (Figure S6B). GO analysis of the genes nearest to differentially open chromatin was consistent with the RNA-seq data (Figures 2 and S6C–S6E). However, a much higher proportion of lung-development-associated genes had open chromatin at the promoter regions in the Lin^{POS} organoids (Figure 5A). For example, the promoter regions of lung differentiation genes *SFTPC*, *TP63*, and *CD36* and Wnt signaling genes, *AXIN2*, *CTNNB1*, *DVL3*, *LRRK2*, and *TCF7L1*, were more accessible in Lin^{POS} than 7–8 pcw organoids (Figures 5B, S6D, and S6E). These data strongly suggest that the chromatin accessibility of the Lin^{POS} organoids is intrinsically more favorable for lineage differentiation than the 7–8 pcw tip organoids, consistent with our functional assays.

To predict which TFs control cell fate, we performed TF motif analysis in the differential ATAC-seq peaks and compared this with our RNA-seq data to focus on TFs whose binding sites and RNA levels changed concordantly. In the 7–8 pcw organoids, the motifs for FOSL1 and GATA6 binding were predictive of function (Figures 5C and S6F). Moreover, GATA6 OE in the Lin^{POS} organoids resulted in a fate conversion of 10%–15% of the Lin^{POS} organoids to cystic Lin^{NEG} organoids (Figures S6G–S6I). In the Lin^{POS} organoids, NKX2.1 and TFAP2C motifs were accessible, and these TFs were highly transcribed (Figure 5D). Immunostaining confirmed that NKX2.1 is more strongly expressed in the Lin^{POS} than 7–8 pcw organoids. Moreover, TFAP2C was absent in 7–8 pcw organoids, but ubiquitous in the Lin^{POS} organoids (Figure 5E). *In vivo, NKX2-1* transcripts were most highly expressed in the tip and reduced in the stalk and *SCGB3A2*$^+$ distal airway regions, whereas *TFAP2C* was expressed in the airway epithelium (Figure S6J–S6L).

OE of NKX2.1 and TFAP2C in undifferentiated 7–9 pcw organoids tested whether either factor was sufficient to induce differentiation to the alveolar or airway lineages (Figure 5F). NKX2.1 OE resulted in ~60% of the 7–9 pcw organoids acquiring alveolar-like structure with high levels of *SFTPC* (Figures 5G–5J and S6M). NKX2.1 also upregulated other AT2 markers including *SCL34A2*, *LAMP3*, *CEBPD*, *HOPX*, and *AXIN2* but downregulated tip markers *SOX9*, *SOX2*, and *CD44* (Figure 5K). In contrast, TFAP2C OE caused ~70% of the organoids to form bronchiolar-like structures and significantly increased basal cell markers TP63/*P63*, *KRT5*, and *NGFR*, (Figures 5G–5J, S6M, and S6N). NKX2.1 and TFAP2C function as key regulators of differentiation toward AT2 and basal cell lineages respectively.

The Lin^{POS} organoids co-express high levels of NKX2-1 and TFAP2C (Figure 5E) yet are comprised of distinct SOX9/SFTPC$^+$ tip and TP63^{Lo} central regions (Figure 2). We analyzed the relationship between NKX2.1 and TFAP2C by simultaneous

Figure 5. Identification of key transcription factors controlling airway and alveolar lineage differentiation using the organoid system

(A) Genomic distribution of differentially accessible chromatin regions associated with human fetal lung development between 7 and 8 pcw and Lin^{POS} organoids.

(B) Representative ATAC-seq tracks visualized in Integrative Genomics Viewer (IGV) at *SFTPC*, *TP63*, *AXIN2*, and *CTNNB1*. Red box indicates the promoter.

(C and D) HOMER motif analysis coupled with RNA-seq data. The top 15 most highly enriched motifs and TF gene expression level (heatmap) are shown for 7–8 pcw (C) and Lin^{POS} organoids (D).

(E) 7–8 pcw and Lin^{POS} organoids stained with antibodies against NKX2.1, TFAP2C, CD44, and E-cadherin.

(F) Diagram showing doxycycline-inducible overexpression of NKX2.1 and/or TFAP2C in 7–9 pcw organoids. Constitutively expressed TagRFP was used for sorting transduced cells.

(G) Morphology of the 7–9 pcw organoids overexpressing NKX2.1 or TFAP2C for 2 weeks. Scale bars, 100 μm.

(H) Relative mRNA levels of *SFTPC* and *TP63* were measured by qRT-PCR in NKX2.1- or TFAP2C-OE 7–9 pcw organoids. Data were normalized to EPCAM$^+$ cells freshly isolated from 20 pcw tip tissues; mean ± SD of four biological replicates. Significance was evaluated by one-way ANOVA with Tukey multiple comparison post-test; ns: not significant, ****$p < 0001$.

(I and J) SFTPC and TP63 antibody staining of 7–9 pcw organoids overexpressing NKX2.1 or TFAP2C for 2 weeks (I). The proportion of the organoids positively stained in (J) was measured based on morphology and signal intensity. 4 biological replicates.

(K) qRT-PCR of 7–9 pcw organoids overexpressing NKX2.1 for 2 weeks. Data were normalized to EPCAM$^+$ cells freshly isolated from 20 pcw tip tissues; mean ± SD of four biological replicates. Significance was evaluated by one-way ANOVA with Tukey multiple comparison post-test; ns: not significant, *$p < 0.05$, **$p < 0.01$, ***$p < 0.001$, and ****$p < 0.0001$.

(L) Knockdown (KD) of endogenous *NKX2.1* in the Lin^{POS} organoids by CRISPR-dCas9-KRAB system. Data were normalized to non-targeting gRNAs; mean ± SD of 5 biological replicates. Significance was evaluated by one-way ANOVA with Tukey multiple comparison post-test; ns: not significant, *$p < 0.05$, **$p < 0.01$, ***$p < 0.001$, ****$p < 0.0001$.

DAPI indicates nuclei. Scale bars, 50 μm.

See also Figure S6 and Table S2.

CellPress
OPEN ACCESS

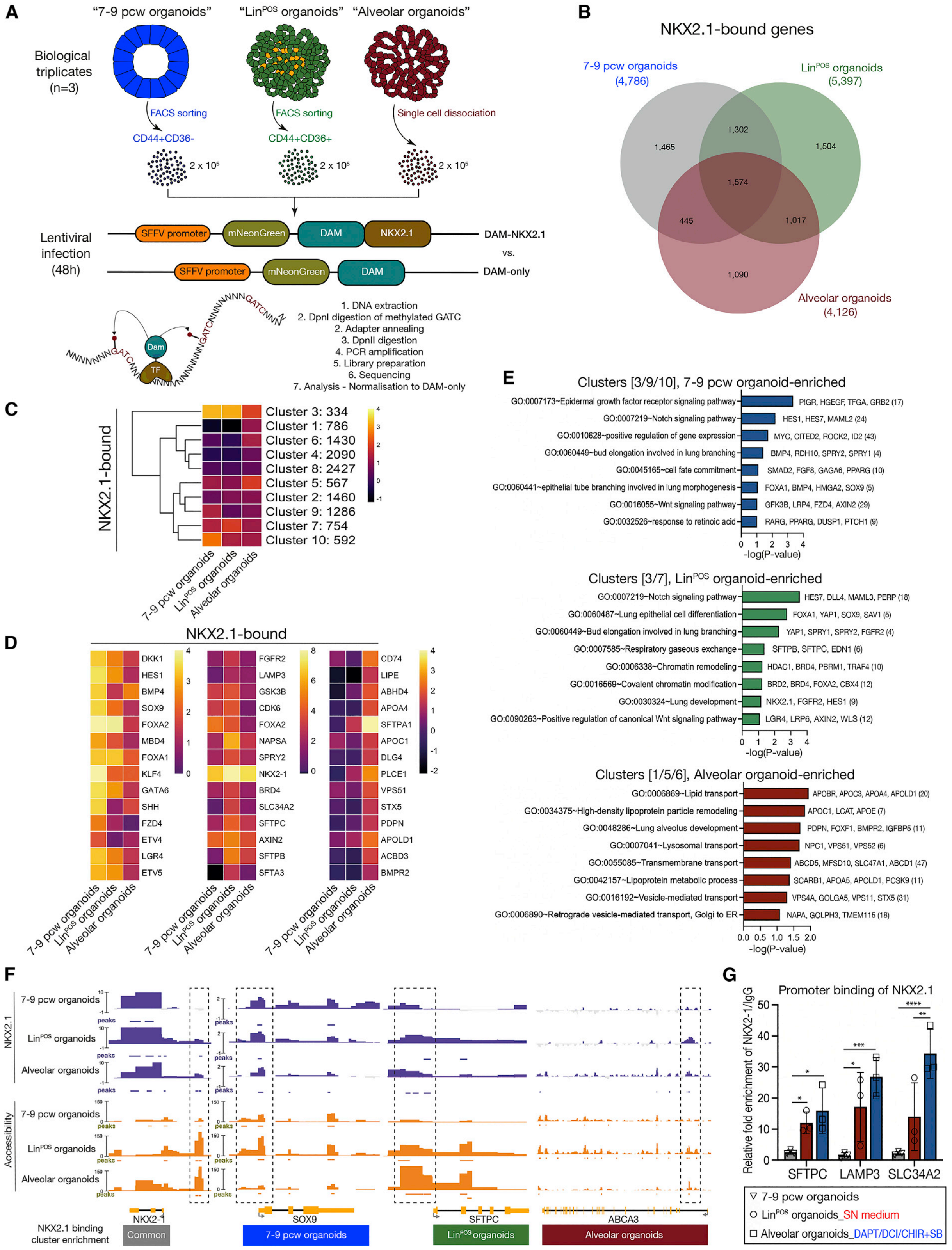

(legend on next page)

OE in the 7–9 pcw organoids. NKX2.1/TFAP2C OE organoids were highly folded, similar to NKX2-1 OE. Furthermore, TP63 was barely detectable, but SFTPC was markedly induced (Figure S6O). When Lin^{POS} organoids were cultured in SMADi/CHIR/FGF7, *NKX2.1*, and *SFTPC* were high, but *TP63* and *TFAP2C* were low. Whereas in SMADi/FGF7 (without the Wnt agonist), NKX2.1 deceased, *SFTPC* turned off and *TP63* and *TFAP2C* were robustly expressed (Figure S6P). These data demonstrate that high NKX2.1 expression, in combination with Wnt signaling, suppresses the airway lineages, explaining why TP63 expression is low in the Lin^{POS} organoids although TFAP2C is expressed (Figure S6P). Further support for the importance of NKX2.1 in promoting alveolar and inhibiting airway differentiation came from *NKX2.1* and *TFAP2C* knockdown experiments in the Lin^{POS} organoids. A small decrease in *NKX2.1* expression was sufficient to decrease AT2-specific gene expression and increase *TP63*, while *TFAP2C* KD did not affect AT2 gene transcription (Figures 5L and S6Q). These data suggest that Wnt signaling is a critical upstream regulator of *NKX2.1* levels and alveolar gene expression via a Wnt-NKX2.1-alveolar lineage transcriptional program.

Differential binding of NKX2.1 orchestrates alveolar differentiation and functional maturation

To investigate how NKX2.1 regulates alveolar lineage differentiation, we analyzed NKX2.1 genomic occupancy by performing targeted DamID (DNA adenine methyltransferase identification).[18–20] We compared NKX2.1 binding in FACs isolated tip cells from 7 to 9 pcw organoids (early/mid stage tips), Lin^{POS} organoids (late-stage tips), and Lin^{POS} organoids differentiated to AT2 fate (AT2-like) (Figure 6A). Overall chromatin accessibility (assessed from background methylation in Dam-only controls) was similar in all cell types (clusters 2–4; Figure S7A). However, we noticed two clusters of genes (clusters 1/5; Figure S7A) with enrichment for GO terms related to LB and Wnt signaling (Figures S7B and S7C), which were more open in the Lin^{POS} organoids and AT2-like cells. This indicates that open chromatin for regions associated with alveolar differentiation and Wnt signaling is a feature of alveolar-fated cells, consistent with the ATAC-seq data (Figure 5).

The gene feature distribution and the numbers of NKX2.1-bound peaks were similar in all organoid types (Figures 6B and S7D; Table S4). However, *k*-means clustering revealed a dynamic distribution of NKX2.1 binding across samples (Figures 6C and 6D). All cell types shared clusters 2, 4, and 8, related to Wnt signaling, cell-cycle, and cell-cell interactions (Figure S7E). We also annotated cell-type-enriched clusters based on NKX2.1 binding intensity (Figures 6C and 6D). The 7–9 pcw and Lin^{POS} tip NKX2-1 binding clusters were enriched for genes with GO terms related to the Notch/Wnt signaling pathways and lung bud elongation (Figures 6D–6F). However, the Lin^{POS} tips also had differential enrichment for GO terms for respiratory gaseous exchange, lung development, and chromatin remodeling, suggesting that the binding preferences of NKX2.1 shifted to alveolar lineage-associated genes to initiate differentiation (Figures 6D–6F). Strikingly, bound genes in the AT2-like cells were enriched with GO terms associated with lipid transport, lung alveolus development, lysosomal/transmembrane transport, and vesicle-mediated transport (Figures 6D–6F), indicating that NKX2.1 directly promotes surfactant protein synthesis, trafficking, and secretion. These data are consistent with the observation that the differentiated AT2-like cells showed a higher production of mature SFTPC/SFTPB and transport of mature LBs (Figure 3). NKX2.1 therefore directly activates genes related to surfactant production and secretion in alveolar-fated cells. We confirmed this for *SFTPC*, *LAMP3*, and *SLC34A2* by chromatin immunoprecipitation (ChIP)-qPCR (Figure 6G).

Motif enrichment analysis showed that NKX2.1-binding sites commonly shared motifs for NKX2.1 and FOXP1, regulators of lung endoderm development[21] (Figure S7F; Table S4), and a zinc-finger protein, ZNF770. We also found putative co-TFs differentially enriched in each organoid type (Figure S7G; Table S4), suggesting additional TFs work with NKX2.1 to coordinate alveolar differentiation and maturation. The combination of chromatin accessibility changes, differential binding, and cooperation with other factors provide a framework for the dynamic activity of NKX2.1 over the course of alveolar-fate acquisition.

Organoid assays can be used to predict the effects of human genetic variation

OE of NKX2.1 lacking the homeodomain showed that DNA binding is essential for AT2 differentiation (Figures 7A and 7B). Naturally occurring human variants in the NKX2.1 homeodomain have been described.[22–24] Many are associated with acute

Figure 6. Differential binding of NKX2.1 orchestrates alveolar differentiation and functional maturation

(A) Diagram describing the experimental scheme from targeted DamID. 2 × 10^5 cells from three types of organoids transduced with a lentiviral vector harboring either DAM only or DAM-NKX2.1 were used for targeted DamID to analyze NKX2.1-binding pattens.

(B) Venn diagram showing the number of NKX2.1-bound genes for each organoid sample.

(C and D) Heatmaps illustrating k-means clustering (C) of NKX2.1-bound genes across the organoid samples and the representative genes (D) that are relatively highly enriched in each sample. Colors represent binding intensities from genes associated with peaks, which was averaged across gene bodies, including −1 kb from the TSS (transcription start site).

(E) GO enrichment analysis of biological process-associated GO terms on the clusters highly enriched in the 7–9 pcw tip organoids (clusters 3/9/10), Lin^{POS} orgnaoids (clusters 3/7), and alveolar organoids (clusters 1/5/6). Representative genes associated with each GO term with the total number of genes in brackets were shown next to each graph bar.

(F) Representative targeted DamID tracks of Dam-NKX2.1 (NKX2.1-bound, purple) and Dam only (chromatin accessibility, orange) at the genes commonly shared (NKX2.1) differentially enriched (SOX9, SFTPC, and ABCA3) in the organoid samples. Peaks are described below the tracks. Black dashed boxes indicate the location of promoter regions.

(G) Chromatin immunoprecipitation (ChIP)-qPCR analysis for quantifying relative enrichment of NKX2.1 binding on the promoter regions of type 2 alveolar lineage markers, *SFTPC*, *LAMP3*, and *SLC34A2* in 7–9 pcw, Lin^{POS}, and alveolar organoids. Data were normalized to the IgG control; mean ± SD of three biological replicates.

See also Figure S7 and Table S4.

CellPress
OPEN ACCESS

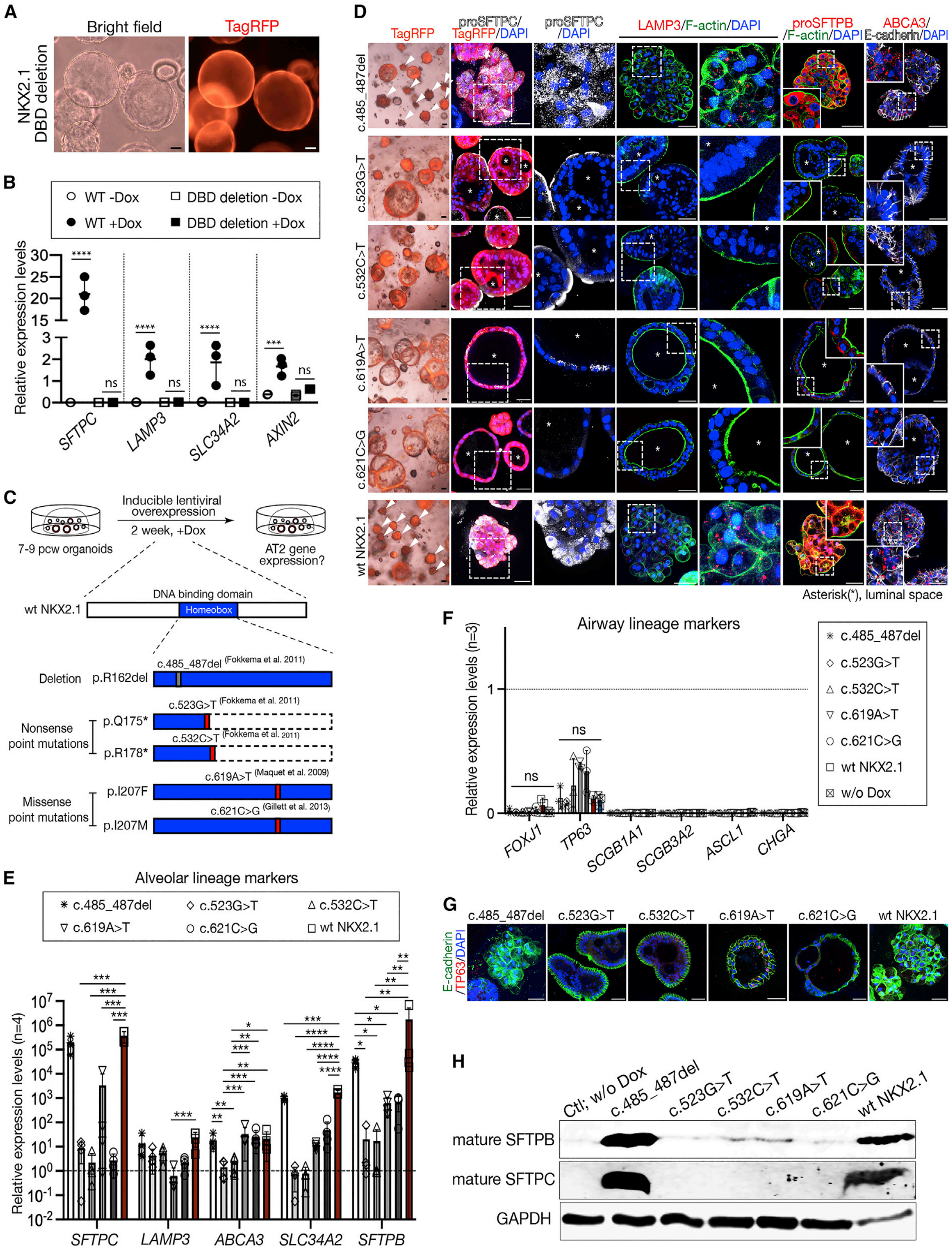

(legend on next page)

respiratory failure; others are predicted to be pathogenic. We hypothesized that the NKX2.1 OE assay would be a simple method to determine the effects of these variants on AT2-specific gene expression (Figure 7C). The variants differentially affected organoid morphology, cell fate specification, and surfactant protein production (Figures 7D–7G) with c.621C>G abrogating AT2 differentiation, c.619A>T causing partial AT2 differentiation, but the predicted pathogenic c.485_487 deletion behaving indistinguishably to the wild type. Expression of the NKX2.1 variants c.523G>T and c.532C>T resulted in the production of mis-localized pro-SFTPC, which was not processed to the mature form (Figures 7D and 7H), consistent with a defect in the surfactant processing and trafficking pathway.[25] NKX2.1 therefore promotes multiple aspects of AT2 differentiation, not simply *SFTPC* transcription and, that the underlying pathology of the *NKX2.1* variants is due to inadequate surfactant production.

DISCUSSION

We show that the distal tip cells at 17–22 pcw of human lung development are intrinsically different to the earlier stages; retaining progenitor status yet exhibiting aspects of AT2 gene expression. Late-tip cell (Lin^{POS}) organoids self-renew, capture features of late-stage human lung development, have extensive open chromatin, and can be readily differentiated to AT2-like cells. We have used this organoid system to demonstrate that Wnt signaling triggers NKX2.1-dependent human AT2 cell differentiation. Additionally, we show that antagonistic signaling interactions between differentiating fibroblasts and myofibroblasts in the developing alveolar niche provide a spatial constraint to Wnt activity, patterning the epithelium into specific lineages. Our organoid systems are readily genetically manipulated and can be used to study human genetic variation.

We demonstrate that the molecular acquisition of alveolar features precedes morphological changes occurring in the tip epithelial progenitors. Our Lin^{POS} organoids are derived from the CD44$^+$,CD36$^+$ 17–21 pcw tips. The CD44$^+$,CD36$^+$ cells are located at the tips of the organoids, maintain expression of late-tip markers, SN, and give rise to airway-fated cells in the organoid center (Figure 2). When provided with appropriate cues they differentiate to an AT2-like cell (Figure 3). *In vivo*, tip cells acquire AT2 markers gradually between 13 and 15 pcw (Figure 1). We hypothesize that during this transition period (~13–15 pcw) the tips are generating the final branch of the airway epithelium and at ~15 pcw switch their competence and generate alveolar-fated daughter cells. However, due to well-documented tip progenitor plasticity in transplantation assays,[26,27] it is not currently possible to test this definitively.

We identify a Wnt-NKX2.1 axis as a key driver of human AT2 fate and patterning. Wnt signaling promotes AT2 differentiation of the tip epithelium *in vitro* and *in vivo* (Figures 3 and 4) while suppressing airway fate (Figure S6). This is consistent with previous reports in mouse.[28] Similarly, human NKX2.1$^+$ lung progenitors derived from PSCs expressed alveolar epithelial markers in response to Wnt.[6] Our experiments with primary tissue provide a molecular and cellular context to the Wnt-induced alveolar patterning. Our data support a model in which opposing signals from differentiating alveolar fibroblasts (WNT2) and myofibroblasts (NOTUM) spatially restrict late-tip and AT2 identity in the late-stage human fetal lung (Figure 4). This is analogous to a recent mouse report where developing AT1 cells are aligned with, and signal to, differentiating myofibroblasts.[29] It will be interesting to test in the future whether myofibroblast inhibition of AT2 cell fate also occurs in pulmonary fibrosis where myofibroblasts are expanded and AT2 cells lost.

We demonstrate that NKX2.1 is a key upstream TF driving the alveolar program while suppressing the airway program (Figure 5), consistent with reported roles in lung cancers.[30–32] Our independent single-cell ATAC-seq data of the developing human lungs from 5 to 22 pcw showed that *in vivo* alveolar-fated cells, including AT1, AT2, and late-stage tip cells are highly enriched with NKX2.1 motifs, while the basal cells have TP63 motifs.[7] The current experimental data show that differential binding of NKX2.1 facilitates alveolar differentiation and functional maturation (Figures 5 and 6), also explaining why pathogenic NKX2.1 mutations are incompatible with neonatal life (Figure 7). Some of the patient NKX2.1 mutations tested caused a partial/stalled AT2 differentiation phenotype, which is potentially rescuable.

By contrast, ectopic expression of TFAP2C promoted basal cell markers, but not other airway lineages (Figure 5). This could mean the culture conditions are permissive only for basal cell differentiation. Alternatively, TFAP2C may be specific for basal cell specification. The latter interpretation would be consistent with a report that TFAP2C acts as an upstream TF to TP63 during epidermal maturation.[33] FGF signaling also promotes airway fate in the absence of Wnt signaling (Figure 4); consistent with our recent *in vivo* finding that airway fibroblasts form a proximal airway niche by secreting FGF7 and providing a physical barrier to block canonical Wnt ligands from reaching the airway epithelial layer.[7] However, hPSC can be differentiated to AT2 cells in the presence of FGF7[14] and we cannot exclude a role for FGF signaling in alveolar epithelial differentiation/proliferation.

We have identified a distinct late-tip progenitor cell in the developing human lung. Culture of these cells as organoids has allowed us to define the cellular and molecular roles of Wnt signaling and NKX2.1 in human alveolar cell development.

Figure 7. Analysis of naturally occurring human genetic variation using organoid assays

(A and B) Morphology (A) and gene expression profile (B) in 7–9 pcw tip organoids overexpressing wild-type NKX2.1, or a NKX2.1 lacking a DNA-binding domain (DBD deletion), cultured for 2 weeks in the presence, or absence, of doxycycline (±DOX).

(C) Diagram describing overexpression of wild-type and mutant forms of NKX2.1 in 7–9 pcw organoids using doxycycline-inducible lentiviral system. Individually, five different mutations were introduced into the DNA-binding homeobox domain; deletion of Arg[162] (R162del),[22] two nonsense point mutations (Q175*, R178*),[22] and two missense point mutations (I207F, I207M)[23,24] were tested.

(D–H) Morphology and immunostaining (D and G), qRT-PCR (E and F), and western blot (H) analysis of 7–9 pcw organoids following overexpression of wild-type or mutant human NKX2.1 for 2 weeks. Data were normalized to doxycycline-non-treated lines (E) or to fresh lung-tissue-derived airway epithelial cells (F); mean ± SD of 4 (E) or 3 (F) biological replicates, respectively. Significance was evaluated by one-way ANOVA with Tukey multiple comparison post-test; *$p < 0.05$, **$p < 0.01$, ***$p < 0.001$. Western blot showing mature SFTPB and SFTPC. GAPDH was used for a loading control for the western blot assay. DAPI indicates nuclei. Scale bars, 50 μm.

Moreover, this differentiating organoid system will be useful for understanding the next phases of alveolar maturation in the developing human lung.

Limitations of the study

We have developed organoid techniques for studying alveolar development in human fetal lungs. Isolation of specific fetal lung cells for organoid cultures can be performed using flow cytometry (Figures 2 and 4). Flow cytometry is inherently limited by differential epitope sensitivity to the enzymes used to generate single-cell suspensions. We controlled this using qRT-PCR to check the identity of our sorted cell populations, but we cannot exclude the possibility of some contamination from other cell lineages.

The organoid assay provided a convenient tool for testing predicted human pathogenic variants of *NKX2.1* (Figure 7). These naturally occurring mutations are observed as heterozygotes and predicted to function as dominant-negative, or neomorphic, proteins. Not all the predicted pathogenetic *NKX2.1* mutations resulted in phenotypes, and we are unable to distinguish whether these variants truly behave as wild type, or whether this is a limitation of the assay.

STAR★METHODS

Detailed methods are provided in the online version of this paper and include the following:

- KEY RESOURCES TABLE
- RESOURCE AVAILABILITY
  - Lead contact
  - Materials availability
  - Data and code availability
- EXPERIMENTAL MODEL AND SUBJECT DETAILS
  - Human embryonic and fetal lung tissue
- METHOD DETAILS
  - *In vitro* culture of human fetal lung organoids
  - Isolation of tip epithelial cells, myofibroblasts, and alveolar fibroblasts
  - Alveolar differentiation of Lin^{POS} organoids
  - Lentiviral transduction
  - Immunostaining of organoids and lung tissues
  - *In situ* hybridization chain reaction (*in situ* HCR)
  - RNA extraction, cDNA synthesis, qRT-PCR analysis, and bulk RNA-sequencing
  - Immunoblotting
  - Activity-based protein profiling on SDS-PAGE gel
  - Chromatin immunoprecipitation
  - Bulk ATAC-sequencing
  - Electron microscopy imaging
  - Organoid single-cell RNA sequencing
  - Targeted DamID sequencing
- QUANTIFICATION AND STATISTICAL ANALYSIS

SUPPLEMENTAL INFORMATION

Supplemental information can be found online at https://doi.org/10.1016/j.stem.2022.11.013.

ACKNOWLEDGMENTS

We acknowledge the Gurdon NGS core, Imaging Facility and Dr. Karin Mueller of Cambridge Advanced Imaging Centre. We thank Professor Azim Surani for epigenetics advice and Dr. Krzysztof Polanski for mapping organoid scRNA-seq data. K.L. is supported by Basic Science Research Program through the National Research Foundation of Korea (NRF) funded by the Ministry of Education (2018R1A6A3A03012122). D.S. is supported by the Wellcome Trust (109146/Z/15/Z) and the Department of Pathology, University of Cambridge. E.L.R. is supported by the Medical Research Council (MR/P009581/1; MR/S035907/1). K.B.M. and S.A.T. by the MRC (MR/S035907/1) and Wellcome (WT211276/Z/18/Z and Sanger core grant WT206194). A.H.B. lab was funded by Wellcome Trust Senior Investigator Award (103792) and the Royal Society Darwin Trust Research Professorship. Core funding to the Gurdon Institute from the Wellcome Trust (203144/Z/16/Z) and CRUK (C6946/A24843).

AUTHOR CONTRIBUTIONS

Conceptualization, K.L. and E.L.R.; methodology, investigation, and validation, K.L., D.S., and A.P.A.D.; software and formal analysis, K.L., W.T., P.H., and J.P.P.; writing – original draft, K.L.; writing – review & editing, E.L.R.; funding acquisition and supervision, E.L.R., S.A.T., J.C.M., K.B.M., and A.H.B.

DECLARATION OF INTERESTS

S.A.T. is a member of the scientific advisory board for the following companies: Biogen, Foresite Labs, GSK, Qiagen, CRG Barcelona, Jax Labs, SciLife Lab, and Allen Institute. She is a consultant for Genentech and Roche. She is co-founder of Transition Bio and a member of the Board.

Received: February 1, 2022
Revised: October 2, 2022
Accepted: November 16, 2022
Published: December 8, 2022

REFERENCES

1. Burri, P.H. (1984). Fetal and postnatal development of the lung. Annu. Rev. Physiol. *46*, 617–628. https://doi.org/10.1146/annurev.ph.46.030184.003153.
2. Nikolić, M.Z., Sun, D., and Rawlins, E.L. (2018). Human lung development: recent progress and new challenges. Development *145*, dev163485. https://doi.org/10.1242/dev.163485.
3. Nikolić, M.Z., Caritg, O., Jeng, Q., Johnson, J.-A., Sun, D., Howell, K.J., Brady, J.L., Laresgoiti, U., Allen, G., Butler, R., et al. (2017). Human embryonic lung epithelial tips are multipotent progenitors that can be expanded in vitro as long-term self-renewing organoids. eLife *6*, e26575. https://doi.org/10.7554/eLife.26575.
4. Miller, A.J., Hill, D.R., Nagy, M.S., Aoki, Y., Dye, B.R., Chin, A.M., Huang, S., Zhu, F., White, E.S., Lama, V., et al. (2018). In vitro induction and in vivo engraftment of lung bud tip progenitor cells derived from human pluripotent stem cells. Stem Cell Rep. *10*, 101–119. https://doi.org/10.1016/j.stemcr.2017.11.012.
5. Sun, D., Evans, L., Perrone, F., Sokleva, V., Lim, K., Rezakhani, S., Lutolf, M., Zilbauer, M., and Rawlins, E.L. (2021). A functional genetic toolbox for human tissue-derived organoids. eLife *10*, e67886. https://doi.org/10.7554/eLife.67886.
6. McCauley, K.B., Hawkins, F., Serra, M., Thomas, D.C., Jacob, A., and Kotton, D.N. (2017). Efficient derivation of functional human airway epithelium from pluripotent stem cells via temporal regulation of Wnt signaling. Cell Stem Cell *20*, 844–857.e6. https://doi.org/10.1016/j.stem.2017.03.001.
7. He, P., Lim, K., Sun, D., Pett, J.P., Jeng, Q., and Polanski, K. (2022). A human fetal lung cell atlas uncovers proximal-distal gradients of differentiation and key regulators of epithelial fates. Cell *185*, 24. https://doi.org/10.1016/j.cell.2022.11.005.

8. Little, D.R., Lynch, A.M., Yan, Y., Akiyama, H., Kimura, S., and Chen, J. (2021). Differential chromatin binding of the lung lineage transcription factor NKX2-1 resolves opposing murine alveolar cell fates in vivo. Nat. Commun. *12*, 2509. https://doi.org/10.1038/s41467-021-22817-6.

9. Guillot, L., Carré, A., Szinnai, G., Castanet, M., Tron, E., Jaubert, F., Broutin, I., Counil, F., Feldmann, D., Clement, A., et al. (2010). NKX2-1 mutations leading to surfactant protein promoter dysregulation cause interstitial lung disease in "Brain-Lung-Thyroid Syndrome". Hum. Mutat. *31*, E1146–E1162. https://doi.org/10.1002/humu.21183.

10. Attarian, S.J., Leibel, S.L., Yang, P., Alfano, D.N., Hackett, B.P., Cole, F.S., and Hamvas, A. (2018). Mutations in the thyroid transcription factor gene NKX2-1 result in decreased expression of SFTPB and SFTPC. Pediatr. Res. *84*, 419–425. https://doi.org/10.1038/pr.2018.30.

11. Travaglini, K.J., Nabhan, A.N., Penland, L., Sinha, R., Gillich, A., Sit, R.V., Chang, S., Conley, S.D., Mori, Y., Seita, J., et al. (2020). A molecular cell atlas of the human lung from single-cell RNA sequencing. Nature *587*, 619–625. https://doi.org/10.1038/s41586-020-2922-4.

12. Ochs, M. (2010). The Closer we Look the more we See? Quantitative microscopic analysis of the pulmonary surfactant system. The closer we look the more we see? Cell. Physiol. Biochem. *25*, 27–40. https://doi.org/10.1159/000272061.

13. Weaver, T.E., Na, Na, C.L., and Stahlman, M. (2002). Biogenesis of lamellar bodies, lysosome-related organelles involved in storage and secretion of pulmonary surfactant. Semin. Cell Dev. Biol. *13*, 263–270. https://doi.org/10.1016/s1084952102000551.

14. Jacob, A., Morley, M., Hawkins, F., McCauley, K.B., Jean, J.C., Heins, H., Na, C.L., Weaver, T.E., Vedaie, M., Hurley, K., et al. (2017). Differentiation of human pluripotent stem cells into functional lung alveolar epithelial cells. Cell Stem Cell *21*, 472–488.e10. https://doi.org/10.1016/j.stem.2017.08.014.

15. Kakugawa, S., Langton, P.F., Zebisch, M., Howell, S., Chang, T.-H., Liu, Y., Feizi, T., Bineva, G., O'Reilly, N., Snijders, A.P., et al. (2015). Notum deacylates Wnt proteins to suppress signalling activity. Nature *519*, 187–192. https://doi.org/10.1038/nature14259.

16. Suciu, R.M., Cognetta, A.B., 3rd, Potter, Z.E., and Cravatt, B.F. (2018). Selective irreversible inhibitors of the Wnt-deacylating enzyme NOTUM developed by activity-based protein profiling. ACS Med. Chem. Lett. *9*, 563–568. https://doi.org/10.1021/acsmedchemlett.8b00191.

17. Pentinmikko, N., Iqbal, S., Mana, M., Andersson, S., Cognetta, A.B., 3rd, Suciu, R.M., Roper, J., Luopajärvi, K., Markelin, E., Gopalakrishnan, S., et al. (2019). Notum produced by Paneth cells attenuates regeneration of aged intestinal epithelium. Nature *571*, 398–402. https://doi.org/10.1038/s41586-019-1383-0.

18. Southall, T.D., Gold, K.S., Egger, B., Davidson, C.M., Caygill, E.E., Marshall, O.J., and Brand, A.H. (2013). Cell-type-specific profiling of gene expression and chromatin binding without cell isolation: assaying RNA Pol II occupancy in neural stem cells. Dev. Cell *26*, 101–112. https://doi.org/10.1016/j.devcel.2013.05.020.

19. Marshall, O.J., and Brand, A.H. (2015). damidseq_pipeline: an automated pipeline for processing DamID sequencing datasets. Bioinformatics *31*, 3371–3373. https://doi.org/10.1093/bioinformatics/btv386.

20. Marshall, O.J., Southall, T.D., Cheetham, S.W., and Brand, A.H. (2016). Cell-type-specific profiling of protein–DNA interactions without cell isolation using targeted DamID with next-generation sequencing. Nat. Protoc. *11*, 1586–1598. https://doi.org/10.1038/nprot.2016.084.

21. Shu, W., Lu, M.M., Zhang, Y., Tucker, P.W., Zhou, D., and Morrisey, E.E. (2007). Foxp2 and Foxp1 cooperatively regulate lung and esophagus development. Development *134*, 1991–2000. https://doi.org/10.1242/dev.02846.

22. Fokkema, I.F.A.C., Taschner, P.E.M., Schaafsma, G.C.P., Celli, J., Laros, J.F.J., and den Dunnen, J.T. (2011). LOVD v.2.0: the next generation in gene variant databases. Hum. Mutat. *32*, 557–563. https://doi.org/10.1002/humu.21438.

23. Gillett, E.S., Deutsch, G.H., Bamshad, M.J., McAdams, R.M., and Mann, P.C. (2013). Novel NKX2.1 mutation associated with hypothyroidism and lethal respiratory failure in a full-term neonate. J. Perinatol. *33*, 157–160. https://doi.org/10.1038/jp.2012.50.

24. Maquet, E., Costagliola, S., Parma, J., Christophe-Hobertus, C., Oligny, L.L., Fournet, J.-C., Robitaille, Y., Vuissoz, J.-M., Payot, A., Laberge, S., et al. (2009). Lethal respiratory failure and mild primary hypothyroidism in a term girl with a de novo heterozygous mutation in the TITF1/NKX2.1 gene. J. Clin. Endocrinol. Metab. *94*, 197–203. https://doi.org/10.1210/jc.2008-1402.

25. Dickens, J.A., Rutherford, E.N., Abreu, S., Chambers, J.E., Ellis, M.O., van Schadewijk, A., Hiemstra, P.S., and Marciniak, S.J. (2022). Novel insights into surfactant protein C trafficking revealed through the study of a pathogenic mutant. Eur. Respir. J. *59*, 2100267. https://doi.org/10.1183/13993003.00267-2021.

26. Laresgoiti, U., Nikolić, M.Z., Rao, C., Brady, J.L., Richardson, R.V., Batchen, E.J., Chapman, K.E., and Rawlins, E.L. (2016). Lung epithelial tip progenitors integrate glucocorticoid- and STAT3-mediated signals to control progeny fate. Development *143*, 3686–3699. https://doi.org/10.1242/dev.134023.

27. Shannon, J.M., Nielsen, L.D., Gebb, S.A., and Randell, S.H. (1998). Mesenchyme specifies epithelial differentiation in reciprocal recombinants of embryonic lung and trachea. Dev. Dyn. *212*, 482–494. https://doi.org/10.1002/(SICI)1097-0177(199808)212:4<482::AID-AJA2>3.0.CO;2-D.

28. Frank, D.B., Peng, T., Zepp, J.A., Snitow, M., Vincent, T.L., Penkala, I.J., Cui, Z., Herriges, M.J., Morley, M.P., Zhou, S., et al. (2016). Emergence of a wave of Wnt signaling that regulates lung alveologenesis by controlling epithelial self-renewal and differentiation. Cell Rep. *17*, 2312–2325. https://doi.org/10.1016/j.celrep.2016.11.001.

29. Zepp, J.A., Morley, M.P., Loebel, C., Kremp, M.M., Chaudhry, F.N., Basil, M.C., Leach, J.P., Liberti, D.C., Niethamer, T.K., Ying, Y., et al. (2021). Genomic, epigenomic, and biophysical cues controlling the emergence of the lung alveolus. Science *371*, eabc3172. https://doi.org/10.1126/science.abc3172.

30. Snyder, E.L., Watanabe, H., Magendantz, M., Hoersch, S., Chen, T.A., Wang, D.G., Crowley, D., Whittaker, C.A., Meyerson, M., Kimura, S., et al. (2013). Nkx2-1 represses a latent gastric differentiation program in lung adenocarcinoma. Mol. Cell *50*, 185–199. https://doi.org/10.1016/j.molcel.2013.02.018.

31. Mollaoglu, G., Jones, A., Wait, S.J., Mukhopadhyay, A., Jeong, S., Arya, R., Camolotto, S.A., Mosbruger, T.L., Stubben, C.J., Conley, C.J., et al. (2018). The lineage-defining transcription factors SOX2 and NKX2-1 determine lung cancer cell fate and shape the tumor immune microenvironment. Immunity *49*, 764–779.e9. https://doi.org/10.1016/j.immuni.2018.09.020.

32. Tata, P.R., Chow, R.D., Saladi, S.V., Tata, A., Konkimalla, A., Bara, A., Montoro, D., Hariri, L.P., Shih, A.R., Mino-Kenudson, M., et al. (2018). Developmental history provides a roadmap for the emergence of tumor plasticity. Dev. Cell *44*, 679–693.e5. https://doi.org/10.1016/j.devcel.2018.02.024.

33. Li, L., Wang, Y., Torkelson, J.L., Shankar, G., Pattison, J.M., Zhen, H.H., Fang, F., Duren, Z., Xin, J., Gaddam, S., et al. (2019). TFAP2C- and p63-dependent networks sequentially rearrange chromatin landscapes to drive human epidermal lineage commitment. Cell Stem Cell *24*, 271–284.e8. https://doi.org/10.1016/j.stem.2018.12.012.

34. Horlbeck, M.A., Gilbert, L.A., Villalta, J.E., Adamson, B., Pak, R.A., Chen, Y., Fields, A.P., Park, C.Y., Corn, J.E., Kampmann, M., et al. (2016). Compact and highly active next-generation libraries for CRISPR-mediated gene repression and activation. eLife *5*, e19760. https://doi.org/10.7554/eLife.19760.

35. Schneider, C.A., Rasband, W.S., and Eliceiri, K.W. (2012). NIH Image to ImageJ: 25 years of image analysis. Nat. Methods *9*, 671–675. https://doi.org/10.1038/nmeth.2089.

36. Choi, H.M.T., Schwarzkopf, M., Fornace, M.E., Acharya, A., Artavanis, G., Stegmaier, J., Cunha, A., and Pierce, N.A. (2018). Third-generation in situ hybridization chain reaction: multiplexed, quantitative, sensitive, versatile,

robust. Development *145*, dev165753. https://doi.org/10.1242/dev.165753.

37. da Huang, W., Sherman, B.T., and Lempicki, R.A. (2009). Systematic and integrative analysis of large gene lists using DAVID bioinformatics resources. Nat. Protoc. *4*, 44–57. https://doi.org/10.1038/nprot.2008.211.

38. Kuleshov, M.V., Jones, M.R., Rouillard, A.D., Fernandez, N.F., Duan, Q., Wang, Z., Koplev, S., Jenkins, S.L., Jagodnik, K.M., Lachmann, A., et al. (2016). Enrichr: a comprehensive gene set enrichment analysis web server 2016 update. Nucleic Acids Res. *44*, W90–W97. https://doi.org/10.1093/nar/gkw377.

39. Korotkevich, G., Sukhov, V., Budin, N., Shpak, B., Artyomov, M.N., and Sergushichev, A. (2021). Fast gene set enrichment analysis. Preprint at bioRxiv. https://doi.org/10.1101/060012.

40. Zhang, Y., Liu, T., Meyer, C.A., Eeckhoute, J., Johnson, D.S., Bernstein, B.E., Nusbaum, C., Myers, R.M., Brown, M., Li, W., et al. (2008). Model-based analysis of ChIP-Seq (MACS). Genome Biol. *9*, R137. https://doi.org/10.1186/gb-2008-9-9-r137.

41. Heinz, S., Benner, C., Spann, N., Bertolino, E., Lin, Y.C., Laslo, P., Cheng, J.X., Murre, C., Singh, H., and Glass, C.K. (2010). Simple combinations of lineage-determining transcription factors prime cis-regulatory elements required for macrophage and B cell identities. Mol. Cell *38*, 576–589. https://doi.org/10.1016/j.molcel.2010.05.004.

42. Megill, C., Martin, B., Weaver, C., Bell, S., Prins, L., Badajoz, S., McCandless, B., Pisco, A.O., Kinsella, M., Griffin, F., et al. (2021). Cellxgene: A performant, scalable exploration platform for high dimensional sparse matrices. Preprint at bioRxiv. https://doi.org/10.1101/2021.04.05.438318.

43. McLean, C.Y., Bristor, D., Hiller, M., Clarke, S.L., Schaar, B.T., Lowe, C.B., Wenger, A.M., and Bejerano, G. (2010). GREAT improves functional interpretation of cis-regulatory regions. Nat. Biotechnol. *28*, 495–501. https://doi.org/10.1038/nbt.1630.

44. McLeay, R.C., and Bailey, T.L. (2010). Motif Enrichment Analysis: a unified framework and an evaluation on ChIP data. BMC Bioinform. *11*, 165. https://doi.org/10.1186/1471-2105-11-165.

45. Thorvaldsdóttir, H., Robinson, J.T., and Mesirov, J.P. (2013). Integrative Genomics Viewer (IGV): high-performance genomics data visualization and exploration. Brief. Bioinform. *14*, 178–192. https://doi.org/10.1093/bib/bbs017.

46. Kulakovskiy, I.V., Vorontsov, I.E., Yevshin, I.S., Sharipov, R.N., Fedorova, A.D., Rumynskiy, E.I., Medvedeva, Y.A., Magana-Mora, A., Bajic, V.B., Papatsenko, D.A., et al. (2018). HOCOMOCO: towards a complete collection of transcription factor binding models for human and mouse via large-scale ChIP-Seq analysis. Nucleic Acids Res. *46*, D252–D259. https://doi.org/10.1093/nar/gkx1106.

47. Quinlan, A.R., and Hall, I.M. (2010). BEDTools: a flexible suite of utilities for comparing genomic features. Bioinformatics *26*, 841–842. https://doi.org/10.1093/bioinformatics/btq033.

48. Wolf, F.A., Angerer, P., and Theis, F.J. (2018). SCANPY: large-scale single-cell gene expression data analysis. Genome Biol. *19*, 15. https://doi.org/10.1186/s13059-017-1382-0.

49. Kaminow, B., Yunusov, D., and Dobin, A. (2021). STARsolo: accurate, fast and versatile mapping/quantification of single-cell and single-nucleus RNA-seq data. Preprint at bioRxiv. https://doi.org/10.1101/2021.05.05.442755.

50. Lun, A.T.L., Riesenfeld, S., Andrews, T., Dao, T.P., Gomes, T.; participants in the 1st Human Cell Atlas Jamboree, and Marioni, J.C. (2019). EmptyDrops: distinguishing cells from empty droplets in droplet-based single-cell RNA sequencing data. Genome Biol. *20*, 63. https://doi.org/10.1186/s13059-019-1662-y.

51. Robinson, M.D., McCarthy, D.J., and Smyth, G.K. (2010). edgeR: a Bioconductor package for differential expression analysis of digital gene expression data. Bioinformatics *26*, 139–140. https://doi.org/10.1093/bioinformatics/btp616.

52. Xie, Z., Bailey, A., Kuleshov, M.V., Clarke, D.J.B., Evangelista, J.E., Jenkins, S.L., Lachmann, A., Wojciechowicz, M.L., Kropiwnicki, E., Jagodnik, K.M., et al. (2021). Gene set knowledge discovery with Enrichr. Curr. Protoc. *1*, e90. https://doi.org/10.1002/cpz1.90.

53. Buenrostro, J.D., Wu, B., Chang, H.Y., and Greenleaf, W.J. (2015). ATAC-seq: A method for assaying chromatin accessibility genome-wide. Curr. Protoc. Mol. Biol. *109*, 21.29.1–21.29.9. https://doi.org/10.1002/0471142727.mb2129s109.

54. Feng, J., Liu, T., Qin, B., Zhang, Y., and Liu, X.S. (2012). Identifying ChIP-seq enrichment using MACS. Nat. Protoc. *7*, 1728–1740. https://doi.org/10.1038/nprot.2012.101.

55. Aughey, G.N., Estacio Gomez, A., Thomson, J., Yin, H., and Southall, T.D. (2018). CATaDa reveals global remodelling of chromatin accessibility during stem cell differentiation in vivo. eLife *7*, e32341. https://doi.org/10.7554/eLife.32341.

STAR★METHODS

KEY RESOURCES TABLE

REAGENT or RESOURCE	SOURCE	IDENTIFIER
Antibodies		
Mouse monoclonal anti-ACTA2	Thermo Fisher Scientific	Cat#MA1-06110, RRID: AB_557419
Mouse monoclonal anti-THBD (CD141), PE conjugated	BioLegend	Cat#344104, RRID: AB_2255842
Rabbit monoclonal anti-PDGFRA	Cell Signaling Technology	Cat#3174, RRID: AB_2162345
Rabbit monoclonal anti-PDGFRA, APC conjugated	BioLegend	Cat# 313511, RRID:AB_493208
Rat monoclonal anti-CD44, APC conjugated	BioLegend	Cat# 103012, RRID:AB_312963
Rat monoclonal anti-CD44	Thermo Fisher Scientific	Cat# 17-0441-82, RRID:AB_469390
Rabbit polyclonal anti-SOX9	Merck	Cat#AB5535, RRID:AB_2239761
Sheep polyclonal anti-PDPN	R&D systems	Cat#AF3670, RRID:AB_2162070
Rat monoclonal anti-E-cadherin	Thermo Fisher Scientific	Cat#13-1900, RRID:AB_2533005
Mouse monoclonal anti-CD45, PE-Cyanine7 conjugated	Thermo Fisher Scientific	Cat#25-9459-42, RRID:AB_2573544
Mouse monoclonal anti-CD31, PE-Cyanine7 conjugated	Thermo Fisher Scientific	Cat#25-0319-42, RRID:AB_10854425
Mouse monoclonal anti-CD31	Abcam	Cat# ab9498, RRID:AB_307284
Mouse monoclonal anti-CD326, PE conjugated	BioLegend	Cat#324206, RRID:AB_756080
Mouse monoclonal anti-CD326, FITC conjugated	BioLegend	Cat# 324204, RRID:AB_756078
Mouse monoclonal anti-CD36, FITC conjugated	Thermo Fisher Scientific	Cat# 11-0369-42, RRID:AB_10718972
Rabbit polyclonal anti-CD36	Proteintech	Cat# 18836-1-AP, RRID:AB_10597244
Mouse monoclonal anti-CD9, PE-Cyanine7conjugated	BioLegend	Cat# 312115, RRID:AB_2728255
Rabbit polyclonal anti-Prosurfactant protein C	Millipore	Cat# AB3786, RRID:AB_91588
Rabbit polyclonal anti-NKX2.1 (TTF1)	Millipore	Cat# 07-601, RRID:AB_310743
Rabbit monoclonal anti-AP2 gamma/ TFAP2C	Abcam	Cat# ab218107, RRID:AB_2891087
Mouse monoclonal anti-alpha-smooth muscle actin (ACTA2)	Thermo Fisher Scientific	Cat# MA1-06110, RRID:AB_557419
Rabbit monoclonal anti-ACE2	Abcam	Cat# ab108252, RRID:AB_10864415
Mouse monoclonal antii-AXIN2	R&D systems	Cat# MAB6078, RRID:AB_2044608
Rabbit monoclonal anti-P63alpha	Cell Signaling Technology	Cat# 13109, RRID:AB_2637091
Goat polyclonal anti-SOX2	R&D systems	Cat# AF2018, RRID:AB_355110
Rabbit polyclonal anti-SOX9	Millipore	Cat# AB5535, RRID:AB_2239761
Rabbit polyclonal anti-LAMP3	Atlas Antibodies	Cat# HPA051467, RRID:AB_2681495
Mouse Monoclonal anti-HT2-280	Terrace biotech	Cat# TB-27AHT2-280, RRID:AB_2832931
Rabbit polyclonal anti-matureSFTPC	Seven Hills Bioreagents	Cat# WMAB-76694, RRID:N/A
Rabbit polyclonal anti-proSFTPC	Millipore	Cat# AB3786, RRID:AB_91588
Rabbit polyclonal anti-matureSFTPB	Seven Hills Bioreagents	Cat# WMAB-48604, RRID:N/A
Rabbit polyclonal anti-proSFTPB	Seven Hills Bioreagents	Cat# WMAB-55522, RRID:N/A
Mouse monoclonal anti-ABCA3	Seven Hills Bioreagents	Cat# WMAB-17G524, RRID:N/A
Rabbit polyclonal anti-ZO-1	Thermo Fisher Scientific	Cat# 40-2200, RRID:AB_2533456

(*Continued on next page*)

Continued

REAGENT or RESOURCE	SOURCE	IDENTIFIER
Rabbit monoclonal anti-GATA6	Cell Signaling Technology	Cat# 5851, RRID:AB_10705521
Rabbit polyclonal anti-NOTUM	Novus Biological	Cat# NBP2-94699, RRID:N/A
Mouse monoclonal anti-KI67	BD Biosciences	Cat# 550609, RRID:AB_393778
Mouse monoclonal anti-GAPDH	Abcam	Cat# ab8245, RRID:AB_2107448
Normal Rabbit IgG	Cell Signaling Technology	Cat# 2729, RRID:AB_1031062
Biological samples		
Organoid lines: HDBR 13393, 13567, 14387, 14404, 14459, 14556, 14598, 14630, 14643, 14644, 14906, 14996, 14998	HDBR London and Newcastle	N/A
Organoid lines: BRC 1915, 1938, 1943	Brain Repair Center, University of Cambridge	N/A
Chemicals, peptides, and recombinant proteins		
ActinGreen™ 488 ReadyProbes™ Reagent	Thermo Fisher Scientific	Cat# R37110
2,2′-Thiodiethanol (TDE)	Merck	Cat# 166782
ABC99	Cambridge Bioscience	Cat# CAY25858
ActivX™ TAMRA-FP Serine Hydrolase Probe	Thermo Fisher Scientific	Cat# 88318
M-PER™ Mammalian Protein Extraction Reagent	Thermo Fisher Scientific	Cat# 78503
Halt™ Phosphatase Inhibitor Cocktail	Thermo Fisher Scientific	Cat# 78420
N2 supplement	Thermo Fisher Scientific	Cat#17502001
B27 supplement	Thermo Fisher Scientific	Cat#12587001
N-acetylcysteine	Merck	Cat#A9165
EGF	PeproTech	Cat#AF-100-15
FGF10	PeproTech	Cat#100-26
FGF7	PeproTech	Cat#100-19
Noggin	PeproTech	Cat#120-10C
R-spondin	Stem Cell Institute, University of Cambridge	N/A
CHIR99021	Stem Cell Institute, University of Cambridge	N/A
SB431542	Bio-techne	Cat# 1614
A83-01	Tocris	Cat# 2939
BMP4	Peprotech	Cat# 120-05
TGF-β1	Peprotech	Cat# 100-21
8-Bromoadenosine 3'5'-cyclic monophosphate (cAMP)	Merck	Cat# B5386
3-Isobutyl-1-methylxanthine (IBMX)	Merck	Cat# I5879
Y-27632	Merck	Cat# 688000
Dexamethasone (Dex)	Merck	Cat# D4902
DAPT	Merck	Cat# D5942
Doxycycline (Dox)	Merck	Cat# D9891
Trimethoprim (TMP)	Merck	Cat# 92131
Collagenase	Merck	Cat# C9891
Dispase	Thermo Fisher Scientific	Cat# 17105041
DNase	Merck	Cat# D4527
RBC lysis buffer	BioLegend	Cat# 420301
Cell Recovery Solution	Corning	Cat# 354253
Collagen	Merck	Cat# CLS3493
Micrococcal Nuclease (MNase)	Cell Signaling Technology	Cat# 10011S
RIPA buffer	Merck	Cat# R0278

(*Continued on next page*)

Continued		
REAGENT or RESOURCE	SOURCE	IDENTIFIER
Critical commercial assays		
CD326 (EpCAM) MicroBeads, human	Miltenyi Biotec	Cat# 130-061-101
In-Fusion® HD Cloning Plus	Takara	Cat# 638909
RNeasy Mini Kit	Qiagen	Cat# 74004
PicoPure™ RNA Isolation Kit	Thermo Fisher Scientific	Cat# KIT0204
SimpleChIP® Chromatin immunoprecipitation kit	Cell Signaling Technology	Cat# 9002
Chromium Single Cell V(D)J Kits (v1)	10X	N/A
Deposited data		
Bulk RNA-seq, ATAC-seq, DamID-seq of organoids	NCBI's GEO	GSE178529
scRNA-seq of human fetal lungs	ArrayExpress[7]	E-MTAB-11278
scRNA-seq of lung organoids	ArrayExpress	E-MTAB-11435
Oligonucleotides		
gRNA-NKX2.1_1: 5'-GTCTGACGGCGGCAGAAGAG-3'	Horlbeck et al.[34]	N/A
gRNA-NKX2.1_2: 5'-GGACCAACAGTGCGGCCCCA-3'	Horlbeck et al.[34]	N/A
gRNA-NKX2.1_2: 5'-GAAATGAGCGAGCGAGTCTG-3'	Horlbeck et al.[34]	N/A
gRNA-TFAP2C_1: 5'-GGCGGTCTTGACACTCGCGG-3'	Horlbeck et al.[34]	N/A
gRNA-TFAP2C_2: 5'-GTCGCCAGGACACACTGTTC-3'	Horlbeck et al.[34]	N/A
gRNA-TFAP2C_3: 5'-GGTCACTGGACACGCATCGG-3'	Horlbeck et al.[34]	N/A
Recombinant DNA		
pLenti-hSPC-eGFP-EF1a-TagRFP	This paper	N/A
pLenti-tetON-KRAB-dCas9-DHFR-EF1a-TagRFP-2A-tet3G	Sun et al.[5]	Addgene: #167935, RRID:Addgene_167935
pLenti-U6-gRNA-EF1a-EGFP-CAAX	Sun et al.[5]	Addgene: #167936, RRID:Addgene_167936
pLenti-tetON-NKX2-1-EF1a-TagRFP-2A-tet3G	Sun et al.[5]	Addgene: #167942; RRID:Addgene_167942
pLenti-tetON-NKX2-1-delDBD-EF1a-TagRFP-2A-tet3G	This paper	N/A
pLenti-tetON-NKX2-1-R162del-EF1a-TagRFP-2A-tet3G	This paper	N/A
pLenti-tetON-NKX2-1-Q175*-EF1a-TagRFP-2A-tet3G	This paper	N/A
pLenti-tetON-NKX2-1-R178*-EF1a-TagRFP-2A-tet3G	This paper	N/A
pLenti-tetON-NKX2-1-I207F -EF1a-TagRFP-2A-tet3G	This paper	N/A
pLenti-tetON-NKX2-1-I207M -EF1a-TagRFP-2A-tet3G	This paper	N/A
pLenti-SFFV-mNG-Dam-NKX2.1	This paper	N/A
pLenti-tetON-TFAP2C-EF1a-TagRFP-2A-tet3G	This paper	N/A
pLenti-tetON-NOTUM-EF1a-TagRFP-2A-tet3G	This paper	N/A

(*Continued on next page*)

Continued

REAGENT or RESOURCE	SOURCE	IDENTIFIER
pLenti-tetON-GATA6-EF1a-TagRFP-2A-tet3G	This paper	N/A
**Software and algorithms**		
ImageJ (version: 2.1.0)	Schneider et al.[35]	https://imagej.nih.gov/ij/; RRID:SCR_003070
GraphPad Prism software (version: 9.1.0)	GraphPad Prism	GraphPad Prism (https://graphpad.com); RRID:SCR_015807
FlowJo software (version: 10.0.0)	FlowJo	FlowJo (https://www.flowjo.com/); RRID:SCR_008520
EdgeR (version 3.16.5)	Choi et al.[36]	https://bioconductor.org/packages/release/bioc/html/edgeR.html
DAVID	da Huang et al.[37]	https://david.ncifcrf.gov/
Enrichr	Kuleshov et al.[38]	https://maayanlab.cloud/Enrichr/
fgsea	Korotkevich et al.[39]	https://montilab.github.io/hypeR-docs/articles/docs/fgsea.html
MACS2 (version: 2.1.2)	Zhang et al.[40]	https://github.com/macs3-project/MACS
HOMER (version: 4.11)	Heinz et al.[41]	http://homer.ucsd.edu/homer/
Cellxgene (version: 0.16.7)	Megill et al.[42]	https://github.com/chanzuckerberg/cellxgene
GREAT	McLean et al.[43]	https://github.com/bgruening/galaxytools/issues/184
MEME	McLeayand Bailey[44]	https://meme-suite.org/meme/doc/meme-chip.html?man_type=web
IGV (version: 2.4.19)	Thorvaldsdóttir et al.[45]	https://igv.org/
HOCOMOCO Human (version: 11)	Kulakovskiy et al.[46]	https://hocomoco11.autosome.org/
Bedtools (version: 2.26.0)	Quinlan and Hall[47]	https://github.com/arq5x/bedtools2/releases?after=v2.26.0
Scanpy (version: 1.8.2)	Wolf et al.[48]	https://github.com/scverse/scanpy
STARsolo (version: 2.7.3a)	Kaminow et al.[49]	https://github.com/alexdobin/STAR/blob/master/docs/STARsolo.md
EmptyDrop	Lun et al.[50]	https://github.com/MarioniLab/DropletUtils
**Other**		
SH800S Cell Sorter	Sony Biotechnology	N/A
BD Influx™ Cell Sorter	BD biosciences	N/A

RESOURCE AVAILABILITY

Lead contact

Lead contact Further information and requests for resources and reagents should be directed to and will be fulfilled by the lead contact, Emma L. Rawlins (e.rawlins@gurdon.cam.ac.uk).

Materials availability

Lung organoid lines used in this study are available from the lead contact, Emma L. Rawlins (e.rawlins@gurdon.cam.ac.uk), with a completed Materials Transfer Agreement.

Data and code availability

- Sequencing data have been deposited at ArrayExpress and GEO and are publicly available. Accession numbers are listed in the key resources table. Processed single cell sequencing data reported in this paper are available at https://fetal-lung.cellgeni.sanger.ac.uk/.
- This paper does not report original code.
- Any additional information required to reanalyse the data reported in this paper is available from the lead contact upon request.

EXPERIMENTAL MODEL AND SUBJECT DETAILS

Human embryonic and fetal lung tissue

Human embryonic and fetal lung tissues were provided from terminations of pregnancy from Cambridge University Hospitals NHS Foundation Trust under permission from NHS Research Ethical Committee (96/085) and the MRC/Wellcome Trust Human Developmental Biology Resource (London and Newcastle, University College London (UCL) site REC reference: 18/LO/0822; Newcastle site REC reference: 18/NE/0290; Project 200454; www.hdbr.org). Sample age ranged from 4 to 23 weeks of gestation (post-conception weeks; pcw). Stages of the samples were determined according to their external physical appearance and measurements. All the samples used for the current study had no known genetic abnormalities. Sample gender was unknown at the time of collection and was not determined.

METHOD DETAILS

In vitro culture of human fetal lung organoids

The isolated tip epithelial cells were embedded in Matrigel (Corning, 356231) and cultured in 48-well plates in self-renewing (SN) medium: Advanced DMEM/F12 supplemented with 1x GlutaMax, 1 mM HEPES and Penicillin/Streptomycin, 1X B27 supplement (without Vitamin A), 1X N2 supplement, 1.25 mM n-Acetylcysteine, 50 ng/ml recombinant human EGF (PeproTech, AF-100-15), 100 ng/ml recombinant human Noggin (PeproTech, 120-10C), 100 ng/ml recombinant human FGF10 (PeproTech, 100-26), 100 ng/ml recombinant human FGF7 (PeproTech, 100-19), 3 μM CHIR99021 (Stem Cell Institute, University of Cambridge) and 10 μM SB431542 (Bio-Techne, 1614). The culture medium was replaced every 2 days and the organoids were usually split 1:3 once per week by breaking them into small fragments. Numbers of replicates are indicated in figure legends. To activate dual SMAD signalling (Figure S4B),10 ng/μl recombinant human BMP4 (Peprotech, 120-05) and 10 ng/μl recombinant human TGF-β1 (Peprotech, 100-21) were added to the medium instead of SB431542 and Noggin.

To perform *in vitro* co-culture experiments, freshly sorted 2x10^5 PDGFRA$^+$CD141$^+$ myofibroblasts or PDGFRA$^-$CD141$^-$ fibroblasts were mixed with *SFTPC*-eGFP$^+$ Lin^{POS} organoids in 100 μl Matrigel and then loaded into an insert of transwell (Merck, CLS3493). On the bottom well plates, coated with Collagen (Merck, CLS3493), 4 x 10^5 PDGFRA$^-$CD141$^-$ fibroblasts were plated in the culture medium containing 2% fetal bovine serum (FBS; Thermo Fisher Scientific, 10500064) in the Advanced DMEM/F12 supplemented with 1x GlutaMax, 1 mM HEPES and Penicillin/Streptomycin. 100 μl culture medium was added every 2 days. For coculture of PDGFRA$^+$CD141$^+$ myofibroblasts with PDGFRA$^-$CD141$^-$ fibroblasts, 2x10^5 of PDGFRA$^+$CD141$^+$ myofibroblasts were plated on the insert and 4x10^5 of PDGFRA$^-$CD141$^-$ fibroblasts were plated on the bottom well plate. After 2 weeks of co-cultures the organoids, or the mesenchymal cells, were harvested for further analysis.

Isolation of tip epithelial cells, myofibroblasts, and alveolar fibroblasts

For isolation of CD44$^+$, or CD44$^+$CD36$^+$, tip epithelium directly from the distal lung tissues, the tissues were finely dissected into tiny pieces and enzymatically digested into single cells by incubating in a dissociation solution containing 0.125 mg/ml Collagenase (Merck, C9891), 1 U/ml Dispase (Thermo Fisher Scientific, 17105041) and 0.1 U/μl DNase (Merck, D4527), in a rotating incubator for 1 hour at 37°C. After rinsing in washing buffer containing 2% FBS in cold PBS the cells were filtered through a 100 μm strainer and harvested by centrifugation. The cell pellets were resuspended and treated with RBC lysis buffer (BioLegend, 420301). Next, the cells were rinsed in the washing buffer and then incubated with primary antibodies against CD45 (1:100; PE-Cy7 conjugated, Thermo Fisher Scientific, 25-9459-42), CD31 (1:100; PE-Cy7 conjugated, Thermo Fisher Scientific, 25-0319-42), EPCAM (1:100; PE-conjugated; BioLegend, 324206), CD44 (1:200; APC-conjugated; BioLegend, 103012), and CD36 (1:100; FITC, conjugated; Thermo Fisher Scientific, 11-0369-42), with a viability dye, Zombie (Biolegend, 423113) for 25 min on ice. Following removal of dead cells and immune/endothelial cells, the EPCAM+ epithelial cells were sorted by CD44 and/or CD36 expression by FACS (BD Influx™ Cell Sorter) (Figure 1G).

Alternatively, the tip epithelial cells were isolated by EPCAM$^+$ magnetic-activated cell sorting (MACS) beads according to the manufacturer's instruction (CD326 MicroBeads, human, Miltenyi Biotec) from the distal lung tissues. Then, the enriched EPCAM$^+$ epithelial cells were sorted by CD44 and/or CD36 expression by FACS (SH800S Cell Sorter) to more purely enrich the tip cell population (Figure 2A).

To purify myofibroblasts and alveolar fibroblasts, the single cells dissociated from the distal lung tissues were incubated with the following primary antibodies: CD45 (1:100; PE-Cy7 conjugated, Thermo Fisher Scientific, 25-9459-42), CD31 (1:100; PE-Cy7 conjugated, Thermo Fisher Scientific, 25-0319-42), CD9 (1:100; PE-Cy7 conjugated, BioLegend, 312115), EPCAM (1:100; FITC-conjugated, BioLegend, 324204), PDGFRA (1:100; APC-conjugated, BioLegend, 313511), CD141 (1:100; PE-conjugated, BioLegend, 344104), with the viability dye, Zombie (Biolegend, 423113). After removing dead cells, immune/endothelial cells, airway smooth muscle cells, and epithelial cells, the cells are sorted by PDGFRA and/or CD141 expression using BD Influx Cell Sorter. The sorted cells were directly applied to an organoid coculture or a gene expression analysis.

Alveolar differentiation of Lin^{POS} organoids

The Lin^{POS} organoids were embedded in Matrigel and cultured for 7 days in alveolar type 2 (AT2) differentiation medium: Advanced DMEM/F12 supplemented with 1x GlutaMax, 1 mM HEPES and Penicillin/Streptomycin, 1X B27 supplement (without Vitamin A), 1x

N2 supplement, 1.25 mM n-Acetylcysteine, 10 mM CHIR99021, 50 μM Dexamethasone (Merck, D4902), 0.1 M 8-Bromoadenosine 3'5'-cyclic monophosphate (cAMP; Merck, B5386), 0.1 M 3-Isobutyl-1-methylxanthine (IBMX; Merck, 15679), 50 mM DAPT (Merck, D5942) with 10 mM SB431542 or 10 mM A83-01 (Tocris, 2939). The culture medium was replaced every 2 days without passaging.

Lentiviral transduction

To introduce a reporter system into the tip epithelial cells, the lentiviral vector pHAGE hSPC-eGFP-W given from Darrell Kotton (Addgene plasmid # 36450; http://n2t.net/addgene:36450; RRID: Addgene_36450) was modified by inserting EF1a-promoter TagRFP cassette. The tip epithelial cells were infected with the modified lentiviral vector for 24 hours at 37°C in a single cell suspension in the SN medium containing 10 μM Y-27632 (Merck, 688000). After 24 hours, the cells were embedded to the Matrigel and cultured in the SN medium containing 10 μM Y-27632 for another 48 hours to support single cell survival. The cultured cells were further sorted by eGFP/TagRFP signals to enrich the infected cells.

For overexpressing NKX2.1 and/or TFAP2C, Tet-ON 3G doxycycline (Dox)-inducible lentiviral vector (Takara, 631337) was modified by inserting EF1a-TagRFP-2A-tet3G with tetON-NKX2-1 CDS, or by inserting EF1a-mNeonGreen-2A-tet3G with tetON-TFAP2C CDS. For generating NKX2.1 variants, naturally occurring mutations in NKX2.1 binding domain region was selected from Leiden Open Variation Database 3.0 [22] (www.lovd.nl/3.0) and two previously reported clinical cases[23,24] – 1 amino acid deletion[22] (p.R162del), two nonsense point mutations[22] (p.Q175* and p.R178*), and two missense point mutations[23,24] (p.I207F and p.I207M). NKX2.1 CDS harbouring each mutation was amplified and inserted by Infusion (638909, Takara) cloning into the tetON-NKX2.1/EF1a-TagRFP-2A-tet3G Dox-inducible lentiviral vector. NKX2.1 CDS lacking the entire DNA binding domain was inserted into the EF1a-TagRFP-2A-tet3G Dox-inducible lentiviral vector by Infusion cloning.

For the NKX2.1 or TFAP2C knock-down experiment, a modified Dox-inducible CRISPRi vector was gifted;[5] N-terminal KRAB-dCas9 (a gift from Bruce Conklin, Addgene plasmid # 73498) fused with a destabilising domain, dihydrofolate reductase (DHFR) sequence that is only stabilised by trimethoprim (TMP) treatment, was sub-cloned into the EF1a-TagRFP-2A-tet3G Dox-inducible lentiviral vector.[5] Treatment of 2 μg/ml Dox (Merck, D9891) with 10 nmol/L TMP (Merck, 92131) in the SN medium stabilizes the functional KRAB-dCas9 protein. Three gRNAs targeting NKX2.1[34] were individually subcloned into gRNA lentivirus as follows: gRNA-1; 5'-GTCTGACGGCGGCAGAAGAG-3', gRNA-2; 5'-GGACCAACAGTGCGGCCCCA-3', gRNA-3; 5'- GAAATGAGCGAGCGAGTCTG-3'. The gRNAs for targeting TFAP2C: gRNA-1; 5'-GGCGGTCTTGACACTCGCGG-3', gRNA-2; 5'- GTCGCCAGGACACACTGTTC-3', gRNA-3; 5'- GGTCACTGGACACGCATCGG-3'.

Single cells dissociated from the organoids were infected and the infected cells were sorted by TagRFP and/or mNeonGreen fluorescent signal using FACS (Figure 5) after 48 hours of infection. The sorted TagRFP$^+$ and/or mNeonGreen$^+$ cells were cultured in the Matrigel in the absence of Dox or TMP for 1 week. After the cells were grown into a typical organoid, the Dox and/or TMP were added and culture continued for additional 2 weeks.

For transducing the N-terminal Dam-NKX2.1 fusion protein to the organoids, NKX2.1 CDS was inserted into the SFFV-mNeonGreen-Dam lentiviral vector by infusion cloning (see Table S4 for vector map). 200,000 CD44$^+$ cells sorted from the 7-9 pcw organoids, CD36$^+$/CD44$^+$ cells sorted from the Lin^{POS} organoids, or single-cell dissociated alveolar organoids were infected with the lentivirus harbouring mNeoGreen-Dam only or mNeoGreen-Dam-NKX2.1 for 2 days.

Immunostaining of organoids and lung tissues

For immunostaining of human lung tissue sections, the lungs were fixed in 4% paraformaldedyde (PFA; Merck, 158127) overnight, washed in PBS and 15%, 20% and 30% sucrose (w/v) in PBS before embedding in Optimum Cutting Temperature (OCT) medium (Merck, F4680). 12 μm thick frozen sections were collected and permeabilised using 0.3% Triton-X in PBS for 15 min. Antigen retrieval was performed by heating the slides in 10 mM Na-Citrate buffer at pH 6.0 in a microwave for 5 min. Then slides were treated with blocking solution containing 5% NDS, 1% Bovine Serum Albumin (BSA), 0.1% Triton-X in PBS at room temperature for 1 hour.

For whole-mount immunostaining of lung organoids, the Matrigel was completely removed from the cultured organoids using Cell Recovery Solution (Corning, 354253) followed by fixation in 4% PFA for 30 min on ice. After rinsing in PBS washing solution containing 0.2% (v/v) Triton X-100 and 0.5% (w/v) BSA, the samples were transferred to a round-bottom 96 well plate and incubated in permeabilization/blocking solution containing 0.2% (v/v) Triton X-100, 1% (w/v) BSA, and 5% normal donkey serum (NDS) in PBS, overnight at 4°C. For primary antibody treatment, the following antibodies were treated to the organoids and the tissue slices at 4°C overnight: proSFTPC (1:200; Merck, AB3786), E-cadherin (1: 500; Thermo Fisher Scientific, 13-1900), NKX2.1 (1:200; Millipore, 07-601), TFAP2C (1:200; Abcam, ab218107), CD44 (1:200; Thermo Fisher Scientific, 17-0441-82), CD36 (1:200; Proteintech, 18836-1-AP), alpha-smooth muscle actin (1:500; Thermo Fisher Scientific, MA1-06110), ACE2 (1:100; Abcam, ab108252), AXIN2 (1:200; R&D Systems, MAB6078), PDPN (1:200; R&D Systems, AF3670), CD31 (1:200; Abcam, ab9498), PDGFRA (1:200; Cell Signaling Technology, 3174), TP63 (1:200; Cell Signaling Technology, 13109), SOX2 (1: 500, R&D systems, AF2018), SOX9 (1: 500, Merck, AB5535), LAMP3 (1:100; Atlas Antibodies, HPA051467), HTII-280 (1:200; Terrace Biotech, TB-27AHT2-280), mature SFTPC (1:200; Seven Hills Bioreagents, WRAB-76694), proSFTPB (1:200; Seven Hills Bioreagents, WRAB-55522), ABCA3 (1:200; Seven Hills Bioreagents, WMAB-17G524), CD141 (1:100; PE-conjugated; BioLegend, 344104), ZO-1 (1:200; Thermo Fisher Scientific, 40-2200), GATA6 (1:200; Cell Signaling Technology, 5851), NOTUM (1:1000; Novus Biologicals, NBP2-94699), and KI67 (1:200; BD Biosciences, 550609). F-actin staining reagent (1 droplet in 200 μl; Thermo Fisher Scientific, R37110) was treated at room temperature for 30 min, followed by nuclei counter staining. After three washes with PBS, 97% (v/v) 2'−2'-thio-diethanol (TDE, Merck, 166782) was treated for clearing. Images were collected under Leica SP8 confocal microscope.

In situ hybridization chain reaction (*in situ* HCR)

In situ HCR v3.0 was performed according to the manufacturer's procedure (Molecular Instruments[36]). Probes were designed according to the protocol and amplifiers with buffers were purchased from Molecular Technologies. Sequence information of the probes for detecting *SFTPC*, *WNT2, NOTUM, WIF1, AXIN2, SOX9, FGFR4, TPPP3, SCGB3A2, SPOCK2*, and *TFAP2C* mRNA targets is listed in Table S3. Briefly, frozen human tissue sections were cut from 20 μm up to 100 μm from lungs fixed overnight in 4% PFA in DEPC-treated PBS and processed to cryoblocks. Lung sections were carefully rinsed in nuclease-free water, followed by 10 μg/mL proteinase K treatment (Thermo Fisher Scientific, AM2546), and 2 pmol of each probe was treated at 37°C overnight. After washing, the tissue was incubated with 6 pmol of the amplifiers at room temperature overnight for amplification. The amplifiers, consisting of a pair of hairpins conjugated to fluorophores, Alexa 546, 647, or 488, were snap-cooled separately and added at final 0.03 μM to the tissue. After removing excess hairpins in 5X SSC (sodium chloride sodium citrate) buffer (5X SSCT), containing 0.1% Triton X-100, nuclei were counter-stained with DAPI.

To combine *in situ* HCR with antibody immunostaining, the frozen human tissue sections from 20 μm up to 100 μm thickness were permeabilised using 0.3% Triton-X in DEPC-treated PBS for 3 min at room temperature. The lung sections were treated with probes at 37°C overnight. After washing in 5X SSCT, incubated with 6 pmol of the amplifiers at room temperature overnight for amplification. After washing three times in 5X SSCT, the tissues were treated with blocking solution containing 5% NDS, 1% BSA, 0.1% Triton-X in DEPC-treated PBS at room temperature for 1 hour and directly treated with a primary antibody against ACTA2 (1:500; Thermo Fisher Scientific, MA1-06110) or E-cadherin (1:200; Thermo Fisher Scientific, 13-1900) overnight at 4°C, followed by a secondary antibody treatment (1:500; Thermo Fisher Scientific, A10036) at room temperature for 3 hours. After washing three times in 5X SSCT, nuclei were stained with DAPI. Finally, the tissues were processed to 2'-2'-thio-diethanol (TDE, Sigma, 166782) for clearing and mounting: 10 %, 25 %, 50 % (v/v) TDE in 1x DEPC-treated PBS for 1 hour and 97% TDE overnight at 4°C. Images were collected under Leica SP8 confocal microscope.

RNA extraction, cDNA synthesis, qRT-PCR analysis, and bulk RNA-sequencing

Organoids were removed from the Matrigel and lysed. Total RNA was extracted according to the RNeasy Mini Kit (Qiagen, 74004) protocol. For cells freshly purified from human lung tissues were directly lysed using 100 μl lysis buffer from PicoPure™ RNA Isolation Kit (Thermo Fisher Scientific, KIT0204). First Strand cDNA synthesis was performed using High-Capacity cDNA Reverse Transcription Kit (Applied Biosystems, 4368814). Then, cDNA was diluted 1:50 for qRT-PCR reaction (SYBR Green PCR Master Mix; Applied Biosystems, 4309155). Primer sequence information is listed in Table S3. Data is presented as fold change, calculated by ddCt method, using *ACTB* as housekeeping reference gene. For bulk RNA-seq, RNA quality was validated on Agilent 2200 Tapestation. The RNA-seq libraries were generated at the Cancer Research UK Cambridge Institute and sequenced on an Illumina HiSeq 4000. A list of differentially expressed genes was extracted using the counted reads and R package edgeR[51] version 3.16.5 for the 3 pairwise comparisons (Table S1). GO biological processes term enrichment, KEGG pathway, and gene set enrichment analysis were performed using DAVID,[37] Enrichr,[52] and R package fgsea package,[39] respectively.

Immunoblotting

The organoid samples were harvested and lysed (RIPA buffer; Merck, R0278) after complete removal of the Matrigel and run on 12.5 % ~ 20 % SDS PAGE gels. Proteins on the gels were transferred onto PVDF membrane using BioRad Mini Trans-Blot system (BioRad, Mini Trans-Blot® Cell). The membranes were washed with pure water and blocked with 5% skimmed milk in 0.1% Tween-20/PBS (PBST) for 30 min at room temperature. Membranes were incubated with primary antibodies against NKX2.1 (1:200; Millipore, 07-601), proSFTPC (1:1000; Millipore, AB3786), mature SFTPC (1:1000; Seven Hills Bioreagents, WRAB-76694), mature SFTPB (1:1000; Seven Hills Bioreagents, WRAB-48604), NOTUM (1:1000; Novus Biologicals, NBP2-94699), and GAPDH (1:5000; Abcam, ab8245) in the blocking buffer overnight at 4 °C. After washing with PBST, secondary antibodies conjugated with fluorescence dyes (1:5000; anti-mouse IRDye® 800CW and anti-rabbit IRDye® 680RD; Abcam, ab216774 and ab216779, respectively) were treated at room temperature for 3 hours. The membranes were washed in PBST and developed using Li-Cor Odyssey imaging system.

Activity-based protein profiling on SDS-PAGE gel

In situ gel-based activity-based protein profiling was performed according to the manufacturer's instruction (ActivX™ TAMRA-FP Serine Hydrolase Probe, Thermo Fisher Scientific, 88318). 1 x 10^6 of freshly isolated alveolar fibroblasts (PDGFRA$^-$CD141$^-$) and myofibroblasts (PDGFRA$^-$CD141$^-$) from late-stage lung tissues were lysed (Thermo Fisher Scientific, 78503), in the presence of a phosphatase inhibitor cocktail (1:100; Thermo Fisher Scientific, 78420), on ice for 10 min. 10 μg of lysate at 2 mg/mL concentration was treated with 1μM ABC99 (Cambridge Bioscience, CAY25858), a selective, irreversible NOTOM inhibitor, or DMSO only for 30 min at 37°C. 2 μM of the serine hydrolase FP probe was incubated for 30 min at room temperature. The reactions stopped by adding 4X Laemmli reducing sample buffer (BioRad, 1610747) and boiling for 5 minutes. The labelled proteins were loaded by SDS-PAGE, followed by fluorescent gel imaging.

Chromatin immunoprecipitation

Chromatin immunoprecipitation (ChIP) was performed according to SimpleChIP® Chromatin immunoprecipitation protocol (Cell Signaling Technology, 9002). In brief, the organoids were harvested and enzymatically dissociated into single cells using TrypLE

Express Enzyme (Thermo Fisher Scientific, 12605010). Then the cells were crosslinked with 1% formaldehyde for 15 min at room temperature and the reaction was quenched by glycine at a final concentration of 0.125 M. Chromatin was digested with 1 μl MNase (Cell Signaling Technologies, 10011S) for 20 min at 37°C, followed by sonication for 12 cycles of 30 seconds on and 30 seconds off using Biorupter (Diagenode, UCD-300), to length of an average size of 150-900 bp. 5 μg of digested chromatin samples was treated with antibodies against rabbit IgG (1:100; Cell Signaling Technology, 2729) or NKX2.1 (1:100; Millipore, 07-601). The amount of immunoprecipitated DNA was quantified by qPCR using primers specific for promoter regions of SFTPC, LAMP3, and SLC34A2. Fold enrichment values are presented as the fold-change over the level of ChIP with negative control IgG antibody (ChIP signal/IgG signal). Sequence information of the primers for targeting SFTPC, LAMP3, and SLC34A2 promoter regions is listed in Table S3.

Bulk ATAC-sequencing

Genome-wide chromatin accessibility of lung organoids was assessed as previously described.[53] In brief, 50,000 cells were harvested from organoids and lysed in lysis buffer (10 mM Tris-HCl, pH 7.4, 10 mM NaCl, 3 mM MgCl2, 0.1% (v/v) IGEPAL CA-630). The lysate was treated in 50 μL reactions with Nextera TDE1 transposase (Illumina, 15027865) for 30 min at 37°C. The purified DNA was amplified and indexed using Nextra DNA CD Indexes (Illumina, 20018707), and size distribution of the DNA libraries was analysed using High-sensitivity Qubit dsDNA Assay Kit (ThermoFisher, Q32851) and Agilent 2200 Tapestation. The libraries were sequenced on an Illumina HiSeq 4000. Peak calling was done using MACS2 algorithm[54] (version 2.1.1) and further processed to extract differential peaks (Table S2). Then, the differential peak data was further used for analysing transcription factor motifs using HOMER[41] software in combined with RNA-seq data.

Electron microscopy imaging

The organoid samples were fixed in 2 % formaldehyde/2 % glutaraldehyde in 0.05 M sodium cacodylate buffer (NaCAC), pH 7.4, containing 2 mM calcium chloride (Merck, C27902) overnight at 4°C. After washing in 0.05 M NaCAC at pH 7.4, the samples were osmicated for 3 days at 4°C. After washing in deionised water (DIW), the samples were treated twice with 0.1 % (w/v) thiocarbohydrazide (Merck, 223220) in DIW for each 20 min and 1 hour at room temperature in the dark, followed by block-staining with uranyl acetate (2 % uranyl acetate in 0.05 M maleate buffer pH 5.5) for 3 days at 4°C. Then, the samples were dehydrated in a graded series of ethanol (50%/70%/95%/100%/100% dry) 100% dry acetone and 100% dry acetonitrile, three times in each for at least 5 min. Next, the samples were infiltrated with a 50:50 mixture of 100% dry acetonitrile/Quetol resin (TAAB, Q005) without BDMA (TAAB, B008) overnight, followed by 3 days in 100% Quetol without BDMA. The sample was infiltrated for 5 days in 100% Quetol resin with BDMA, exchanging the resin each day. The Quetol resin mixture is: 12 g Quetol 651, 15.7 g NSA (TAAB, N020), 5.7 g MNA (TAAB, M012) and 0.5 g BDMA. Samples were placed in embedding moulds and cured at 60°C for 3 days.

Thin sections were cut using an Ultracut E ultramicrotome (Leica) and mounted on melinex plastic coverslips. The coverslips were mounted on aluminium SEM stubs using conductive carbon tabs and the edges of the slides were painted with conductive silver paint. Then, the samples were sputter coated with 30 nm carbon using a Quorum Q150 T E carbon coater and imaged in a Verios 460 scanning electron microscope (FEI, Thermo Fisher Scientific) at 4 keV accelerating voltage and 0.2 nA probe current in backscatter mode using the concentric backscatter detector in immersion mode at a working distance of 3.5-4 mm; 1,536 x 1,024 pixel resolution, 3 μs dwell time, 4 line integrations. Stitched maps were acquired using FEI MAPS software using the default stitching profile and 10% image overlap.

Organoid single-cell RNA sequencing

Four biological replicates of the Lin^{POS} and alveolar organoids were harvested individually and enzymatically dissociated into single cells using TrypLE Express Enzyme. The cell suspension was passed through a 30 μm filter, pelleted, and resuspended in appropriate volume with 0.04% BSA/PBS, and single cell RNA seq was carried out according to 10X Chromium Single Cell 5' Kits (v1). Library generation for 10x Genomics analysis were performed following the Chromium Single Cell 5′ Reagents Kits (10x Genomics) and sequenced on a NovaSeq 6000 S4 Flowcell (paired-end (PE), 150-bp reads) to achieve an average of 50,000 PE reads per cell. The single-cell RNA seq data were mapped (STARsolo[49] v2.7.3a) to GRCh38 reference (v3.0.0; Ensembl 93), followed by cell calling post-processed with an implementation of EmptyDrops extracted from Cell Ranger 3.0.2.

Targeted DamID sequencing

For Targeted DamID sequencing, genomic DNA was extracted from whole organoids or sorted cells and processed as previously described.[20] Sequencing was performed as single end 100 bp reads by the Gurdon Institute NGS core facility using an illumina NovaSeq 6000[19]. Raw fastq files were analysed with a modified version of the damidseq_pipeline. Reads were mapped to a bowtie2 indexed GRCh38 genome assembly (hg38), binned into 5'-GATC-3' fragments and each NKX2-1 fusion protein sample was normalised against a separate Dam-only replicate. Each organoid sample was treated as an individual replicate. For each of these replicates, the NKX2-1 fusion samples were normalized against a paired Dam-only control derived from the same biological sample (RPM normalization, 300 bp bins). Binding intensity values were quantile normalized across all replicates for each stage and back-transformed ("unlog"). Dam-only samples were analysed in parallel without normalization as a proxy for chromatin accessibility.[55] For visualisation, files were converted to bigwig format using bedGraphToBigWig (v4) and imported into the Integrative Genomics Viewer (IGV v2.4.19). Macs2 (v2.1.2) was used to call broad peaks for each NKX2-1 fusion and Dam-only pair using the bam files generated by the damidseq_pipeline. Peaks were filtered to only those present in 2/3 replicates using bedtools (v2.26.0) and

surpassing FDR<10^{-5} (Table S4). Distribution across genomic features was analysed and peak-gene associations were identified using GREAT.[43] Binding intensities from genes associated with peaks were extracted from bedgraph files and averaged across gene bodies, including -1kb from the TSS. In order to derive binding patterns across time points, these genes were subjected to k-means clustering, with parameters optimised according to silhouettes calculated with R-cluster package (v2.0.7-1). Heatmaps were generated using the ggplot2 R package. *De novo* motif enrichment analysis was performed using AME package from MEME suite[44] with the HOCOMOCO Human (v11) motif database as reference.

QUANTIFICATION AND STATISTICAL ANALYSIS

Data are expressed as average ± standard deviation (SD). Statistical significance was evaluated by unpaired student's *t* test, 1- or 2-way ANOVA with Tukey/Bonferroni/Dunnett comparison multiple comparison post-test; ns: not significant, *$P<0.05$, **$P<0.01$, ***$P<0.001$, and ****$P<0.0001$.

Cell Stem Cell, Volume *30*

Supplemental Information

Organoid modeling of human fetal lung alveolar development reveals mechanisms of cell fate patterning and neonatal respiratory disease

Kyungtae Lim, Alex P.A. Donovan, Walfred Tang, Dawei Sun, Peng He, J. Patrick Pett, Sarah A. Teichmann, John C. Marioni, Kerstin B. Meyer, Andrea H. Brand, and Emma L. Rawlins

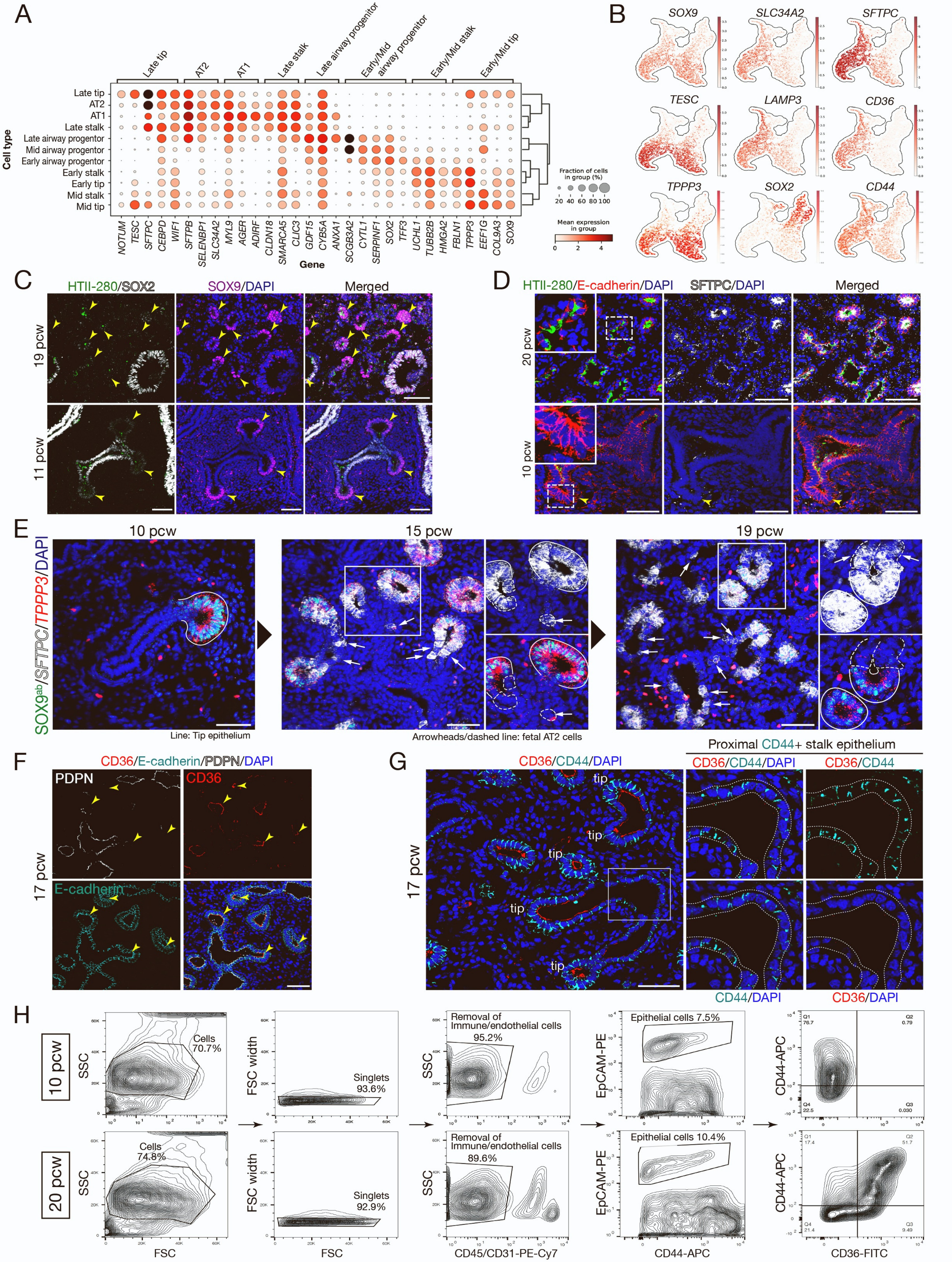
A
Late tip
AT2
AT1
Late stalk
Late airway progenitor
Early/Mid airway progenitor
Early/Mid stalk
Early/Mid tip
Cell type
Late tip
AT2
AT1
Late stalk
Late airway progentor
Mid airway progentor
Early airway progentor
Early stalk
Early tip
Mid stalk
Mid tip
NOTUM TESC SFTPC CEBPD WIF1 SFTPB SELENBP1 SLC34A2 MYL9 AGER ADIRF CLDN18 SMARCA5 CLIC3 GDF15 CYB5A ANXA1 SCGB3A2 CYTL1 SERPINF1 SOX2 TFF3 UCHL1 TUBB2B HMGA2 FBLN1 TPPP3 EEF1G COL9A3 SOX9
Gene
Fraction of cells in group (%)
20 40 60 80 100
Mean expression in group
0 2 4
B
SOX9
SLC34A2
SFTPC
TESC
LAMP3
CD36
TPPP3
SOX2
CD44
C
HTII-280/SOX2
SOX9/DAPI
Merged
19 pcw
11 pcw
D
HTII-280/E-cadherin/DAPI
SFTPC/DAPI
Merged
20 pcw
10 pcw
E
SOX9ab/SFTPC/TPPP3/DAPI
10 pcw
15 pcw
19 pcw
Line: Tip epithelium
Arrowheads/dashed line: fetal AT2 cells
F
CD36/E-cadherin/PDPN/DAPI
PDPN
CD36
E-cadherin
17 pcw
G
CD36/CD44/DAPI
Proximal CD44+ stalk epithelium
CD36/CD44/DAPI
CD36/CD44
tip
17 pcw
CD44/DAPI
CD36/DAPI
H
10 pcw
20 pcw
SSC
FSC
Cells 70.7%
Cells 74.8%
FSC width
Singlets 93.6%
Singlets 92.9%
Removal of Immune/endothelial cells 95.2%
Removal of Immune/endothelial cells 89.6%
CD45/CD31-PE-Cy7
EpCAM-PE
Epithelial cells 7.5%
Epithelial cells 10.4%
CD44-APC
Q1 76.7
Q2 0.79
Q4 22.5
Q3 0.030
Q1 17.4
Q2 51.7
Q4 21.4
Q3 9.49
CD36-FITC

Figure S1. Characterization of the lung tip epithelium at the late stage. Related to Figure 1.

(A and B) Dot plot (A) and UMAP (B) showing representative genes of each cell type in distal human lung tissues from 5 to 22 pcw.

(C and D) Human fetal lung at early-mid and late stages; 11, 19 pcw (C) and 10, 20 pcw (D). Tip epithelium (arrowheads) is marked by E-cadherin, SFTPC, HTII-280, and SOX9. SOX2, airway epithelium (C). DAPI, nuclei. Scale bars, 50 µm.

(E) Time course analysis of human fetal lung tissues at early stage, 10 pcw, and late stages, 15 and 19 pcw, by *in situ* HCR (*SFTPC* and *TPPP3*) and antibody immunostaining ($SOX9^{ab}$). The early-stage tip epithelium at 10 pcw expresses SOX9 and *TPPP3*, and gains the expression of the alveolar lineage marker, *SFTPC*, at late stages, 15 and 19 pcw. The $SFTPC^{+}$ stalk cells (hereafter, fetal AT2 cells) at 15 and 19 pcw lack SOX9 and *TPPP3* expression ($SOX9^{-}TPPP3^{-}SFTPC^{+}$). Lines, tip epithelium. Arrowheads/dashed lines, fetal AT2 cells. DAPI, nuclei. Scale bars, 50 µm.

(F and G) Frozen sections of human fetal lung tissues at 17 pcw. Stained for CD36, E-cadherin and PDPN (F) and CD36, CD44 (G). Arrowheads indicate $CD36^{+}PDPN^{-}$ tips. Inset (G) shows a CD44+, CD36- stalk epithelial region. DAPI, nuclei. Scale bars, 50 µm.

(H) Flow cytometry to isolate the human lung tip epithelial population at 10 (*upper*) and 20 pcw (*lower*). Note that the apparent CD44-,CD36+ population on the bottom-right FACs plot, quadrant 3, cannot be located in the tissue by immunostaining and additional controls show that it is likely to be a FACs-artefact caused by differential sensitivity of the CD36 and CD44 epitopes to digestion by the enzymes used to generate a single cell suspension.

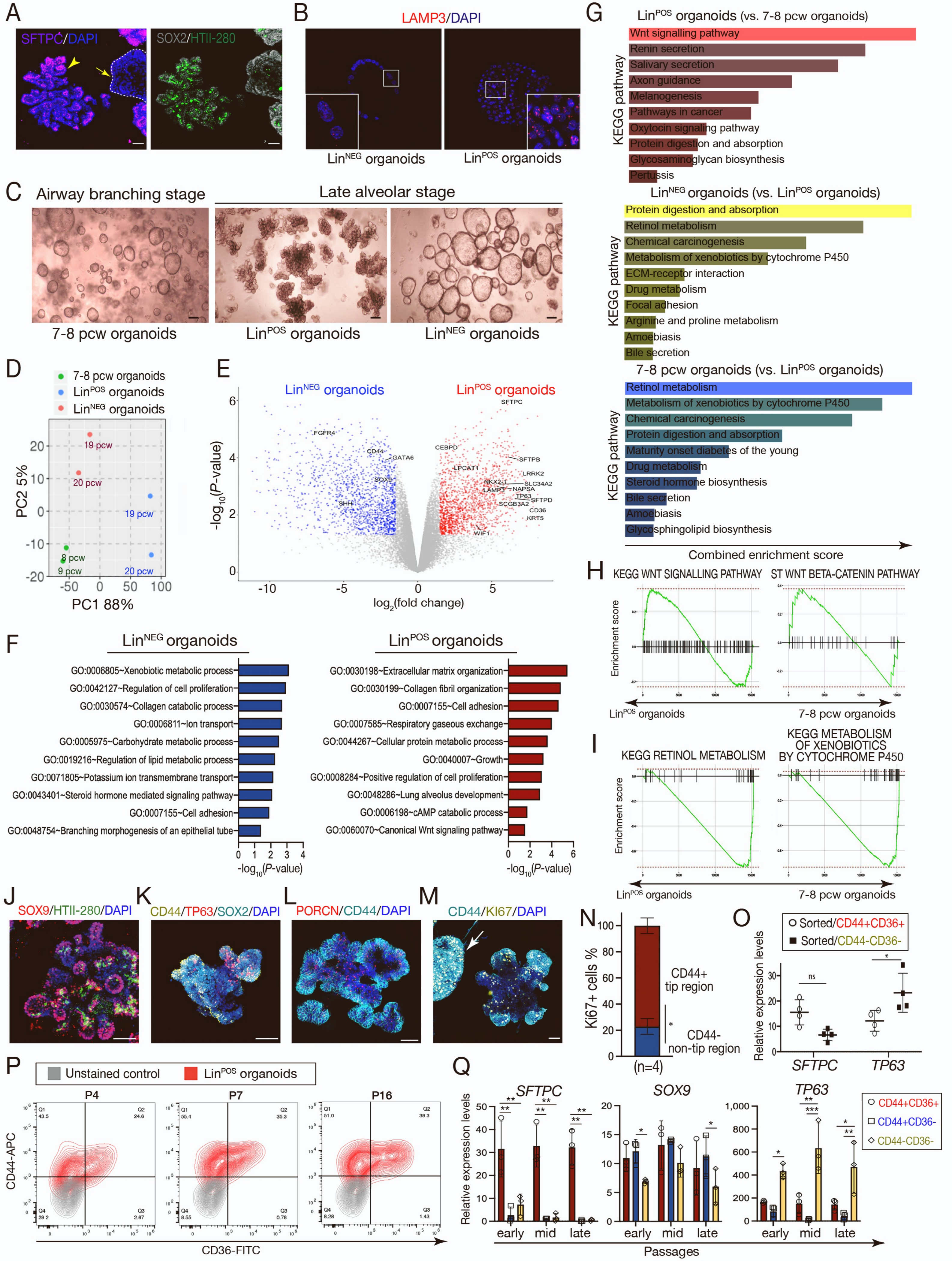

A
SFTPC/DAPI
SOX2/HTII-280
B
LAMP3/DAPI
LinNEG organoids
LinPOS organoids
C
Airway branching stage
Late alveolar stage
7-8 pcw organoids
LinPOS organoids
LinNEG organoids
D
7-8 pcw organoids
LinPOS organoids
LinNEG organoids
PC2 5%
PC1 88%
E
LinNEG organoids
LinPOS organoids
-log10(P-value)
log2(fold change)
F
GO:0006805~Xenobiotic metabolic process
GO:0042127~Regulation of cell proliferation
GO:0030574~Collagen catabolic process
GO:0006811~Ion transport
GO:0005975~Carbohydrate metabolic process
GO:0019216~Regulation of lipid metabolic process
GO:0071805~Potassium ion transmembrane transport
GO:0043401~Steroid hormone mediated signaling pathway
GO:0007155~Cell adhesion
GO:0048754~Branching morphogenesis of an epithelial tube
GO:0030198~Extracellular matrix organization
GO:0030199~Collagen fibril organization
GO:0007155~Cell adhesion
GO:0007585~Respiratory gaseous exchange
GO:0044267~Cellular protein metabolic process
GO:0040007~Growth
GO:0008284~Positive regulation of cell proliferation
GO:0048286~Lung alveolus development
GO:0006198~cAMP catabolic process
GO:0060070~Canonical Wnt signaling pathway
-log10(P-value)
G
LinPOS organoids (vs. 7-8 pcw organoids)
Wnt signalling pathway
Renin secretion
Salivary secretion
Axon guidance
Melanogenesis
Pathways in cancer
Oxytocin signalling pathway
Protein digestion and absorption
Glycosaminoglycan biosynthesis
Pertussis
LinNEG organoids (vs. LinPOS organoids)
Protein digestion and absorption
Retinol metabolism
Chemical carcinogenesis
Metabolism of xenobiotics by cytochrome P450
ECM-receptor interaction
Drug metabolism
Focal adhesion
Arginine and proline metabolism
Amoebiasis
Bile secretion
7-8 pcw organoids (vs. LinPOS organoids)
Retinol metabolism
Metabolism of xenobiotics by cytochrome P450
Chemical carcinogenesis
Protein digestion and absorption
Maturity onset diabetes of the young
Drug metabolism
Steroid hormone biosynthesis
Bile secretion
Amoebiasis
Glycosphingolipid biosynthesis
KEGG pathway
Combined enrichment score
H
KEGG WNT SIGNALLING PATHWAY
ST WNT BETA-CATENIN PATHWAY
Enrichment score
LinPOS organoids
7-8 pcw organoids
I
KEGG RETINOL METABOLISM
KEGG METABOLISM OF XENOBIOTICS BY CYTOCHROME P450
J
SOX9/HTII-280/DAPI
K
CD44/TP63/SOX2/DAPI
L
PORCN/CD44/DAPI
M
CD44/KI67/DAPI
N
Ki67+ cells %
CD44+ tip region
CD44- non-tip region
(n=4)
O
Sorted/CD44+CD36+
Sorted/CD44-CD36-
Relative expression levels
SFTPC
TP63
P
Unstained control
LinPOS organoids
P4
P7
P16
CD44-APC
CD36-FITC
Q
SFTPC
SOX9
TP63
CD44+CD36+
CD44+CD36-
CD44-CD36-
Relative expression levels
early
mid
late
Passages

Figure S2. Characterization of the late-stage lung tip organoids. Related to Figure 2.

(A and B) Immunofluorescence analysis of the Lin^{POS} (arrowhead) and Lin^{NEG} organoids (arrow) at passage 1 cultured in the self-renewing medium, showing the alveolar lineage markers, SFTPC, HTII-280 (A) and LAMP3 (B, *lower* panel) were expressed in the Lin^{POS} organoids, but not in the Lin^{NEG} organoids. DAPI, nuclei. Scale bar, 50 μm.

(C) Morphology of 7-8 pcw organoids derived from airway branching stage, and the Lin^{POS} and Lin^{NEG} organoids from late alveolar stage. The Lin^{POS} and Lin^{NEG} organoids grown from $EPCAM^+$ tip epithelial cells were manually separated and cultured in the self-renewing culture condition.

(D) Principal component analysis of bulk-RNA seq data using 7-8 pcw organoids, Lin^{POS} and Lin^{NEG} organoids.

(E) Volcano plot showing differentially expressed genes between Lin^{POS} organoids (*red*) versus Lin^{NEG} organoids (*dark blue*); $\log_2 FC > 4$.

(F) Gene ontology (GO) enrichment analysis performed for biological process (BP)- associated GO terms on the differentially expressed genes between the Lin^{POS} and Lin^{NEG} organoids; $\log_2 FC > 4$.

(G) KEGG pathway analysis using Enrichr. Length of coloured bars indicates combined enrichment score by adjusted p-value < 0.05.

(H and I) Gene set enrichment (GSEA) analysis of the differentially expressed genes of the Lin^{POS} organoids (H) and 7-8 pcw organoids (I).

(J-M) Immunofluorescence images of the Lin^{NEG} and Lin^{POS} organoids originating from sorted $CD44^+CD36^+$ tip epithelium from 20 pcw lung. Antibodies against SOX9, HTII-280 (J), CD44, TP63, SOX2 (K), CD44, PORCN (L) and CD44, KI67 (M). Arrow (M) indicates a Lin^{NEG} organoid. DAPI indicates nuclei. Scale bar, 50 μm.

(N) The percentage of $KI67^+$ cells in the $CD44^+$ tip and $CD44^-$ non-tip regions are represented as mean ± SD of biological 4 replicates.

(O) Expression of lineage markers was investigated by qRT-PCR in the Lin^{POS} organoids and airway-like spheres at passage 1 derived from the $CD44^+CD36^+$ or $CD44^-CD36^-$ passage zero subpopulations respectively. Data was normalized to $EPCAM^+$ cells freshly sorted from 20 pcw tip tissues and represented as mean ± SD of 4 biological replicates. Significance was evaluated by 2-way ANOVA with Bonferroni multiple comparison post-test; ns: not significant, $*P<0.05$.

(P and Q). FACS analysis (P) of the cellular composition of Lin^{POS} organoid lines at multiple passages: early passage (2-5); mid passage (6-12); late passages (14-20) by CD36 (FITC) and CD44 (APC) and gene expression profiling (Q) of each fraction, $CD44^+CD36^+$, $CD44^+CD36^-$, and $CD44^-CD36^-$, by qRT-PCR. Red, stained. Gray, unstained control. Data normalized to fresh EPCAM+ cells from 20 pcw distal tissues; mean ± SD of biological 3 replicates. Significance was evaluated by 2-way ANOVA with Tukey multiple comparison post-test; * $P<0.05$, ** $P<0.01$, *** $P<0.001$, and ****$P<0.0001$.

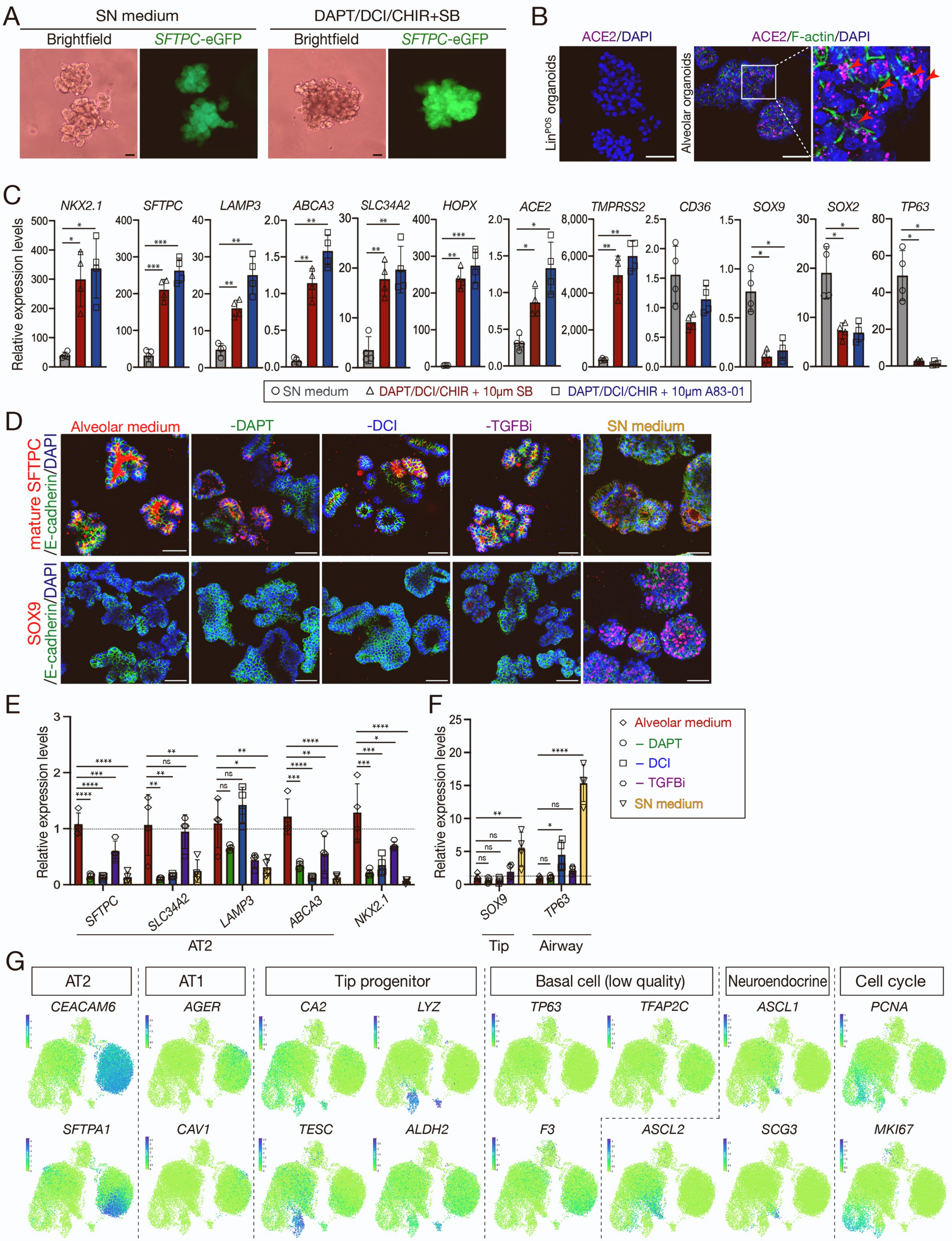

A
SN medium
DAPT/DCI/CHIR+SB
Brightfield
SFTPC-eGFP
B
ACE2/DAPI
ACE2/F-actin/DAPI
Lin^POS organoids
Alveolar organoids
C
NKX2.1
SFTPC
LAMP3
ABCA3
SLC34A2
HOPX
ACE2
TMPRSS2
CD36
SOX9
SOX2
TP63
Relative expression levels
○ SN medium
△ DAPT/DCI/CHIR + 10μm SB
□ DAPT/DCI/CHIR + 10μm A83-01
D
Alveolar medium
-DAPT
-DCI
-TGFBi
SN medium
mature SFTPC/E-cadherin/DAPI
SOX9/E-cadherin/DAPI
E
Relative expression levels
SFTPC
SLC34A2
LAMP3
ABCA3
NKX2.1
AT2
F
Relative expression levels
SOX9
TP63
Tip
Airway
Alveolar medium
– DAPT
– DCI
– TGFBi
SN medium
G
AT2
AT1
Tip progenitor
Basal cell (low quality)
Neuroendocrine
Cell cycle
CEACAM6
AGER
CA2
LYZ
TP63
TFAP2C
ASCL1
PCNA
SFTPA1
CAV1
TESC
ALDH2
F3
ASCL2
SCG3
MKI67

Figure S3. Alveolar differentiation of the cultured late stage tip cells. Related to Figure 3.

(A) Morphology and fluorescent images of the Lin^{POS} organoids cultured in control SN medium, or in DAPT/DCI/CHIR with SB, for 1 week.

(B) Immunofluorescent analysis of ACE2 in the Lin^{POS} organoids and alveolar organoids. Phalloidin (F-actin) marks apical membrane of epithelial cells in the organoids. Scale bar, 50 µm.

(C) qRT-PCR of Lin^{POS} organoids cultured in the SN medium or in DAPT/DCI/CHIR with SB, or A83-01, for 1 week. Data were normalized to $EPCAM^{+}$ cells freshly isolated from 20 pcw tip tissues; mean ± SD of four biological replicates. Significance was evaluated by 1-way ANOVA with Tukey multiple comparison post-test; **P*<0.05, ***P*<0.01, ****P*<0.001.

(D-F) Fluorescence (D) and qRT-PCR (E, F) analysis of the alveolar organoids upon withdrawal of each component, DAPT, DCI, or TGFBi (SB431542), from the alveolar differentiation medium for 1 week. Expression level in SN medium is shown as the negative control. Data normalized to the alveolar medium condition; mean ± SD, *n* = 3. Significance evaluated by 1-way ANOVA with Tukey multiple comparison post-test; ns: not significant, **P*<0.05, ***P*<0.01, ****P*<0.001, *****P*<0.0001. DAPI, nuclei. Scale bar, 50 µm.

(G) UMAP plots showing transcript expression of marker genes specific to AT2, AT1, tip progenitor, basal cell, neuroendocrine lineages and cell cycle.

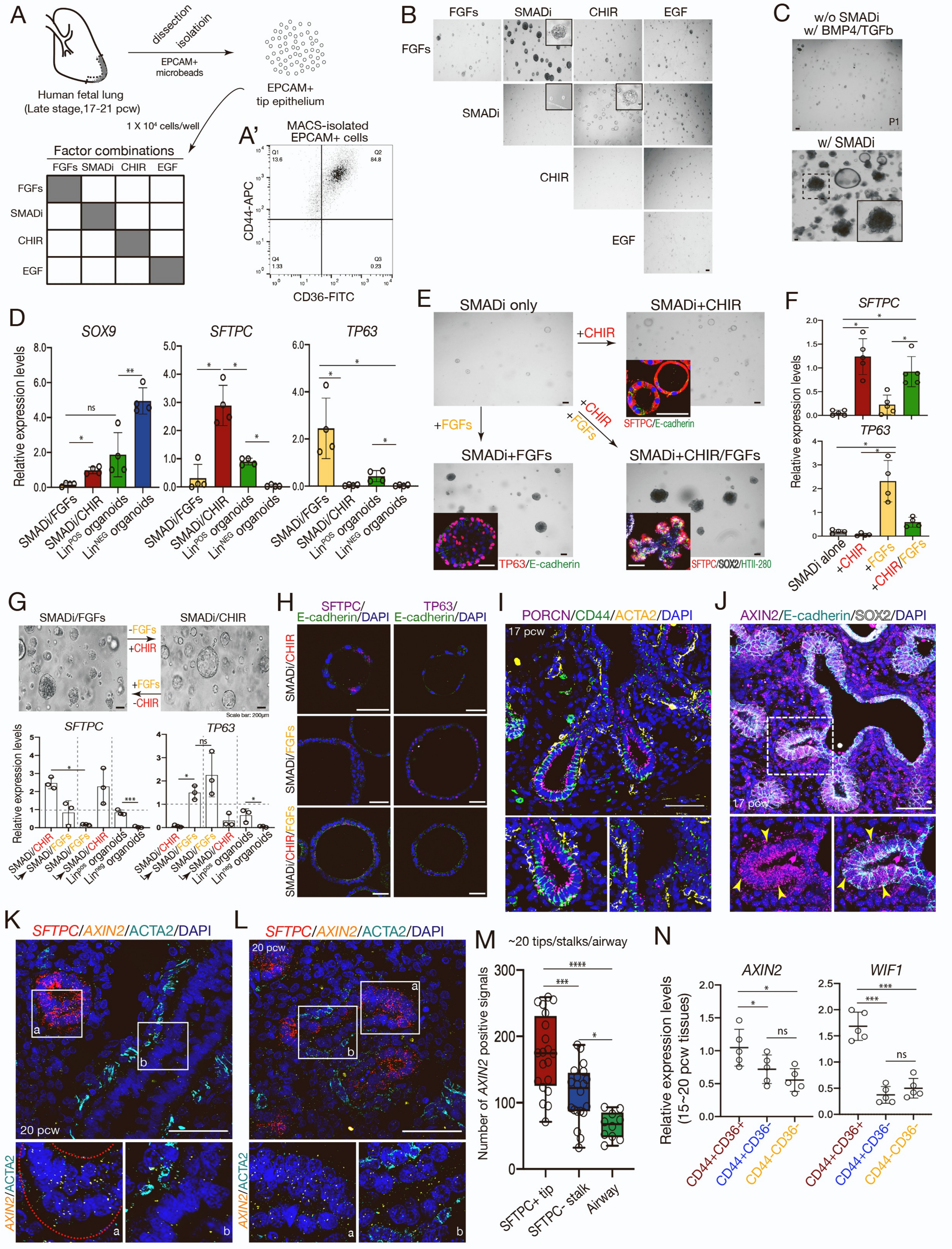

A
Human fetal lung (Late stage,17-21 pcw)
dissection
isolatioin
EPCAM+ microbeads
EPCAM+ tip epithelium
1 X 10⁴ cells/well
Factor combinations
FGFs SMADi CHIR EGF
A'
MACS-isolated EPCAM+ cells
CD44-APC
CD36-FITC
B
FGFs SMADi CHIR EGF
C
w/o SMADi w/ BMP4/TGFb
P1
w/ SMADi
D
SOX9
SFTPC
TP63
Relative expression levels
SMADi/FGFs
SMADi/CHIR
Lin^POS organoids
Lin^NEG organoids
E
SMADi only
SMADi+CHIR
+CHIR
+FGFs
SMADi+FGFs
SMADi+CHIR/FGFs
SFTPC/E-cadherin
TP63/E-cadherin
SFTPC/SOX2/HTII-280
F
SFTPC
TP63
Relative expression levels
SMADi alone
+CHIR
+FGFs
+CHIR/FGFs
G
SMADi/FGFs
SMADi/CHIR
-FGFs +CHIR
+FGFs -CHIR
Scale bar: 200μm
SFTPC
TP63
Relative expression levels
H
SFTPC/ E-cadherin/DAPI
TP63/ E-cadherin/DAPI
SMADi/CHIR
SMADi/FGFs
SMADi/CHIR/FGFs
I
PORCN/CD44/ACTA2/DAPI
17 pcw
J
AXIN2/E-cadherin/SOX2/DAPI
17 pcw
K
SFTPC/AXIN2/ACTA2/DAPI
20 pcw
AXIN2/ACTA2
L
SFTPC/AXIN2/ACTA2/DAPI
20 pcw
AXIN2/ACTA2
M
~20 tips/stalks/airway
Number of AXIN2 positive signals
SFTPC+ tip
SFTPC- stalk
Airway
N
AXIN2
WIF1
Relative expression levels (15~20 pcw tissues)
CD44+CD36+
CD44+CD36-
CD44-CD36-

Figure S4. High plasticity of the late-stage tip epithelial cells in response to the signalling cues. Related to Figure 4.

(A) Diagram showing *in vitro* culture of the freshly isolated tip epithelial cells for 2 weeks in single or pairwise combinations of signalling cues: FGFs (FGF7, FGF10), SMADi (Noggin, SB431542), CHIR (CHIR99021) and EGF. (A') The isolated EPCAM$^+$ tip epithelial cells from the distal lung tissue at 20 pcw were analysed by CD44 and CD36 expression using FACS.

(B) Morphology of the tip organoids cultured in different culture conditions for 2 weeks. Representative image from 1 biological replicate is shown; n=4 biological replicates in total. Scale bar, 200 µm.

(C) Tip epithelial cells cultured with, or without, SMAD inhibition for 3 weeks. The self-renewal medium condition (w/ SMADi; *lower* panel) was used for positive control. Scale bar, 200 µm.

(D) Relative mRNA levels of *SOX9*, *SFTPC* and *TP63* measured by qRT-PCR. Normalized to a Lin^{POS} organoid line; mean ± SD of 4 independent biological replicates. Significance was evaluated by 1-way ANOVA with Tukey multiple comparison post-test; ns: not significant, *P<0.05 and **P<0.01.

(E and F) After 2 weeks growing in SMADi conditions, cells were sequentially transferred to culture medium containing CHIR, or FGFs, or CHIR/FGFs. After a further 2 weeks of exposure to the different culture conditions, the epithelial organoids were stained with lineage makers including SFTPC or TP63 (E) and relative mRNA levels of *SFTPC* and *TP63* were measured by qRT-PCR (F). Data were normalized to the Lin^{POS} organoids; mean ± SD of at least 4 biological replicates. Significance was evaluated by 1-way ANOVA with Tukey multiple comparison post-test; ns: not significant, *P<0.05, **P<0.01 and ***P<0.001. Scale bar, 200 µm.

(G) Dynamic cell fate plasticity of the tip epithelial cells depending on Wnt and FGF signals. The morphology of the epithelial organoids was changed when the culture conditions were switched from SMADi/FGFs to SMADi/CHIR, or vice versa (upper panels). The gene expression profile (lower panels) was analysed by qRT-PCR following 2 weeks exposure to the 2nd medium. Data were normalized to freshly isolated Lin^{POS} organoids; mean ± SD of 3 biological replicates. Significance was evaluated by 1-way ANOVA with Tukey multiple comparison post-test; ns: not significant, *P<0.05, **P<0.01, and ***P<0.001.

(H) Immunofluorescence analysis of 8 pcw organoids in the different conditions at passage 0. Antibodies against SFTPC, TP63, E-cadherin and SOX2 were used. Scale bar, 50 µm.

(I-M) Frozen sections of human fetal lung tissues at 17 pcw (I,J) and 20 pcw (K,L) were immunostained for PORCN, CD44, and ACTA2 (I), AXIN2, E-cadherin and SOX2 (J), or ACTA2 followed by *in situ* HCR for *SFTPC* and *AXIN2* (K, tip (a) and airway (b); L, tip (a) and stalk (b)). Arrowheads (J) indicate AXIN2$^+$ tip epithelial cells. Red dashed line in the inset (K) indicates *SFTPC*$^+$ tip epithelial cells. *AXIN2* signals were counted from 20 areas of tips and stalks and 10 areas of airway across 3 independent lung tissues at 18-20 pcw (M). Significance was evaluated by 1-way ANOVA

with Tukey multiple comparison post-test; ns: not significant, *$P<0.05$, **$P<0.01$, ***$P<0.001$ and ****$P<0.0001$. Scale bar, 50 μm.

(N) Gene expression profile of the freshly isolated lung epithelial cells from the late stage human lung tissues sorted by $CD44^{+}CD36^{+}$, $CD44^{+}CD36^{-}$ and $CD44^{-}CD36^{-}$. Data normalized to freshly isolated $EPCAM^{+}$ cells from 20 pcw tip tissues; mean ± SD of 5 biological replicates aged from 15~20 pcw. Significance was evaluated by 1-way ANOVA with Tukey multiple comparison post-test; ns: not significant, *$P<0.05$, **$P<0.01$ and ***$P<0.001$.

DAPI indicates nuclei.

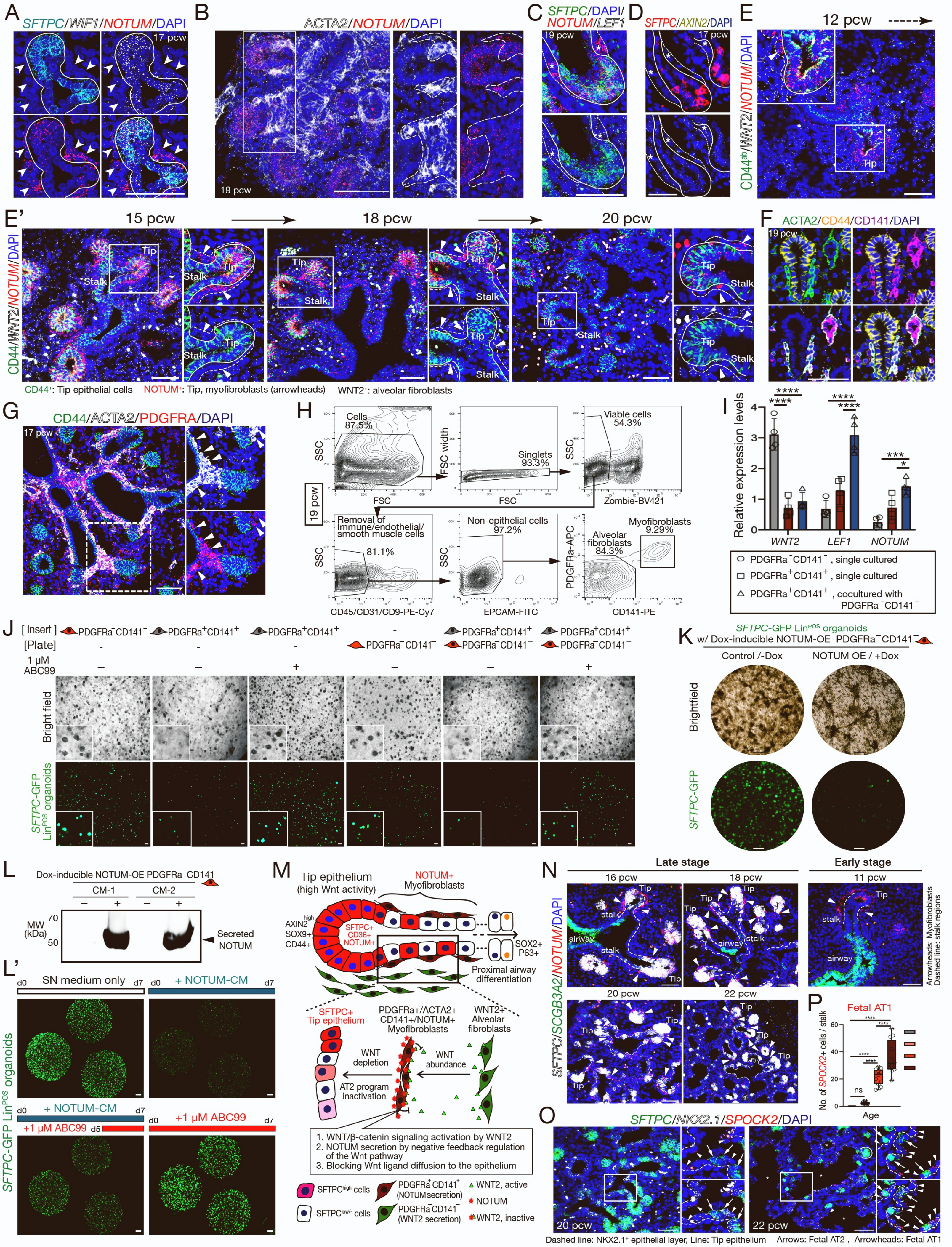

A
SFTPC/WIF1/NOTUM/DAPI
17 pcw
B
ACTA2/NOTUM/DAPI
19 pcw
C
SFTPC/DAPI/NOTUM/LEF1
19 pcw
D
SFTPC/AXIN2/DAPI
17 pcw
E
12 pcw
CD44ab/WNT2/NOTUM/DAPI
Tip
E'
15 pcw
18 pcw
20 pcw
CD44/WNT2/NOTUM/DAPI
Tip
Stalk
CD44+: Tip epithelial cells
NOTUM+: Tip, myofibroblasts (arrowheads)
WNT2+: alveolar fibroblasts
F
ACTA2/CD44/CD141/DAPI
19 pcw
G
CD44/ACTA2/PDGFRA/DAPI
17 pcw
H
19 pcw
Cells 87.5%
Singlets 93.3%
Viable cells 54.3%
SSC
FSC
FSC width
Zombie-BV421
Removal of Immune/endothelial/smooth muscle cells
81.1%
Non-epithelial cells 97.2%
Myofibroblasts 9.29%
Alveolar fibroblasts 84.3%
CD45/CD31/CD9-PE-Cy7
EPCAM-FITC
CD141-PE
PDGFRa-APC
I
Relative expression levels
WNT2
LEF1
NOTUM
PDGFRa−CD141−, single cultured
PDGFRa+CD141+, single cultured
PDGFRa+CD141+, cocultured with PDGFRa−CD141−
J
[Insert]
[Plate]
1 μM ABC99
PDGFRa−CD141−
PDGFRa+CD141+
Bright field
SFTPC-GFP LinPOS organoids
K
SFTPC-GFP LinPOS organoids
w/ Dox-inducible NOTUM-OE PDGFRa−CD141−
Control /-Dox
NOTUM OE / +Dox
Brightfield
SFTPC-GFP
L
Dox-inducible NOTUM-OE PDGFRa−CD141−
CM-1
CM-2
MW (kDa)
70
50
Secreted NOTUM
L'
SN medium only
+ NOTUM-CM
+1 μM ABC99
d0
d5
d7
SFTPC-GFP LinPOS organoids
M
Tip epithelium (high Wnt activity)
NOTUM+ Myofibroblasts
AXIN2high SOX9+ CD44+
SFTPC+ CD36+ NOTUM+
SOX2+ P63+
Proximal airway differentiation
SFTPC+ Tip epithelium
PDGFRa+/ACTA2+ CD141+/NOTUM+ Myofibroblasts
WNT2+ Alveolar fibroblasts
WNT depletion
WNT abundance
AT2 program inactivation
1. WNT/β-catenin signaling activation by WNT2
2. NOTUM secretion by negative feedback regulation of the Wnt pathway
3. Blocking Wnt ligand diffusion to the epithelium
SFTPChigh cells
SFTPClow/- cells
PDGFRa+CD141+ (NOTUM secretion)
PDGFRa−CD141− (WNT2 secretion)
WNT2, active
NOTUM
WNT2, inactive
N
Late stage
Early stage
16 pcw
18 pcw
11 pcw
20 pcw
22 pcw
SFTPC/SCGB3A2/NOTUM/DAPI
Tip
stalk
airway
Arrowheads: Myofibroblasts
Dashed line: stalk regions
P
Fetal AT1
No. of SPOCK2+ cells / stalk
Age
ns
O
SFTPC/NKX2.1/SPOCK2/DAPI
20 pcw
22 pcw
Dashed line: NKX2.1+ epithelial layer, Line: Tip epithelium
Arrows: Fetal AT2, Arrowheads: Fetal AT1

Figure S5. Wnt-responsive NOTUM$^+$ myofibroblasts in the distal human fetal lung pattern differentiating alveolar epithelium. Related to Figure 4.

(A-D) Fluorescence imaging of human fetal lung sections at 17 (A, D) and 19 pcw (B, C), *in situ* HCR and immunostaining. (A) *SFTPC*, *WIF1*, *NOTUM*. (B) ACTA2, *NOTUM.* (C) *SFTPC*, *NOTUM, LEF1*. (D) *SFTPC*, *AXIN2*. Arrowheads and asterisks indicate ACTA2$^+$ *NOTUM*$^+$ myofibroblasts. Lines and dashed lines indicate the boundaries of epithelial cells and myofibroblasts, respectively.

(E) Time course analysis of distal human lung tissues at 12, 15, 18, and 20 pcw, using *in situ* HCR and immunostaining. *WNT2*, alveolar fibroblasts, white; *NOTUM*, myofibroblasts, red; CD44, tip epithelium, green. Arrowheads indicate myofibroblast population at the adjacent tip regions. Lines and dashed lines indicate the boundaries of epithelial cells and myofibroblasts, respectively.

(F, G) Identification of a combination of surface antigens, CD141 and PDGFRA, targeting the myofibroblasts, by immunostaining. (F) ACTA2, CD44, CD141. (G) CD44, ACTA2, PDGFRA.

(H) Flow cytometry to isolate alveolar fibroblasts and myofibroblasts from the late-stage lung at 19 pcw.

(I) Relative mRNA levels from myofibroblasts and alveolar fibroblasts cultured alone, or cocultured in transwells. Data were normalized to the whole freshly isolated lung fibroblast population; mean ± SD of biological 4 replicates. Significance was evaluated by unpaired student *t*-test; **P*<0.05, ***P*<0.01, ****P*<0.001.

(J) *In vitro* coculture assay of *SFTPC*-GFP$^+$ Lin^{POS} organoids with freshly isolated PDGFRA$^-$CD141$^-$ alveolar fibroblasts and/or PDGFRA$^+$CD141$^+$ myofibroblasts. 1µM ABC99 was used to inhibit NOTUM activity. Scale bar, 100 µm.

(K) Coculture of the *SFTPC*-GFP$^+$ Lin^{POS} organoids with the alveolar fibroblasts overexpressing doxycycline (Dox)-inducible NOTUM (NOTUM-OE), in DMEM/FBS 2% medium for 1 week. Scale bar, 100 µm.

(L, L') Conditioned medium (CM) was collected every 2 days for 1 week from the cultured PDGFRA$^-$ CD141$^-$ NOTUM-OE alveolar fibroblasts in the presence or absence of Dox and concentrated using 30K MWCO Concentrator (Thermo Fisher; 88529). (L) Western blot detection of the secreted NOTUM in the CM. DMEM+FBS 2% medium were used for the CM preparation; N = 2. (L') The *SFTPC*-GFP Lin^{POS} organoids were treated with/without the CM in the presence or absence of 1 µM ABC99, in the self-renewal (SN) medium, for 1 week. Scale bar, 100 µm.

(M) Summary diagram showing the spatial regulation of Wnt signalling mediated by ACTA2$^+$PDGFRa$^+$CD141$^+$ myofibroblasts in the distal regions of human lung tissues during the late-stage.

(N) Time course analysis of *NOTUM*+ myofibroblasts, *SCGB3A2*$^+$ terminal airway cells, and *SFTPC*$^+$ tip and stalk cells from early (11 pcw) to late stages (16, 18, 20, and 22 pcw), by *in situ* HCR. At the early stage (at 11 pcw), the tip epithelium that alveolar differentiation competence is absent, directly

differentiates to *SCGB3A2*$^+$ distal airway after a short range of intermediate stalks. At the later stages of development, the *SCGB3A2*$^+$ airway cells are rarely observed in the distal lung regions near the distal tip/stalk; e.g. 20 pcw afterward. Instead, from 16 to 22 pcw, the alveolar fated-tip epithelium facing *NOTUM*$^+$ myofibroblasts began to produce the *SFTPC*+ fetal AT2 cells along the tip-stalk axis until reaching at the *SCGB3A2*$^+$ terminal bronchiole regions. *NOTUM*, red; *SCGB3A2*, green; white, *SFTPC*. DAPI, nuclei. Arrowheads and dashed line indicate the myofibroblasts and stalk epithelial cells, respectively.

(O and P) Time-course visualization (O) and quantitation (P) of *SFTPC*$^-$/*SPOCK2*$^+$ stalk cells (hereafter, fetal AT1 cells) in the fetal lung tissues at 20 pcw afterward, analysed by *in situ* HCR. The fetal AT1 cells are located at the stalk regions, next to the fetal AT2 cells. *SPOCK2*, red, AT1 cell marker; *SFTPC*, green, AT2 cell marker; white, *NKX2.1*, lung epithelial cell marker. DAPI, nuclei. Arrowheads, fetal AT1 cells. Arrows, fetal AT2 cells. Line, tip epithelium. Dashed line, NKX2.1+ epithelial layer.

DAPI indicates nuclei. Scale bar, 50 µm.

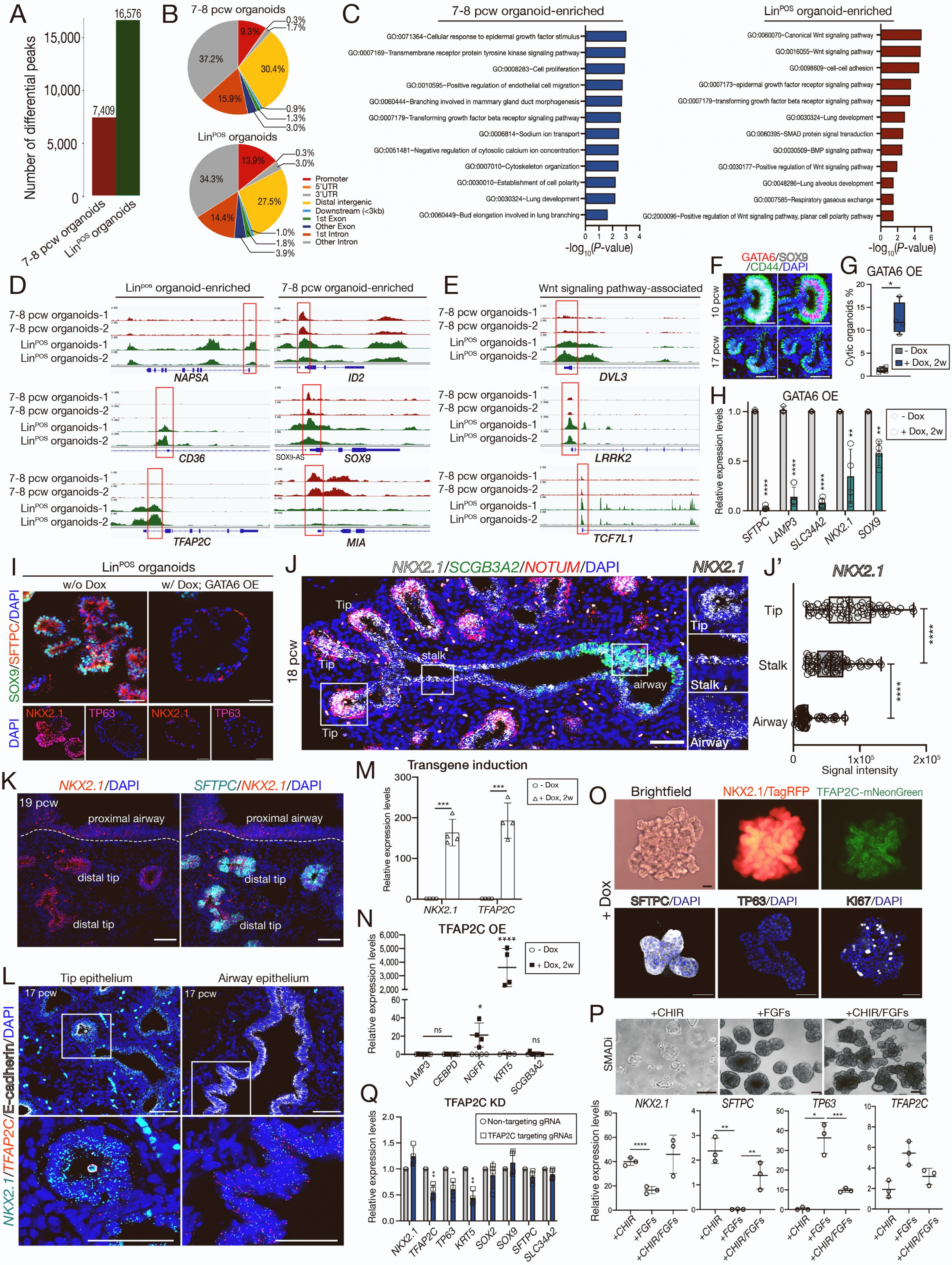
A
Number of differential peaks
16,576
7,409
15,000
10,000
5,000
0
7-8 pcw organoids
Lin^POS organoids
B
7-8 pcw organoids
9.3%
0.3%
1.7%
37.2%
30.4%
15.9%
0.9%
1.3%
3.0%
Lin^POS organoids
13.9%
0.3%
3.0%
34.3%
27.5%
14.4%
1.0%
1.8%
3.9%
Promoter
5'UTR
3'UTR
Distal intergenic
Downstream (<3kb)
1st Exon
Other Exon
1st Intron
Other Intron
C
7-8 pcw organoid-enriched
GO:0071364~Cellular response to epidermal growth factor stimulus
GO:0007169~Transmembrane receptor protein tyrosine kinase signaling pathway
GO:0008283~Cell proliferation
GO:0010595~Positive regulation of endothelial cell migration
GO:0060444~Branching involved in mammary gland duct morphogenesis
GO:0007179~Transforming growth factor beta receptor signaling pathway
GO:0006814~Sodium ion transport
GO:0051481~Negative regulation of cytosolic calcium ion concentration
GO:0007010~Cytoskeleton organization
GO:0030010~Establishment of cell polarity
GO:0030324~Lung development
GO:0060449~Bud elongation involved in lung branching
-log10(P-value)
Lin^POS organoid-enriched
GO:0060070~Canonical Wnt signaling pathway
GO:0016055~Wnt signaling pathway
GO:0098609~cell-cell adhesion
GO:0007173~epidermal growth factor receptor signaling pathway
GO:0007179~transforming growth factor beta receptor signaling pathway
GO:0030324~Lung development
GO:0060395~SMAD protein signal transduction
GO:0030509~BMP signaling pathway
GO:0030177~Positive regulation of Wnt signaling pathway
GO:0048286~Lung alveolus development
GO:0007585~Respiratory gaseous exchange
GO:2000096~Positive regulation of Wnt signaling pathway, planar cell polarity pathway
-log10(P-value)
D
Lin^pos organoid-enriched
7-8 pcw organoid-enriched
7-8 pcw organoids-1
7-8 pcw organoids-2
Lin^POS organoids-1
Lin^POS organoids-2
NAPSA
ID2
CD36
SOX9-AS
SOX9
TFAP2C
MIA
E
Wnt signaling pathway-associated
DVL3
LRRK2
TCF7L1
F
GATA6/SOX9/CD44/DAPI
10 pcw
17 pcw
G
GATA6 OE
Cytic organoids %
- Dox
+ Dox, 2w
H
GATA6 OE
Relative expression levels
- Dox
+ Dox, 2w
SFTPC
LAMP3
SLC34A2
NKX2.1
SOX9
I
Lin^POS organoids
w/o Dox
w/ Dox; GATA6 OE
SOX9/SFTPC/DAPI
NKX2.1
TP63
DAPI
J
NKX2.1/SCGB3A2/NOTUM/DAPI
18 pcw
Tip
stalk
airway
NKX2.1
Stalk
Airway
J'
NKX2.1
Tip
Stalk
Airway
Signal intensity
0
1x10^5
2x10^5
K
NKX2.1/DAPI
SFTPC/NKX2.1/DAPI
19 pcw
proximal airway
distal tip
L
Tip epithelium
Airway epithelium
17 pcw
NKX2.1/TFAP2C/E-cadherin/DAPI
M
Transgene induction
Relative expression levels
- Dox
+ Dox, 2w
NKX2.1
TFAP2C
N
TFAP2C OE
Relative expression levels
- Dox
+ Dox, 2w
LAMP3
CEBPD
NGFR
KRT5
SCGB3A2
ns
O
Brightfield
NKX2.1/TagRFP
TFAP2C-mNeonGreen
+ Dox
SFTPC/DAPI
TP63/DAPI
KI67/DAPI
P
+CHIR
+FGFs
+CHIR/FGFs
SMADi
Relative expression levels
NKX2.1
SFTPC
TP63
TFAP2C
Q
TFAP2C KD
Relative expression levels
Non-targeting gRNA
TFAP2C targeting gRNAs
NKX2.1
TFAP2C
TP63
KRT5
SOX2
SOX9
SFTPC
SLC34A2

Figure S6. NKX2.1 drives the onset of the alveolar program whilst supressing the airway program. Related to Figure 5.

(A) Analysis of chromatin accessibility in the 7-8 pcw and Lin^{POS} organoids by bulk-ATAC seq. Approximately 90,000 peaks were found in total, and 7,409 and 16,576 of differentially enriched peaks were identified in between 7-8 pcw organoids and Lin^{POS} organoids, respectively (Table S2; fold change > 2 and FDR< 0.05).

(B) Pie charts representing the genomic distribution of global accessible chromatin regions in the 7-8 pcw organoids and Lin^{POS} organoids.

(C) Biological Process-associated GO term analysis using the differential accessible chromatin regions highly enriched in the 7-8 pcw organoids and Lin^{POS} organoids.

(D and E) IGV image shots of representative ATAC seq tracks at loci showing differentially accessible chromatin regions between the 7-8 pcw organoids and Lin^{POS} organoids. Red box indicates the promoter regions.

(F) Fluorescence imaging of tip epithelium in 10 and 17 pcw lung tissues, by immunostaining. Red, GATA6; white, SOX9; green, CD44.

(G) Proportions of cystic organoids morphologically converted from the folded Lin^{POS} organoids were counted after 2 weeks of GATA6 overexpression in the self-renewal medium. Significance was evaluated by student t-test; *P<0.05.

(H, I) Gene expression (H) and immunofluorescence (I) analysis of the Lin^{POS} organoids overexpressed with GATA6. The qRT-PCR data (H) was normalized to the Lin^{POS} organoids treated without Dox; mean ± SD of biological 4 replicates. Data was normalized to the Lin^{POS} organoids treated with DMSO only; mean ± SD of biological 3 replicates. Significance was evaluated by 1-way ANOVA (H); *P<0.05, **P<0.01, ***P<0.001, ****P<0.0001.

(J) *In situ* HCR analysis of human fetal lung tissues at 18 pcw. *NKX2.1*, white; *NOTUM*, red; *SCGB3A2*, green. Dashed line indicates the boundary of the stalk epithelial tube. (J') Intensity of NKX2.1 signals at tip, stalk, and airway regions were measured using ImageJ. Mean ± SD of 4 biological replicates of late stage lungs at different ages, 15 to 18 pcw. Significance was evaluated by one-way ANOVA; *P<0.05, **P<0.01, ***P<0.001, ****P<0.0001.

(K and L) *In situ* HCR images for detecting transcripts, *SFTPC* or *TFAP2C* with *NKX2.1,* followed by immunostaining (L. E-cadherin), in 17 (L) and 19 pcw (K) fetal lung tissues.

(M) Transgene induction following doxycycline treatment for 2 weeks measured by qRT-PCR. Data was normalized to the untreated group; mean ± SD of four biological replicates. Significance was evaluated by 1-way ANOVA with Tukey multiple comparison post-test; ns: not significant, ****P<0.0001.

(N) qRT-PCR analysis of 7-9 pcw organoids overexpressing TFAP2C for 2 weeks. Data was normalized to $EPCAM^+$ positive cells freshly isolated from 20 pcw tip tissues; mean ± SD of

biological 4 replicates. Significance was evaluated by 1-way ANOVA with Tukey multiple comparison post-test; ns: not significant, **P*<0.05, ***P*<0.01, ****P*<0.001 and *****P*<0.0001.

(O) Morphology and fluorescent images of the 7-9 pcw organoids overexpressing both NKX2.1 and TFAP2C for 2 weeks.

(P) qRT-PCR of endogenous *NKX2.1*, *SFTPC*, and *TP63* in the Lin^{POS} organoids cultured in medium containing CHIR, FGF7 or CHIR/FGF7. Data was normalized to EPCAM^{+} cells freshly isolated from 20 pcw tip tissues; mean ± SD of three biological replicates. Significance was evaluated by 1-way ANOVA with Tukey multiple comparison post-test; ns: not significant, **P*<0.05, ***P*<0.01, ****P*<0.001, *****P*<0.0001.

(Q) Knock-down (KD) of endogenous *TFAP2C* in the Lin^{POS} organoids by CRISPR-dCas9-KRAB system. Data was normalized to non-targeting gRNAs; mean ± SD of 3 biological replicates. Significance was evaluated by 1-way ANOVA with Tukey multiple comparison post-test; **P*<0.05, ***P*<0.01, ****P*<0.001, *****P*<0.0001.

DAPI indicates nuclei. Scale bar, 50 µm.

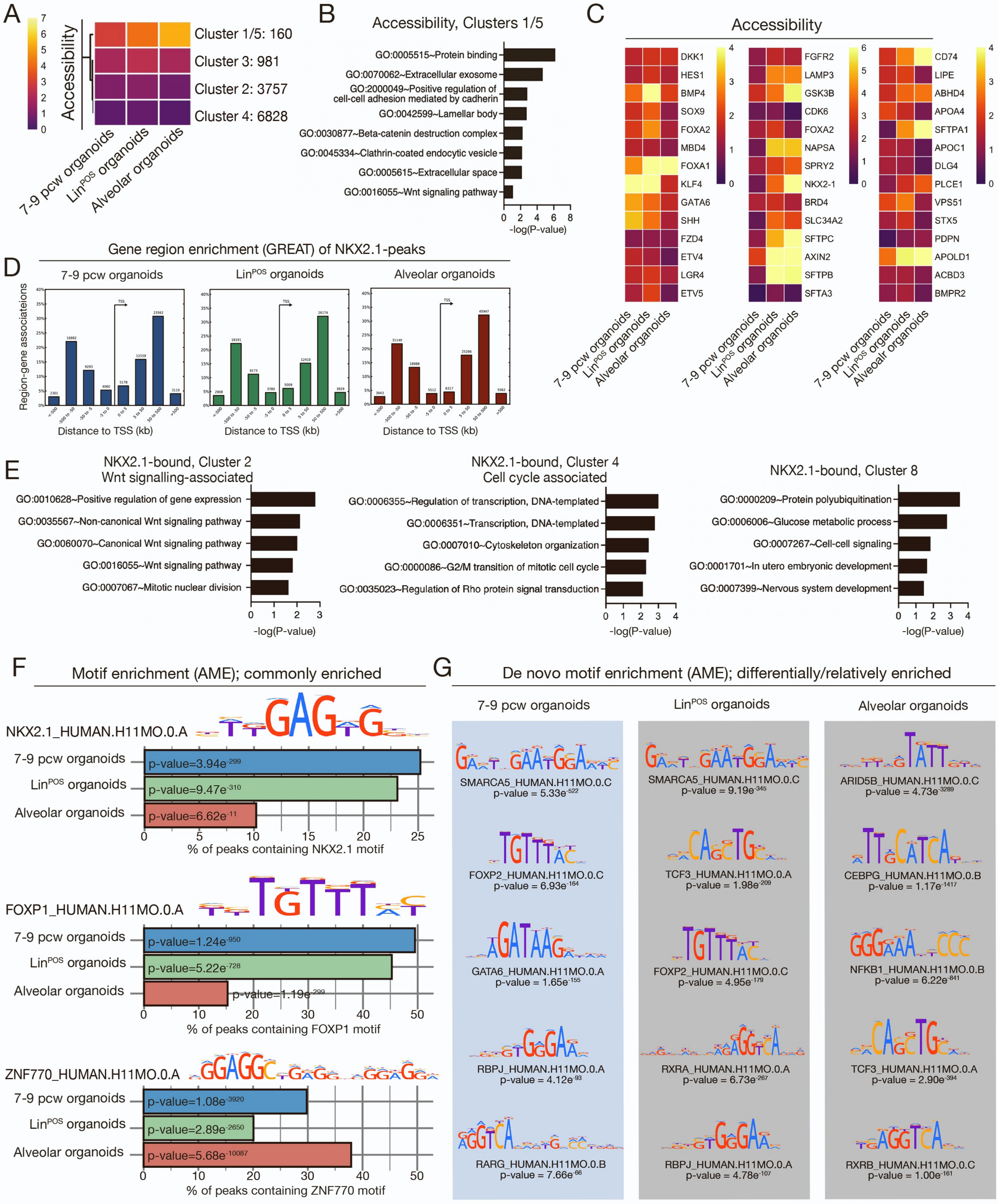

A
Accessibility
Cluster 1/5: 160
Cluster 3: 981
Cluster 2: 3757
Cluster 4: 6828
7-9 pcw organoids
LinPOS organoids
Alveolar organoids
B
Accessibility, Clusters 1/5
GO:0005515~Protein binding
GO:0070062~Extracellular exosome
GO:2000049~Positive regulation of cell-cell adhesion mediated by cadherin
GO:0042599~Lamellar body
GO:0030877~Beta-catenin destruction complex
GO:0045334~Clathrin-coated endocytic vesicle
GO:0005615~Extracellular space
GO:0016055~Wnt signaling pathway
-log(P-value)
C
Accessibility
DKK1
HES1
BMP4
SOX9
FOXA2
MBD4
FOXA1
KLF4
GATA6
SHH
FZD4
ETV4
LGR4
ETV5
FGFR2
LAMP3
GSK3B
CDK6
FOXA2
NAPSA
SPRY2
NKX2-1
BRD4
SLC34A2
SFTPC
AXIN2
SFTPB
SFTA3
CD74
LIPE
ABHD4
APOA4
SFTPA1
APOC1
DLG4
PLCE1
VPS51
STX5
PDPN
APOLD1
ACBD3
BMPR2
7-9 pcw organoids
LinPOS organoids
Alveolar organoids
Alveoalr organoids
D
Gene region enrichment (GREAT) of NKX2.1-peaks
7-9 pcw organoids
LinPOS organoids
Alveolar organoids
Region-gene associateions
Distance to TSS (kb)
TSS
E
NKX2.1-bound, Cluster 2
Wnt signalling-associated
GO:0010628~Positive regulation of gene expression
GO:0035567~Non-canonical Wnt signaling pathway
GO:0060070~Canonical Wnt signaling pathway
GO:0016055~Wnt signaling pathway
GO:0007067~Mitotic nuclear division
NKX2.1-bound, Cluster 4
Cell cycle associated
GO:0006355~Regulation of transcription, DNA-templated
GO:0006351~Transcription, DNA-templated
GO:0007010~Cytoskeleton organization
GO:0000086~G2/M transition of mitotic cell cycle
GO:0035023~Regulation of Rho protein signal transduction
NKX2.1-bound, Cluster 8
GO:0000209~Protein polyubiquitination
GO:0006006~Glucose metabolic process
GO:0007267~Cell-cell signaling
GO:0001701~In utero embryonic development
GO:0007399~Nervous system development
-log(P-value)
F
Motif enrichment (AME); commonly enriched
NKX2.1_HUMAN.H11MO.0.A
7-9 pcw organoids
p-value=3.94e-299
LinPOS organoids
p-value=9.47e-310
Alveolar organoids
p-value=6.62e-11
% of peaks containing NKX2.1 motif
FOXP1_HUMAN.H11MO.0.A
p-value=1.24e-950
p-value=5.22e-728
p-value=1.19e-299
% of peaks containing FOXP1 motif
ZNF770_HUMAN.H11MO.0.A
p-value=1.08e-3920
p-value=2.89e-2650
p-value=5.68e-10087
% of peaks containing ZNF770 motif
G
De novo motif enrichment (AME); differentially/relatively enriched
7-9 pcw organoids
LinPOS organoids
Alveolar organoids
SMARCA5_HUMAN.H11MO.0.C
p-value = 5.33e-522
FOXP2_HUMAN.H11MO.0.C
p-value = 6.93e-164
GATA6_HUMAN.H11MO.0.A
p-value = 1.65e-155
RBPJ_HUMAN.H11MO.0.A
p-value = 4.12e-93
RARG_HUMAN.H11MO.0.B
p-value = 7.66e-66
SMARCA5_HUMAN.H11MO.0.C
p-value = 9.19e-345
TCF3_HUMAN.H11MO.0.A
p-value = 1.98e-209
FOXP2_HUMAN.H11MO.0.C
p-value = 4.95e-179
RXRA_HUMAN.H11MO.0.A
p-value = 6.73e-267
RBPJ_HUMAN.H11MO.0.A
p-value = 4.78e-107
ARID5B_HUMAN.H11MO.0.C
p-value = 4.73e-3289
CEBPG_HUMAN.H11MO.0.B
p-value = 1.17e-1417
NFKB1_HUMAN.H11MO.0.B
p-value = 6.22e-841
TCF3_HUMAN.H11MO.0.A
p-value = 2.90e-394
RXRB_HUMAN.H11MO.0.C
p-value = 1.00e-161

Figure S7. NKX2.1-DamID-seq analysis of lung tip organoids and alveolar organoids. Related to Figure 6.

(A) Heatmap illustrating k-means clustering of chromatin accessibility across the organoid samples (Dam-only). Colours represent binding intensities from genes associated with peaks, that was averaged across gene bodies, including -1kb from the TSS.

(B) GO enrichment analysis of BP-associated GO terms on the Clusters 1 and 5 showing increased binding intensity across the organoid samples.

(C) Heatmap describing the representative genes that showed relatively high chromatin accessibility for each sample.

(D) Distribution across genomic features analysed using GREAT.

(E) GO enrichment analysis of BP-associated GO terms on the Clusters 2 (Wnt signalling-associated), 4 (cell cycle-associated), and 6.

(F and G) Motif enrichment analysis performed using AME package from MEME suite. Commonly enriched (F) and differentially/relatively enriched (G) motifs were selected with *p*-value across the organoid samples.
